# Two coexisting pseudo-mirror heteromolecular telomeric G-quadruplexes in opposite loop progressions differentially recognized by a low equivalent of Thioflavin T

**DOI:** 10.1093/nar/gkab755

**Published:** 2021-09-09

**Authors:** Wenqiang Fu, Haitao Jing, Xiaojuan Xu, Suping Xu, Tao Wang, Wenxuan Hu, Huihui Li, Na Zhang

**Affiliations:** High Magnetic Field Laboratory, Chinese Academy of Sciences, Hefei 230031, China; University of Science and Technology of China, Hefei 230026, China; High Magnetic Field Laboratory, Chinese Academy of Sciences, Hefei 230031, China; University of Science and Technology of China, Hefei 230026, China; High Magnetic Field Laboratory, Chinese Academy of Sciences, Hefei 230031, China; University of Science and Technology of China, Hefei 230026, China; High Magnetic Field Laboratory, Chinese Academy of Sciences, Hefei 230031, China; High Magnetic Field Laboratory, Chinese Academy of Sciences, Hefei 230031, China; High Magnetic Field Laboratory, Chinese Academy of Sciences, Hefei 230031, China; University of Science and Technology of China, Hefei 230026, China; High Magnetic Field Laboratory, Chinese Academy of Sciences, Hefei 230031, China; University of Science and Technology of China, Hefei 230026, China; High Magnetic Field Laboratory, Chinese Academy of Sciences, Hefei 230031, China; Key Laboratory of High Magnetic Field and Ion Beam Physical Biology, Hefei Institutes of Physical Science, Chinese Academy of Sciences, Hefei 230031, China; Key Laboratory of Anhui Province for High Field Magnetic Resonance Imaging, Hefei 230031, China; High Magnetic Field Laboratory of Anhui Province, Hefei 230031, China

## Abstract

The final 3′-terminal residue of the telomeric DNA G-overhang is inherently less precise. Here, we describe how alteration of the last 3′-terminal base affects the mutual recognition between two different G-rich oligomers of human telomeric DNA in the formation of heteromolecular G-quadruplexes (hetero-GQs). Associations between three- and single-repeat fragments of human telomeric DNA, target d(GGGTTAGGGTTAGGG) and probe d(TAGGGT), in Na^+^ solution yield two coexisting forms of (3 + 1) hybrid hetero-GQs: the kinetically favourable LLP-form (left loop progression) and the thermodynamically controlled RLP-form (right loop progression). However, only the adoption of a single LLP-form has been previously reported between the same probe d(TAGGGT) and a target variant d(GGGTTAGGGTTAGGG***T***) having one extra 3′-end thymine. Moreover, the flanking base alterations of short G-rich probe variants also significantly affect the loop progressions of hetero-GQs. Although seemingly two pseudo-mirror counter partners, the RLP-form exhibits a preference over the LLP-form to be recognized by a low equivalent of fluorescence dye thioflavin T (ThT). To a greater extent, ThT preferentially binds to RLP hetero-GQ than with the corresponding telomeric DNA duplex context or several other representative unimolecular GQs.

## INTRODUCTION

Human telomeric DNA contains thousands of tandem repeats of the G-rich (GGGTTA)n sequence (1), with a 3′-end single-stranded overhang of 100–200 nucleotides (2). Under physiological ionic conditions, the G-rich sequences of human telomeric DNA can adopt a four-stranded helical structure (G-quadruplex, GQ) by the stacking of multiple G-tetrads through Hoogsteen type base pairing between four guanines, mostly in K^+^ or Na^+^ solution (3–5). Telomeric DNA GQs are likely important for telomere biology (3–9) and are a good target for drug design (3–5,9–11).

Telomeric DNA GQs are highly polymorphic (5). Their varieties of topologically distinct structures are assembled mainly by one, two, or four separate strands, and very occasionally by three strands, under different experimental conditions (3–5,12,13). Formation of unimolecular GQs folded by the various individual telomeric DNA strands has been more widely observed. Other intermolecular GQs, mostly dimeric or tetrameric, are formed by the association of two or four telomeric DNA strands, respectively, containing two- or single- repeat fragments. Notably, through the homomolecular association between multiple strands of the same kind with identical sequences, almost all intermolecular GQs preferentially self-assemble. Rarely, a hetero-GQ assembly can consist of quite different G-rich oligomers (5).

The three-repeat human telomeric sequence d(GGGTTAGGGTTAGGG***T***) (***htel3*** in Table 1) associates with the single-repeat human telomeric sequence d(TAGGGT) to form a heteromolecular GQ assembly (14). Telomerase catalyses the elongation of single-stranded telomeric DNA. The formation of hetero-GQs will be expected to inhibit telomerase. As an alternative to the conventional antisense method using a complementary oligomer, the heteromolecular GQ assembles between two different G-rich oligomers, with the longer one as the target captured by a short G-rich probe. This could be a novel way to recognize G-rich sequences of telomere or other critical genomic regions (14,15). Mutual recognition between two different molecules plays a central role in organisms. The capacity for intermolecular recognition between two G-rich DNA oligomers has been studied. However, heteromolecular GQ (hetero-GQ) complexes assembled by other G-rich DNA oligomers of different lengths have remained largely unexplored.

**Table 1.** Sequence of natural and sequence-specific labeled oligonucleotides

Name	Sequence (5′-3′)	Abundance
***htel3***	GGGTTAGGGTTAGGGT	Natural abundance
***htel3* *Δ* *T***	GGGTTAGGGTTAGGG	Natural abundance
***htel3M1***	GGGTTAGGGTTAGGGTT	Natural abundance
***htel3M2***	GGGTTAGGGTTAGGGTTA	Natural abundance
***htel3M3***	GGGTTAGGGTTAGGGTTAG	Natural abundance
***htel3M4***	GGGTTAGGGTTAGGGTTAGG	Natural abundance
***P1***	TAGGGT	Natural abundance
***P2***	GGGTTA	Natural abundance
***P3***	TTAGGG	Natural abundance
***htel3* *Δ* *T-C***	CCCTAACCCTAACCC	Natural abundance
***htel3* *Δ* *T-G1***	**G** GGTTAGGGTTAGGG	4%
***htel3* *Δ* *T-G2***	G**G**GTTAGGGTTAGGG	4%
***htel3* *Δ* *T-G3***	GG**G**TTAGGGTTAGGG	4%
***htel3* *Δ* *T-G7***	GGGTTA**G**GGTTAGGG	4%
***htel3* *Δ* *T-G8***	GGGTTAG**G**GTTAGGG	4%
***htel3* *Δ* *T-G9***	GGGTTAGG**G**TTAGGG	4%
***htel3* *Δ* *T-G13***	GGGTTAGGGTTA**G**GG	4%
***htel3* *Δ* *T-G14***	GGGTTAGGGTTAG**G**G	4%
***htel3* *Δ* *T-G15***	GGGTTAGGGTTAGG**G**	100%
***P1-G24***	TA**G**GGT	100%
***P1-G25***	TAG**G**GT	100%
***P1-G26***	TAGG**G**T	100%
***P2-G24***	**G** GGTTA	100%
***P2-G25***	G**G**GTTA	100%
***P2-G26***	GG**G**TTA	100%
***P3-G24***	TTA**G**GG	100%
***P4-G25***	TTAG**G**G	100%
***P3-G26***	TTAGG**G**	100%

The bold and underlined **G** indicates that this residue is labelled with ^13^C, ^15^N isotope.

The natural abundances of ^13^C and ^15^N are 1.11% and 0.37%, respectively.

The different folding topologies of hetero-GQ assemblies could be related to the distinct recognition modes between the two different G-rich fragments. Notably, alteration in the looped or overhung non-G nucleotides flanking to the G-rich core segment of the GQ-forming sequence can cause significant structural variation. This occurs frequently among unimolecular GQs assembled by four-repeat human telomeric sequences (16–21). Whether this structural variation due to non-G flanking base variants is also applicable to the formation of other heteromolecular GQ assemblies is unclear.

Only the single-stranded form of the short fragment at the extreme 3′-end of G-overhang serves as a suitable substrate for telomerase. Theoretically, GQs can form anywhere along the long G-rich overhang of telomeric DNA. However, telomeric GQs form preferentially at the extreme 3′-end, rather than at the internal positions, of long telomeric DNA G-overhangs (22). This preference may contribute to the inhibition of telomerase elongation activity (14,22). Additionally, the last residue at the extreme 3′-end of the telomeric DNA G-strand is heterogeneous in base composition, without precise ending (23). These features make this particular location even more appealing. We describe here the nuclear magnetic resonance (NMR) investigation of how an alteration in the last 3′-end base affects the mutual recognition between two different G-rich fragments of human telomeric DNA in the formation of heteromolecular G-quadruplexes (hetero-GQs).

**Figure 1.**
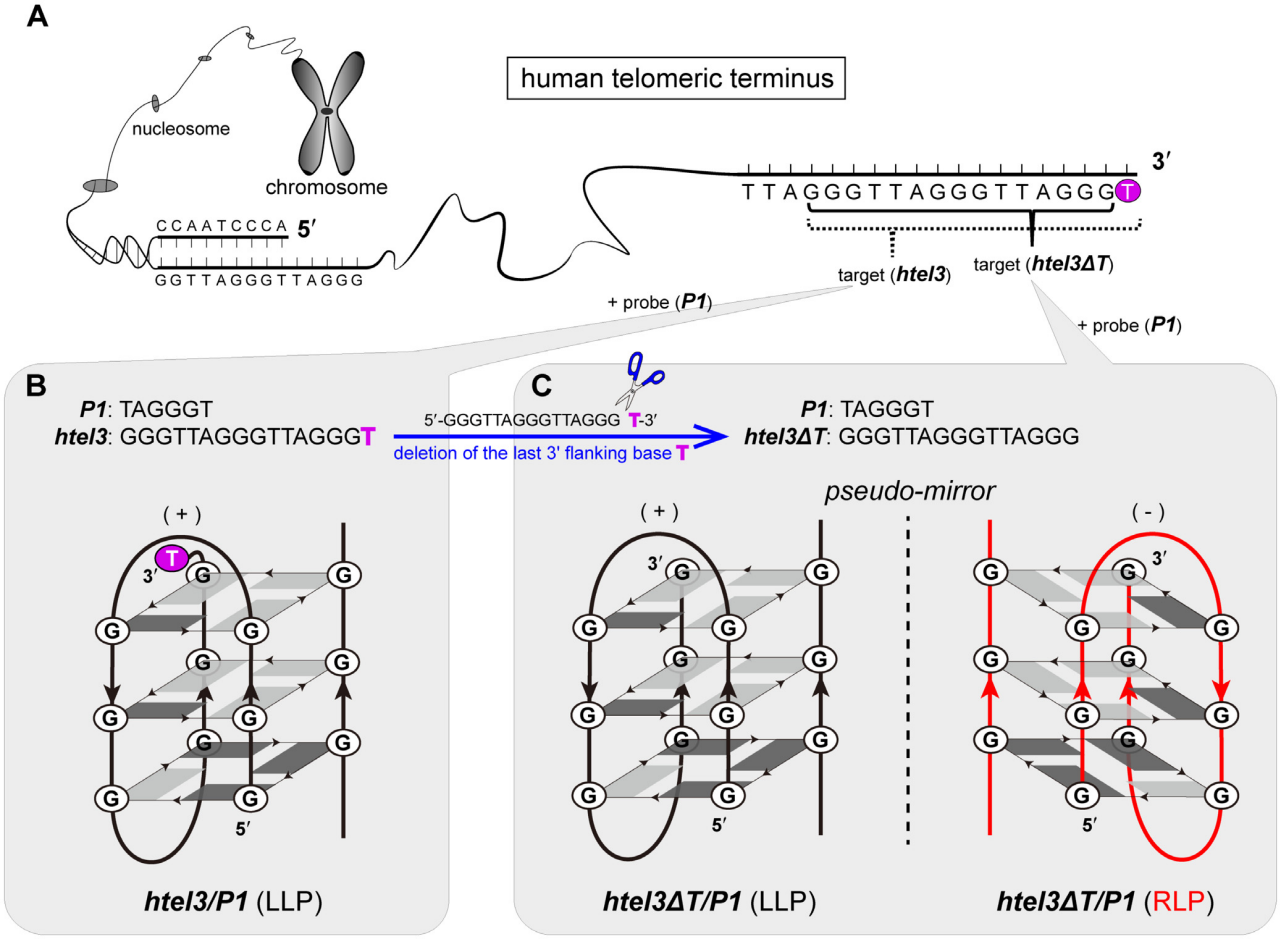
A single alteration of the last 3′ terminal nucleotide flanking to the core segment of G-rich sequence dramatically influences the association between the three-repeat target and the single-repeat probe of human telomeric DNA fragments in the assembly of different (3 + 1) hybrid hetero-GQs. (**A**) Schematic diagram of the location and sequences of target ***htel3*** and target ***htel3ΔT*** at the 3′ terminus of human telomere. Sequence ***htel3ΔT*** has only one fewer 3′-end thymine than the previously reported sequence of ***htel3*** (14). The last extra single thymine at 3′-end of ***htel3*** is highlighted in magenta. (**B**) Complex ***htel3***/***P1*** adopts only a single (3 + 1) hybrid GQ assembly with left loop progression (LLP) (14). The last extra thymine at 3′-end of ***htel3*** is shown in bold magenta. (**C**) Complex ***htel3ΔT***/***P1*** adopts two coexisting (3 + 1) hybrid GQs in opposite loop progressions seemingly as pseudo-mirror counter partners: the kinetically favourable LLP-form (backbones are coloured in black with left loop progression) and the thermodynamically controlled RLP-form (backbones are coloured in red with right loop progression). LLP is also indicated by (+) and RLP is indicated by (-), as defined previously (78). *Syn*- and *anti*-guanines are coloured in dark grey and light grey, respectively.

When the last three-repeat fragment at the extreme 3′-end of human telomeric DNA G-overhang serves as a designated target, its core segment of the sequence may or may not end with thymine (Figure 1A). The target sequence d(GGGTTAGGGTTAGGG) of this study (***htel3ΔT*** in Table 1) has only one less 3′-end thymine than the previously reported sequence variant of d(GGGTTAGGGTTAGGG***T***) (***htel3*** in Table 1) (14). Despite this minor deviation, ***htel3ΔT*** and ***htel3*** exhibited quite different behaviours (Figure 1B and C) when each was associated with the same short G-rich probe of d(TAGGGT) (***P1*** in Table 1).

A previous report (14) adopted only a single hetero-GQ assembly with a (3 + 1) hybrid topology in the complex between ***htel3*** and ***P1*** in Na^+^ solution (Figure 1B). This (3 + 1) hybrid hetero-GQ of ***htel3***/***P1*** has three parallel and one antiparallel strand orientations, three stacked G-tetrads, and two TTA edgewise loops in the left progression (Figure 1B).

In contrast, we found that the association between ***htel3ΔT*** (one fewer 3′-end thymine than ***htel3***) and the same ***P1*** in Na^+^ solution led to the formation of two different hetero-GQ assemblies coexisting in equilibrium: a kinetically favourable GQ and a thermodynamically controlled GQ. These coexisting ***htel3ΔT***/***P1*** GQs adopted the same (3 + 1) hybrid strand orientation, but in completely opposite loop progressions. The kinetic hetero-GQ with a faster assembly rate is termed the left loop progression (LLP) form. The thermodynamic hetero-GQ with slower assembly is termed the right loop progression (RLP) form (Figure 1C). These findings reveal that a flanking nucleotide alteration, even only one less flanking thymine at the 3′-end, has a significant impact on the mutual recognition between two different G-rich oligomers.

Interestingly, both the LLP- and RLP-forms of ***htel3ΔT***/***P1*** share many structural similarities. These include the same (3 + 1) hybrid strand orientation with two TTA edgewise loops and three layers of G-tetrads, the same G-residue arrays to construct each individual G-tetrad, and the same *syn* and *anti* glycosidic angle patterns of guanine residues along the sequence and in the G-tetrads (Figure 1C). However, the forms have completely opposite loop progression. These two hetero-GQs of the LLP- and RLP-forms might at first seem to be pseudo-mirror counter partners with a particular emphasis on the global folding topology (Figure 1C).

Although they exhibit less dramatically distinct structural dissimilarities, can LLP- and RLP-form GQs be selectively recognized by a ligand? Remarkably, even in a mixture of both forms coexisting in equilibrium, the fluorescence dye thioflavin T (ThT), which is much less molar equivalent, preferentially recognized the RLP-form over the LLP-form of hetero-GQs. To a greater extent, ThT also preferentially bound to RLP hetero-GQ than with the corresponding telomeric DNA duplex context or several other representative intramolecular GQs.

Our findings are expected to have important implications for the structure of telomeric DNA and targeting GQs in telomeres. Forming hetero-GQs of telomeric DNA will inhibit telomerase which plays an important role in cancer and aging. Even a subtle alternation of flanking non-G residues can significantly affect the folding of these hetero-GQs that subsequently renders different responses to the interactions of small molecule ligands or other GQ-specific proteins.

## MATERIALS AND METHODS

### DNA sample preparation

The unlabelled oligonucleotides purchased from Sangon Biotech (Shanghai) Co., Ltd. (China) were purified by high-performance liquid chromatography (HPLC) using a C18 reversed-phase separation column. Sequence-specific low-enriched (4% uniformly ^13^C,^15^N-labelled) oligonucleotides (24) were synthesized by TaKaRa Biomedical Technology Co., Ltd. (China). Labelled phosphoramidites of 2′-deoxyguanosine (^13^C10, 98%; ^15^N5, 98%) were purchased from Cambridge Isotope Laboratories, Inc. (USA). Alternatively, using our in-house enzymatic synthesis approach (12), we also prepared 100% ^13^C,^15^N-labelled oligonucleotides specifically at G15 of target sequence ***htel3ΔT***, as well as at G24, G25, and G26 positions of probes ***P1***, ***P2***, and ***P3***, respectively (Table 1). All oligonucleotides designed as corresponding primers/templates (listed in Supplementary Table S2) were chemically synthesized by Sangon Biotech (Shanghai) Co., Ltd. The 100% uniformly ^13^C,^15^N-labelled deoxyribonucleosidetriphosphates (dNTPs) used in enzymatic labelling approach were purchased from Cambridge Isotope Laboratories, Inc. (USA).

The concentration of ThT was calibrated with standard concentrations of dGTP and dTTP purchased from Sangon Biotech Co., Ltd. (Shanghai). The DNA strand concentration was determined by measuring the UV absorbance at 260 nm. DNA samples with a millimolar strand concentration were prepared in 100 mM sodium chloride and 20 mM sodium phosphate buffer (pH 6.8). The sample was heated for 5 min in 50 ml boiling water above 95°C and then slowly annealed as the water returned to room temperature (termed the annealing procedure). Next, the samples were dialysed in 100 mM sodium chloride and 20 mM sodium phosphate buffer (pH 6.8) to remove contaminants before examination. Subnanomolar DNA samples were prepared by diluting the prepared millimolar samples with 100 mM sodium chloride and 20 mM sodium phosphate buffer (pH 6.8), unless otherwise specified. Moreover, the samples were heated at 95°C for 5 min, and then cooled by quickly placing them in a 75% alcohol bath at -20°C (termed the quench procedure). The samples for NMR measurement in D_2_O were prepared by freeze-drying and re-dissolving in 99.96% D_2_O.

### Circular dichroism (CD) spectroscopy experiment

The CD spectra of low concentration samples (50 μM) were obtained at room temperature (∼25°C) using a model J-810 circular dichroism spectrometer (JASCO, Japan). Spectra from 200 to 340 nm were recorded at a scanning rate of 100 nm/min. The CD measurements of high concentration samples (0.5 mM) were carried out on an Applied Photophysics Chirascan circular dichroism spectrometer (25). All measurements were made in a 1-mm path length quartz cuvette. Induced Circular Dichroism (ICD) spectra were recorded between 200 and 550 nm during the titration of GQs with ThT (26). To ensure an equivalent association ratio between a short G-rich probe and the G-rich oligonucleotide target, folding status of hetero-GQ complexes was first examined by NMR. These NMR examined samples were then diluted to 40 μM in 200 μl of 5 mM sodium phosphate buffer containing 100 mM NaCl for CD measurements. An average of three scans was taken for each measurement and the baseline was corrected using the same buffer.

### Melting temperature measurement of UV-Vis spectroscopy

The G-quadruplex was evaluated by UV-vis melting experiments measured at 295 nm using a METASH UV-6100 UV-vis spectrometer (Shanghai Metash Instruments Co., Ltd. China). Gradual heating was performed from 20 to 75°C at a rate of 0.5°C/min, as described previously (27). UV-vis spectra were recorded at 1°C intervals. A buffer melting baseline was subtracted from each spectrum and the data was normalized. The melting temperature (Tm) was defined as the midpoint of the transition calculated by the first derivative of the sigmoidal curve. This midpoint corresponds to a population of a 50% folded and 50% unfolded G-quadruplex structure (28,29). The melting curve was analysed and fit to the logistic function $( {y = {A_2} + \frac{{{A_1} - {A_2}}}{{1 + {{(X - {X_0})}^P}}}} )$ in Origin 8.5.1 software. Tm was equal to *X*_0_, and other related fitting parameters were listed in Supplementary Figure S15. The pre-examined 3 mM NMR samples were diluted to 85 μM in 500 μL of 20 mM sodium phosphate buffer containing 100 mM NaCl for UV measurements. The measurements were repeated three times, and the average value was obtained.

### NMR spectroscopy

NMR data were collected on 500, 600 and 850 MHz Bruker spectrometers with cryoprobes at 288 and 303 K. Two-dimensional total correlation spectroscopy (TOCSY) with a mixing time of 80 ms, ^1^H-^13^C heteronuclear multiple bond correlation (HMBC), ^1^H-^15^N heteronuclear single quantum correlation (HSQC), ^1^H-^13^C HSQC, and nuclear Overhauser effect spectroscopy (NOESY) spectra in H_2_O (mixing times of 250 ms) and D_2_O (mixing times of 50 ms and 250 ms) were recorded for resonance assignment and structural identification. Water suppression was used in experiments either by gradient-tailored excitation (WATERGATE) for 10% D_2_O samples or pre-saturation pulse sequence for 100% D_2_O samples. All datasets were processed and analysed using Bruker Topspin 3.6.2. and CcpNmr Analysis software (version 2.4.2) (30).

### NMR structure calculation

The distance restraints of non-exchangeable protons were derived from the ^1^H-^1^H NOESY spectrum in 100% D_2_O (250 ms mixing time) processed by CcpNmr Analysis 2.4.2 (30). The process is based on the *r*^-6^ distance dependence of NOE (31) and involves using the peak intensity (volume) corresponding to a known distance, for calibration. The distance of independent H1′-H2″ cross peak was set to 2.20 Å as the distance reference (32). Boundaries of ± 20% up to ± 30% were used for the measured volume integration. The exchangeable proton restraints were estimated from the NOESY spectrum (mixing time of 250 ms) in H_2_O. These distances were classified as strong 2.7 (±0.9) Å, medium 3.8 (±1.2) Å, or weak 5.0 (±1.5) Å according to the intensities of cross peak volumes in the NOESY spectrum, respectively. Twelve hydrogen-bond restrains were added to retain hydrogen-bonding within the individual G-tetrads. The glycosidic χ torsion angles of *syn* and *anti* bases showed strong and medium H1′-H8/H6 cross peaks in the 50 ms NOE spectrum, which were set to 65° ± 25° and 220° ± 30°, respectively (15).

The structures of ***htel3ΔT***/***P3*** were determined by MD simulated annealing computations driven by NOE distance, dihedral angle, and hydrogen-bonding restraints by using the XPLOR-NIH program (version 3.0.3) as previously described (33–35). First, iterative distance geometry simulated annealing was performed. A set of folded structures was generated from an extended conformation with satisfactory covalent geometry by enforcing distance, torsion angle, and planarity restraints following the protocol as previously reported (36,37). The force constants were scaled at 1 and 200 kcal·mol^-1^·Å^-2^ for distance restraints and hydrogen-bond constraints. The unambiguous restraints were first incorporated. The best structure with the lowest energy and an acceptable number of violations was used as the structure for the next round of refinement. In this manner, an increasing number of NOE distance restraints were included in the structure calculations. Further, distance-restrained MD refinement was performed (38). The best structure based on both their minimal energy terms and number of NOE violations from the first step was input as a start file for refinement. Two hundred structures were generated. In this step, dihedral (50 kcal·mol^-1^·rad^-2^) and planarity (0.6 kcal·mol^-1^·Å^-2^ for tetrads) restraints were maintained throughout the course of refinement. A total of 405 distance restraints were used in the structural calculations. Eventually, the ten best refined structures based on the lowest energy and the lowest number of NOE violations were selected as the representative solution structures of hetero-GQ ***htel3ΔT***/***P3***. Subsequently, these ten best representative solution structures were validated by the wwPDB validation system and deposited in the Protein Data Bank (PDB ID: 7DO1). The refined structures were displayed using the PyMOL (39). The chemical shifts have been deposited in BioMagResBank under the accession code 50625.

### Molecular docking

Chemical shift perturbation experiments and ICD experiments indicated that ThT was stacked on the G1·G9·G13·G24 tetrad of ***htel3******ΔT***/***P3*** (Figure 7E and G; Supplementary Figures S29C and S30B). In order to obtain the refined structure of ThT-***htel3ΔT/P3*** complex, molecular docking calculations were carried out using the Schrodinger software (Maestro 11.5, Schrodinger 2018-1) (40,41). First, the initial structure of ***htel3ΔT/P3*** (PDB ID: 7DO1), as the receptor of docking input coordinate file, was prepared by XPLOR-NIH. During this process, inter-residue NOE restraints of the T10T11A12 loop and T21T22A23 overhang adjacent to the G1·G9·G13·G24 tetrad were removed to make the binding sites accessible to ThT (Figure 7H). Then, the receptor optimized using the wizard tool in Maestro panel. OPLS_2005 force field was applied to minimize the structure of the receptor (42). Further receptor grid boxes were generated using ‘Receptor Grid Generation’ module at the binding site (the center of residue G1, G13, and G24) of receptor with the computing cubic box of 20 Å × 20 Å × 20 Å. The initial coordinate file of ligand ThT was obtained by Chemoffice 2014. 3D and geometry optimizations with energy minimization of ligand were executed using algorithms monitored in Schrodinger software (40). ‘LigPrep’ module was used to generate 3D structures of the ligand by adding hydrogen atoms and removing salt and ionizing at pH (7.0 ± 2) (43). Energy minimization was performed using OPLS_2005 force field by using the standard energy function of molecular mechanics and RMSD cut off 0.01 Å to generate the low-energy ligand tautomer (41). Finally, the ligand ThT was docked to the binding site of receptor using extra precision (XP). Concluding energy assessment was done with the docking score. The perfect complex (ThT-***htel3ΔTP3***) with the lowest docking score was selected for further molecular dynamics simulation to obtain the more refined structure.

### MD simulation

The molecular dynamics (MD) simulations of ThT-***htel3ΔT/P3*** complex were performed using Amber 18 software (44). In this process, not any NOE restraint was incorporated. Topology of the G-quadruplex was derived from AmberTool (‘tleap’) with the choice of the parmbsc1 force field (45), a refined force field for DNA simulations. The partial charges of atoms on ThT were calculated by the DFT method and then derived from RESP fitting (46) via the AmberTool ‘antechamber’ (47). The force field used for the ThT was the generalized Amber force field (GAFF) (48). The system was solvated using the TIP3P water model, which was extended up to 10 Å in a hexahedron box. The neutrality of the system was ensured by the addition of Na^+^ ions. The periodic boundary condition was turned on, with a 12 Å cutoff for both electrostatic and van der Waals interactions. The steepest descent algorithm for the first 9000 steps and the conjugate gradient algorithm for the last 1000 steps were performed during the energy minimizations. The solvents and ions were equilibrated under an NVT ensemble (298 K), followed by an NPT ensemble (1 atm). The simulation time for NVT and NPT equilibrations was 200 ps with an integration time step of 2 fs (hydrogen atoms were constrained using the shake algorithm). A Langevin thermostat was used to control the temperature. The random number generator was initialized with a random seed. The coordinates in the mdcrd trajectory file were updated every 1000 steps during the production MD run. The length of the production MD run was 150 ns, which was repeated three times in parallel. Analyses of the resulting trajectories were performed using the Ptraj module of Amber 18, and the VMD program (49) was used for visualization.

## RESULTS AND DISCUSSION

### Formation of two coexisting hetero-GQs assembled between three-repeat *htel3ΔT* and single-repeat *P1* of human telomeric DNA sequences

**Figure 2.**
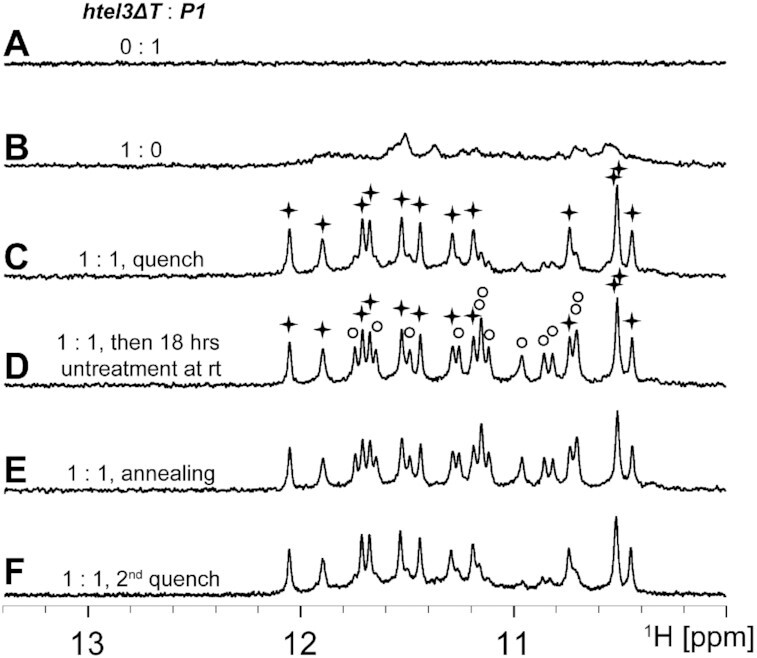
NMR detection of the formation of two coexisting hetero-GQs in an equimolar mixture of ***htel3ΔT*** and ***P1*** under different experimental conditions. The expanded imino proton region of one-dimensional ^1^H spectra of ***P1***alone (**A**), ***htel3Δ******T***alone (**B**), ***htel3ΔT***/***P1*** hetero-GQ complex at an equimolar ratio under different conditions: quick quench (**C**), quick quench followed by 18 hours untreated incubation at room temperature (**D**), slow annealing initially (**E**), and slow annealing followed by a second quench (**F**). The imino proton peaks of the LLP- and RLP-forms of ***htel3ΔT***/***P1*** hetero-GQs are denoted by asterisks and circles, respectively. The spectra were collected at 288 K in a solution with a 0.4 mM DNA strand concentration, 20 mM Na-Pi buffer (pH 6.8), and 100 mM NaCl.

The d(TAGGGT) of ***P1*** alone was essentially unstructured, with no hydrogen-bonded imino proton signals observed at 10–12 ppm (Figure 2A). The d(GGGTTAGGGTTAGGG) of ***htel3ΔT*** alone mostly self-assembled into a mixture of conformationally heterogeneous GQs, as indicated by the presence of multiple sets of imino peaks at 10–12 ppm (Figure 2B), the characteristic ^1^H NMR region for GQ formation. Increased ***P1*** during titration gradually lessened and eliminated the original NMR signals of ***htel3ΔT***. For an equimolar mixture of ***htel3ΔT*** and ***P1***, a clean new single set of 12 imino proton signals at 10–12 ppm developed after quick quenching (asterisks in Figure 2C). These 12 imino proton signals were well-resolved and sharp, suggesting the formation of a hetero-GQ assembly between ***htel3ΔT*** and ***P1***, which likely contains three layers of G-tetrads.

Starting from this asterisked form as essentially the only populated GQ form after rapid quenching, spontaneously another new single set of signals (circled in Figure 2D) gradually increased during untreated incubation of samples on the bench at room temperature (∼25°C); meanwhile the signals of asterisked form partially decreased. After 18 h, two sets of guanine imino proton peaks, comprising ∼60% and 40% of the total, appeared at 10–12 ppm (asterisk and circle, respectively, in Figure 2D). The observations indicated the coexistence of two structurally distinct GQ complexes in the equimolar mixture of ***htel3ΔT*** and ***P1***.

Alternatively, when the equimolar mixture of ***htel3ΔT*** and ***P1*** was initially treated by slow annealing, the ultimate equilibrium in the population was also reached with 60% and 40% of each form (asterisk and circle, respectively, in Figure 2E). The spectra of this equimolar mixture in both Figure 2D and E were very similar to each other. Both had a kinetically favourable GQ form (indicated by the asterisk) and another thermodynamically controlled GQ form (indicated by the circle), indicating that both GQs coexisted in equilibrium. On the other hand, only the kinetically favourable GQ was present in the quick quenching condition (Figure 2C).

When a prior equilibrium mixture of two coexisting forms of the GQ forms indicated above by the asterisk and circle were quenched again, the kinetically favourable asterisked GQ form reappeared as the only major folded GQ structure (Figure 2F). Its spectrum was identical to that of Figure 2C. These observations suggest that the kinetically favourable and thermodynamically controlled GQ forms are mutually inter-convertible through global unfolding/refolding processes of these hetero-GQ complexes. The controllable folding form feature for this equimolar mixture of ***htel3ΔT*** and ***P1*** advantageously facilitated subsequent NMR spectrum assignments and structural identification that both are fully described below. The kinetically favourable GQ represented the LLP-form and the thermodynamically controlled GQ represented the RLP-form. The folding and unfolding kinetics of these forms of the ***htel3ΔT***/***P1*** GQ complex are detailed in the supplementary text and Supplementary Figures S1 and S2.

### NMR spectral assignments of LLP-form and RLP-form of *htel3ΔT*/*P1* hetero-GQs

Non-ambiguous resonance assignments are usually performed by a sequence-specific low enrichment labelling approach, using samples that are generally 2–8% isotopically labelled at a designated position by solid-phase chemical synthesis (24). However, this chemical approach is currently unable to isotopically label the last residue at the extreme 3′-end of DNA oligonucleotides. Except for the last 3′-end G15, we labelled every other guanines in the ***htel3ΔT*** sequence of d(G1G2G3T4T5A6G7G8G9T10T11A12G13G14G15) with a 4% ^13^C,^15^N-low enrichment by solid-phase chemical synthesis. Alternatively, the last 3′-end G15 of ***htel3ΔT*** was specifically 100% ^13^C,^15^N-labelled using our in-house enzymatic approach at a very affordable price, as described in the labelled sample preparation in the Methods section. Similarly, other guanine-labelled samples for all short G-rich probes (***P1***, ***P2*** and ***P3***; Table 1) were also prepared using our in-house enzymatic approach. The sequence position and isotopic abundance for each of the ^13^C- and ^15^N-labelled samples, specifically at a designated single guanine residue, are listed in Table 1.

**Figure 3.**
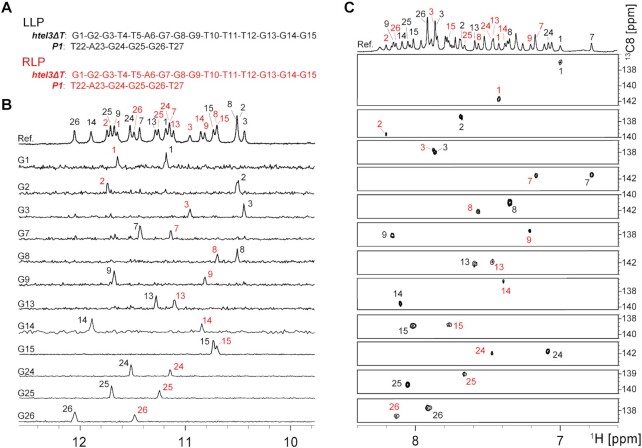
Unambiguous resonance assignments for guanine imino and aromatic protons of the LLP-form and RLP-form of ***htel3ΔT***/***P1***by using sequence-specifically ^15^N,^13^C-labelled DNA oligomers at the indicated positions. (**A**) The residue numberings of ***htel3ΔT***/***P1*** are shown. Imino (**B**) and aromatic (**C**) protons spectra with assignments indicated over the reference spectrum (Ref.). Guanine imino and aromatic protons were assigned in ^15^N-filtered and ^13^C-filtered spectra of samples that were 4% ^15^N, ^13^C-labelled at the G1, G2, G3, G7, G8, G9, G13 and G14 positions, while 100% ^15^N,^13^C-labelled at the G15, G24, G25 and G26 positions. LLP-form and RLP-form of ***htel3ΔT***/***P1*** are coloured black and red, respectively.

The imino and H8 protons of guanines were unambiguously assigned to their positions in the ***htel3ΔT*** sequence of d(G1G2G3T4T5A6G7G8G9T10T11A12G13G14G15) and in the ***P1*** sequence of d(T22A23G24G25G26T27), based on the relatively enhanced intensity in the ^15^N-filtered and ^13^C-filtered spectra, respectively (Figure 3). Notably, each sample of singly labelled guanines exhibited two intense ^15^N-filtered guanine imino proton peaks (Figure 3B). One peak corresponded to the LLP-form with a slightly higher intensity (in black). The other peak corresponded to the RLP-form with a slightly lower intensity (in red). Similarly, two intense ^13^C-filtered cross peaks of guanine H8 protons (Figure 3C) were also detected for each singly labelled guanine. Collectively, these observations are consistent with the presence of two kinds of distinctly folded hetero-GQs: the black LLP-form in ∼60% of the population and the red RLP-form in ∼40% of the population. These forms coexisted in equilibrium in an equimolar mixture of ***htel3ΔT*** and ***P1***. The black and red signals of these two hetero-GQs were readily distinguishable because the black signals of the kinetically favourable LLP-form emerged much faster than those of the thermodynamically controlled RLP-form denoted in red.

**Figure 4.**
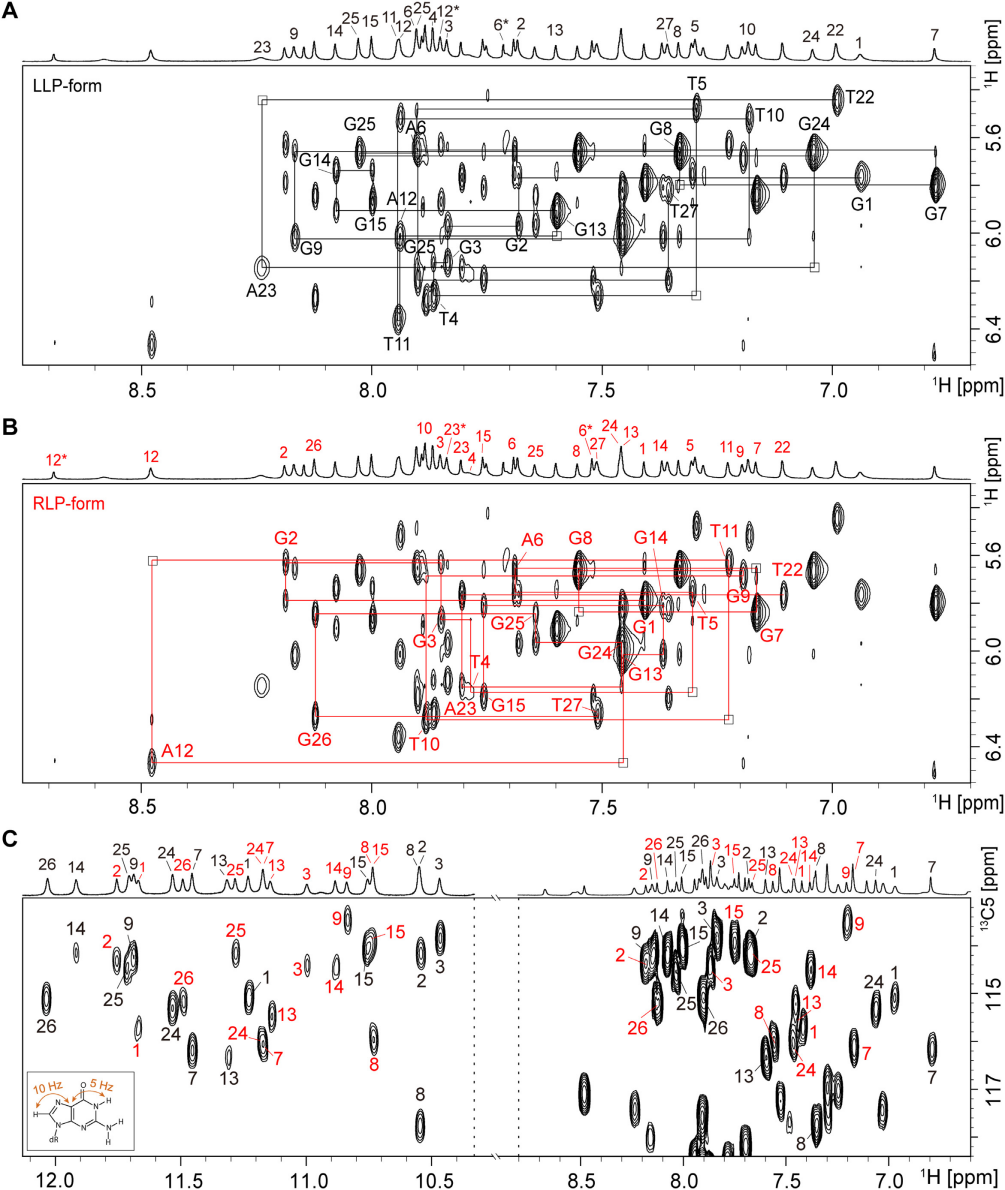
NMR spectral assignments for coexisting LLP- and RLP-forms of ***htel3ΔT***/***P1***by using conventionally non-labelled oligomers. Sequential walking in the NOESY spectrum (250 ms mixing time, D_2_O) demonstrates the H8/H6-H1′ connectivity of LLP-form (**A**) and RLP-form (**B**) of ***htel3ΔT***/***P1***. Cross peaks are labelled with residue numbers. Weak or missing sequential connections are labelled with rectangles. The H2 protons of adenine are marked by right superscript *. (**C**) Through-bond correlations between guanine imino and H8 protons via ^13^C5 at natural abundance, using long-range J-couplings, as shown in the inset. The LLP-form and RLP-form of ***htel3ΔT***/***P1***are coloured black and red, respectively.

Alternatively, we used non-isotopically labelled samples to perform a series of through-bond [2D ^1^H-^15^N HSQC (Supplementary Figure S3A), ^1^H-^13^C HSQC (Supplementary Figure S3B), ^1^H-^1^H TOCSY (Supplementary Figure S3C), and ^1^H-^13^C HMBC (Figure 4C)] and through-space [^1^H-^1^H NOESY (Figure 4A and B)] NMR experiments to gain more insight into the complex ***htel3Δ******T***/***P1***. First, non-exchangeable base H8/H6 and sugar H1′ proton assignments were accomplished by tracing the sequential NOE connections for non-isotopically labelled d(G1G2G3T4T5A6G7G8G9T10T11A12G13G14G15) of ***htel3ΔT*** and d(T22A23G24G25G26T27) of ***P1***, respectively, corresponding to the coexisting LLP-form (Figure 4A) and RLP-form (Figure 4B), in the NOESY spectrum with a mixing time of 250 ms in D_2_O solution of Na-Pi buffer (pH 6.8). Furthermore, the guanine imino proton assignments for these two individual strands of ***htel3ΔT*** and ***P1*** in the coexisting LLP-form and RLP-form were achieved by the ^1^H-^13^C HMBC experiment in H_2_O solution of Na-Pi buffer (pH 6.8) (Figure 4C). This ^1^H-^13^C HMBC experiment was based on the correlation between guanine base H8 and imino H1 protons through ^13^C5 at natural abundance (50). Overall, these assignments using non-labelled samples are consistent with those assigned non-ambiguously using labelled samples.

### NMR structural determination of LLP- and RLP-forms of *htel3ΔT*/*P1* hetero-GQs

**Figure 5.**
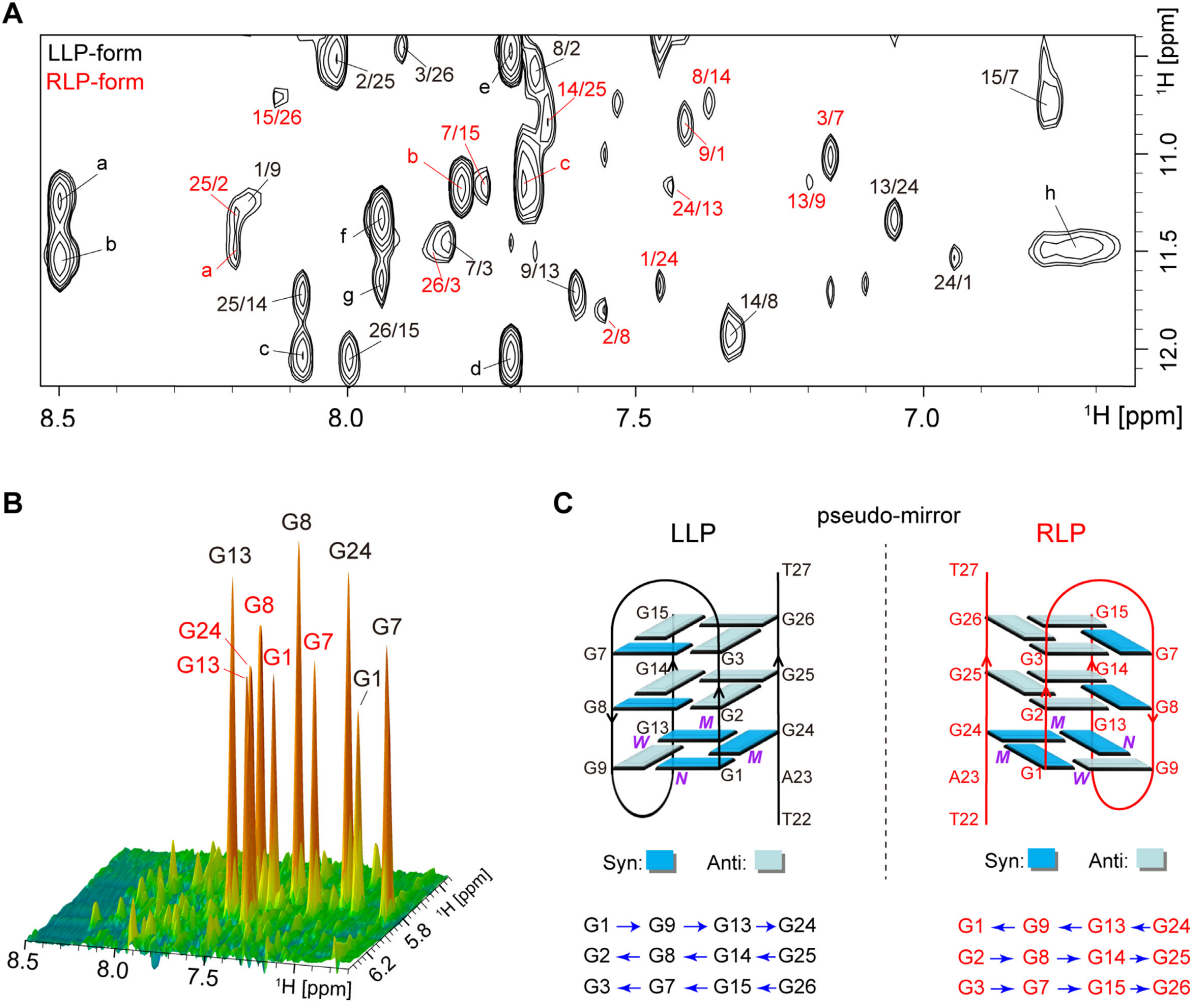
Folding topology of ***htel3ΔT***/***P1*** determined by NMR. (**A**) NOESY spectrum (250 ms mixing time, 10% D_2_O/90% H_2_O) reveals inter-residue imino H1-aromatic H8 cross peaks for the identification of the arrangement of the G-tetrads. The guanine H1-H8 cross peaks are labelled with residue numbers of the H1 and H8 protons in the first and second positions, respectively. The LLP-form and RLP-form of ***htel3ΔT***/***P1*** are coloured black and red, respectively. The remaining strong cross peaks in the NOE spectrum are also identified as: a (black), G1H1/A23H2; b (black), G24H1/A23H2; c (black), G26H1/G14H8; d (black), G26H1/A6H2; e (black), G3H1/A6H2; f (black), G13H1/A12H8; g (black), G9H1/A12H8; h (black), G7H1/G7H22; a (red), G26H1/G2H8; b (red), G24H1/A23H8; c (red), G7H1/A6H8. (**B**) Stacked plot of two-dimensional NOESY spectrum (50 ms mixing time, D_2_O) of ***htel3ΔT***/***P1*** at 288 K. Strong intra-residue H8-H1′ cross peaks for the *syn* guanines are evident. (**C**) Schematic folding topologies of ***htel3ΔT***/***P1***. The backbones of the LLP- and RLP-forms are shown as black and red solid lines, respectively. The hydrogen-bond directionality from donor (arrow tail) to acceptor (arrow head) within the same G-tetrad is shown in the same row by the blue arrows. *W*, *M*, and *N* represent wide, medium, and narrow groove width, respectively. *Syn-* and *anti-*guanines are indicated by dark blue and light blue rectangles, respectively.

Hydrogen-bond alignments and directionality within each G-tetrad were determined based on imino-H8 connections in the NOESY spectrum with a 250 ms mixing time in H_2_O (LLP-form: G1→G9→G13→G24, G2←G8←G14←G25, and G3←G7←G15←G26 in black in Figure 5C; RLP-form: G1←G9←G13←G24, G2→G8→G14→G25, and G3→G7→G15→G26 in red in Figure 5C). The hydrogen-bonding directionality is indicated by an arrow from a donor (arrow tail) to an acceptor (arrow head). Accordingly, the LLP- and RLP-forms both adopted a (3 + 1) hybrid strand orientation folding topology, with three G-tetrad layers and two edgewise TTA loops. However, the two target sequences of ***htel3ΔT*** displayed completely opposite loop progressions (Figure 5C). Notably, compared to any two given G-tetrads of the same layer corresponding to both forms, the same guanine composite arrays, same *syn* and *anti* glycosidic angle patterns of guanine residues, but exactly opposite H-bonding directionality were observed (Figure 5C). These two hereto-GQs of the LLP- and RLP-forms might seem to be pseudo-mirror counter partners, if only the whole GQ global folding topology was considered (Figure 1C).

Nevertheless, unlike those of authentic enantiomer pairs, these two GQs represent two closely related diastereomers that are both right-handed four-stranded helical structures (Supplementary Figure S4). In fact, the LLP- and RLP-forms are not even approximately superimposable, but rather are distinct in their loop progression, groove width for each loop span, and donor-acceptor relationship for each individual hydrogen-bonded pair in the G-tetrads (Figures 5C and Supplementary Figure S4).

In the stacked NOESY spectrum with a short (50 ms) mixing time in 100% D_2_O solution, two sets of strong H8-H1′ cross peaks were observed for G-residues adopting a *syn* glycosidic conformation. They included G1, G7, G8, G13 and G24 for either the LLP- or RLP-forms (Figure 5B). The adoption of a *syn* glycosidic conformation for these particular G-residues was consistent with the schematic (3 + 1) hybrid GQ topologies for both forms (Figure 5C). Furthermore, results of circular dichroism (CD) spectra and NMR hydrogen-deuterium exchange experiments supported these two (3 + 1) hybrid GQ folding topologies for both forms (the supplementary text and Figures S5 and S6).

### Similar thermal stabilities of LLP- and RLP-forms of *htel3ΔT*/*P1* hetero-GQs

Notably, both kinetically favourable LLP-form and thermodynamically controlled RLP-form of ***htel3ΔT/P1*** exhibited similar melting points (Supplementary Figure S15). This was because these two pseudo-mirror GQs of ***htel3ΔT***/***P1*** shared many structural similarities in terms of numbers of H-bonds, H-bonding patterns, numbers of stacked G-tetrads and the *syn* and *anti* glycosidic angle patterns. In addition, both pseudo-mirror GQs had the same (3 + 1) hybrid strand orientation with two TTA edgewise loops that respectively span a wide groove and a narrow groove. All of these identitical structural features essentially made LLP- and RLP-GQs exhibit similar thermal stability.

It is unlikely that these hetero-GQ complexes remain static. Instead, the association/disassociation of a short G-rich probe within a hetero-GQ complex constantly occurs. The single G-rich oligonucleotide ***htel3ΔT*** itself has three G-tracts and presumably can involve an ensemble of G-triplex intermediates as proposed by many other laboratories (51–55). We assumed that the formation/disassembly of LLP- and RLP-GQs were involved in the corresponding intermediate states of both LLP- and RLP-G-triplexes as the most plausible candidates for on-pathway intermediates adopted by the same target sequence of ***htel3ΔT***. Intrinsically, the LLP G-triplex intermediate of ***htel3ΔT*** might be more readily to accept the incoming probe ***P1***, leading to a rapid formation of the kinetically favorable LLP-form of hetero-GQ ***htel3ΔT/P1***. In contrast, the RLP G-triplex of ***htel3ΔT*** had a relatively slower association rate with ***P1*** to re-assemble into the thermodynamically controlled RLP-form of hetero-GQ ***htel3ΔT/P1***.

### Flanking base alterations of short G-rich probe variants significantly affect associations with the same target of *htel3ΔT*

In addition to d(TAGGGT) of ***P1***, two other analogues of a short single-repeat fragment of human telomeric DNA, d(GGGTTA) and d(TTAGGG) (***P2*** and ***P3***, respectively; Table 1), were also titrated with the target of ***htel3ΔT*** (Supplementary Figure S7). ***P1*** associated with ***htel3ΔT*** to form two coexisting GQs in equilibrium either after slow annealing or after long-term untreated incubation at room temperature. In contrast, the association of ***htel3ΔT*** with either ***P2*** or ***P3*** yielded only a single hetero-GQ after annealing.

The structural identification of hetero-GQ complexes of ***htel3Δ******T***/***P2*** and ***htel3Δ******T***/***P3*** are detailed in the Supplementary text and Figures S8-S13. GQ topology is essentially dependent on the characteristic arrangement of G-tetrads which can be structurally identified based on inter-residue imino H1-aromatic H8 connections in the NOESY spectrum. In comparison, the same hydrogen-bond alignments and directionality within each G-tetrad (G1→G9→G13→G24, G2←G8←G14←G25, and G3←G7←G15←G26 in black) were shared with complex ***htel3Δ******T***/***P2*** (Supplementary Figures S10A and S10D) and the LLP-form of complex ***htel3ΔT***/***P1*** (Figure 5A and C); whereas the same pattern (G1←G9←G13←G24, G2→G8→G14→G25 and G3→G7→G15→G26 in red) were shared with complex ***htel3ΔT***/***P3*** (Supplementary Figures S13A and S13D) and the RLP-form of complex ***htel3ΔT***/***P1*** (Figure 5A and C). Accordingly, the GQ folding topologies of complex ***htel3ΔT/P2*** (Supplementary Figure S10E) and the LLP-form of complex ***htel3ΔT***/***P1*** (Figure 5C in black) were the same; while the GQ complex ***htel3ΔT***/***P3*** (Supplementary Figure S13E) adopted the same topology as that of RLP-form of ***htel3ΔT***/***P1*** (Figure 5C in red). The findings indicated that the flanking base alterations of short G-rich probe variants were also important in determining the loop progressions of hetero-GQs and in regulating the population ratio between the LLP- and RLP-forms (Supplementary Figure S14). There was a preference for LLP-form formation whenever at least one or several non-G flanking bases were present at the 3′-end of short G-rich probe variants. The formation of RLP-form hetero-GQ was preferred only in the presence of a bare 3′-terminus without any non-G flanking residue. Once formed, the hetero-GQs of ***htel3Δ******T***/***P1***, ***htel3ΔT***/***P2*** and ***htel3ΔT***/***P3*** were stable at physiological temperatures, as shown in UV melting experiments (Supplementary Figure S15).

### Structural refinement of *htel3ΔT*/*P3* hetero-GQ represents the RLP-form hetero-GQ

**Figure 6.**
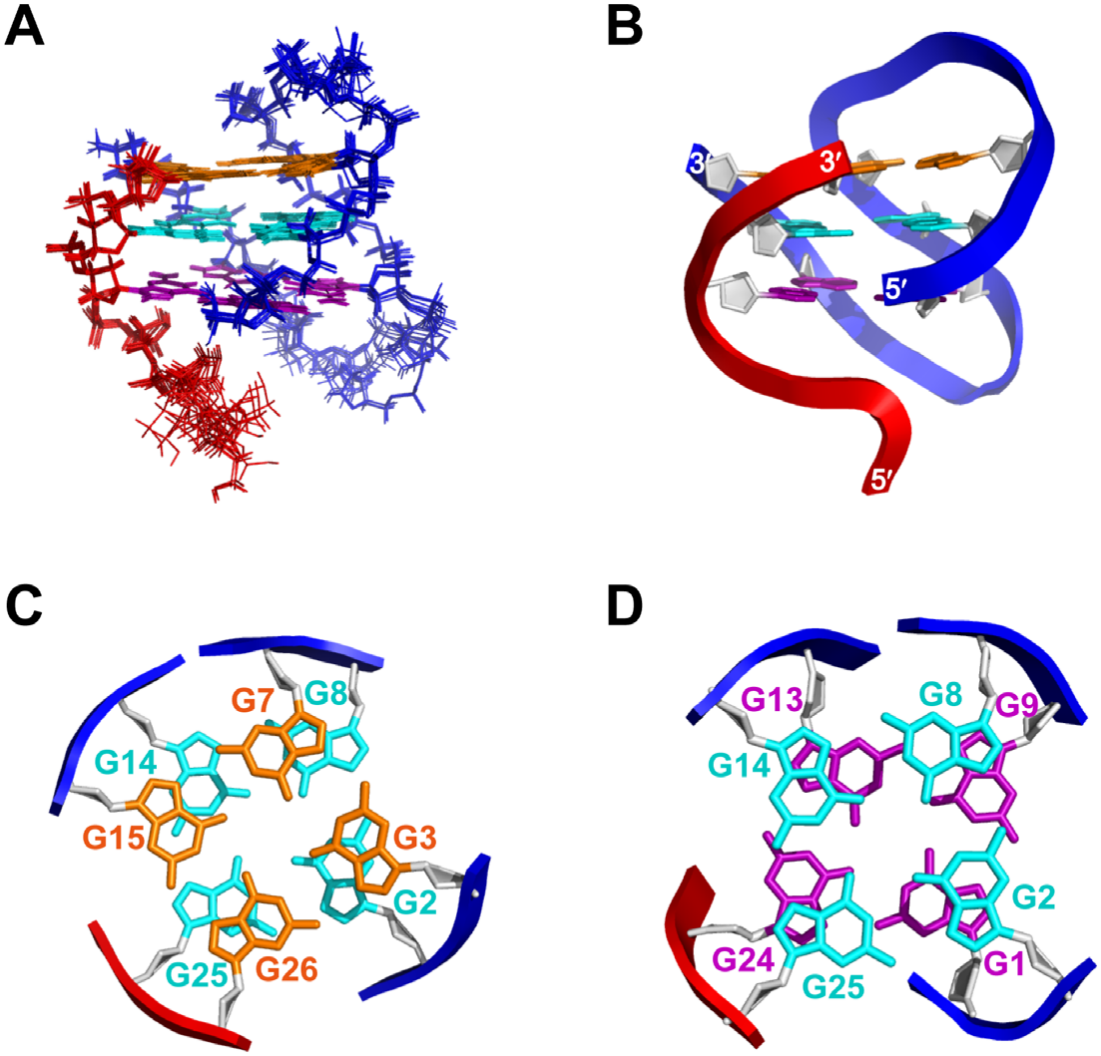
Solution structure of ***htel3ΔT***/***P3*** (similar to the RLP-form of ***htel3ΔT***/***P1***). (**A**) The 10 lowest energy structures are superimposed. Guanine bases in the top, middle, and bottom G-tetrads of a GQ core are indicated in orange, cyan, and purple, respectively. The backbones of ***htel3ΔT*** and ***P3***are coloured blue and red, respectively. (**B**) Cartoon representation of a representative refined structure. The backbones are displayed in a rectangular view coloured blue and red, respectively. (**C**) Stacking of G3·G7·G15·G26 (base in orange) over G2·G8·G14·G25 (base in cyan). (**D**) Stacking of G2·G8·G14·G25 (base in cyan) over G1·G24·G13·G9 (base in purple). The backbones of ***htel3ΔT*** and ***P3***are indicated by blue and red rectangles, and the sugar of guanines is coloured light grey. The residue numbers are the same as those shown in the schematic folding topology of ***htel3ΔT***/***P3*,** as illustrated in Supplementary Figure S13E.

Notably, the NMR spectra of the single structured ***htel3ΔT***/***P3*** GQ complex (Supplementary Figures S7, S11–S13) had better quality with less overlapping resonances. In contrast, the coexistence of both the LLP- and RLP-forms of ***htel3Δ******T***/***P1*** produced extensive overlap of some regions of the spectra (Figure 4). Since the ***htel3Δ******T***/***P3*** complex had a simplified NMR spectra and adopted the same folding topology as that of the RLP-form of ***htel3ΔT***/***P1***, the ***htel3Δ******T***/***P3*** complex (Figure 6 and Supplementary Figure S16) was chosen for further NMR structural refinements to represent the novel RLP hetero-GQ topology of ***htel3Δ******T***/***P1*** (Figure 5C). The complete chemical shift assignments of imino, amino, base, and sugar protons, as well as most ^13^C and ^15^N heavy atoms for the ***htel3Δ******T***/***P3*** complex are listed in Supplementary Table S1. The structure of ***htel3Δ******T***/***P3*** GQ was calculated based on NMR restraints by using the XPLOR-NIH program (version 3.0.3) (33–35). The restraint and structural statistical data are presented in Table 2. Since the LLP-form of complex ***htel3Δ******T***/***P1*** (Figure 5C) or the folding topology of ***htel3Δ******T***/***P2*** (Supplementary Figure S10E) was similar to that of the previously reported ***htel3***/***P1*** complex (Figure 1B) (14), no further structural refinements were carried out for these two LLP-forms.

**Table 2.** NMR restraints and structure statistics of ***htel3******ΔT**/**P3***

**A. NMR restraints**	
total number of DNA distance restraints	405
exchangeable distance restraints	72
nonexchangeable distance restraints	333
interresidue restraints	136
intraresidue restraints	169
hydrogen bond restraints	24
torsion angle restraints	21
**B. Structural statistics**	
NOE violations	
number > 0.2Å	0.8 ± 0.8
rms deviation of violations	0.002 ± 0.000
deviation from the ideal covalent geometry	
bond length (Å)	0.003 ± 0.000
bond angle (deg)	0.381 ± 0.007
impropers (deg)	0.223 ± 0.005
pairwise rms deviation (Å) (10 refined structures)	
G-tetrad core	0.26 ± 0.06
all heavy atoms	1.39 ± 0.38

### Solution structure of *htel3ΔT*/*P3* hetero-GQ with novel RLP

Ten superimposed lowest-energy intensity-refined structures of the ***htel3ΔT***/***P3*** GQs are displayed in Figure 6A. The G-tetrad core of ***htel3Δ******T***/***P3*** was converged with a root mean squared deviation (RMSD) of 0.26 ± 0.06 Å. The edgewise loops were more flexible than the G-tetrad core, and the entire global GQ structure of ***htel3Δ******T***/***P3*** had an RMSD of 1.39 ± 0.38 Å (Table 2). A representative refined structure is shown as a cartoon representation in Figure 6B, in which the hetero-GQ of ***htel3Δ******T***/***P3*** is a right-handed four-stranded helical assembly. The longer ***htel3ΔT*** strand (blue backbone) associated with the ***P3*** short strand (red backbone) to assemble a hetero-GQ with three different layers of G-tetrads (Figure 6B). The stacking patterns between these three G-tetrads, including G3→G7→G15→G26, G2→G8→G14→G25, and G1←G9←G13←G24, in which the H-bonding directionality is indicated by an arrow, from an H-bonding donor (arrow tail) to an H-bonding acceptor (arrow head), are shown in Figure 6C and D. One narrow, one wide, and two medium grooves were evident in the surface view (Supplementary Figure S16).

Two TTA looping segments of the blue target ***htel3ΔT*** strand functioned as edgewise loops in the RLPs (Figure 6B). The overhanging TTA segment of the red ***P3*** strand protruded from the G-tetrad core (Figure 6B). Each TTA edgewise loop of the blue target ***htel3ΔT*** linked two adjacent antiparallel G-columns. Notably, in other human telomeric DNA GQs, additional base pairs or triads formed by looping and overhanging TTA segments cap on the G-tetrad core (56,57). However, no capping of A⋅T base pairs was evident in the ***htel3Δ******T***/***P3*** hetero-GQ.

### Thioflavin T preferentially binds with RLP-form over coexisting LLP-form of *htel3ΔT*/*P1* hetero-GQs

Telomeric DNA GQs are attractive targets amenable to recognition or stabilization by small molecules (11,58). In this study, the structural dissimilarities between the LLP- and RLP-form GQs of ***htel3ΔT***/***P1*** were less significant. We explored whether a ligand could preferentially bind to these two pseudo-mirror counter partners of the LLP- and RLP-forms. Here, we found that ThT, a popular fluorescence dye and GQ ligand (26,59), preferentially recognized the RLP-form at low ThT:GQ molar ratios.

**Figure 7.**
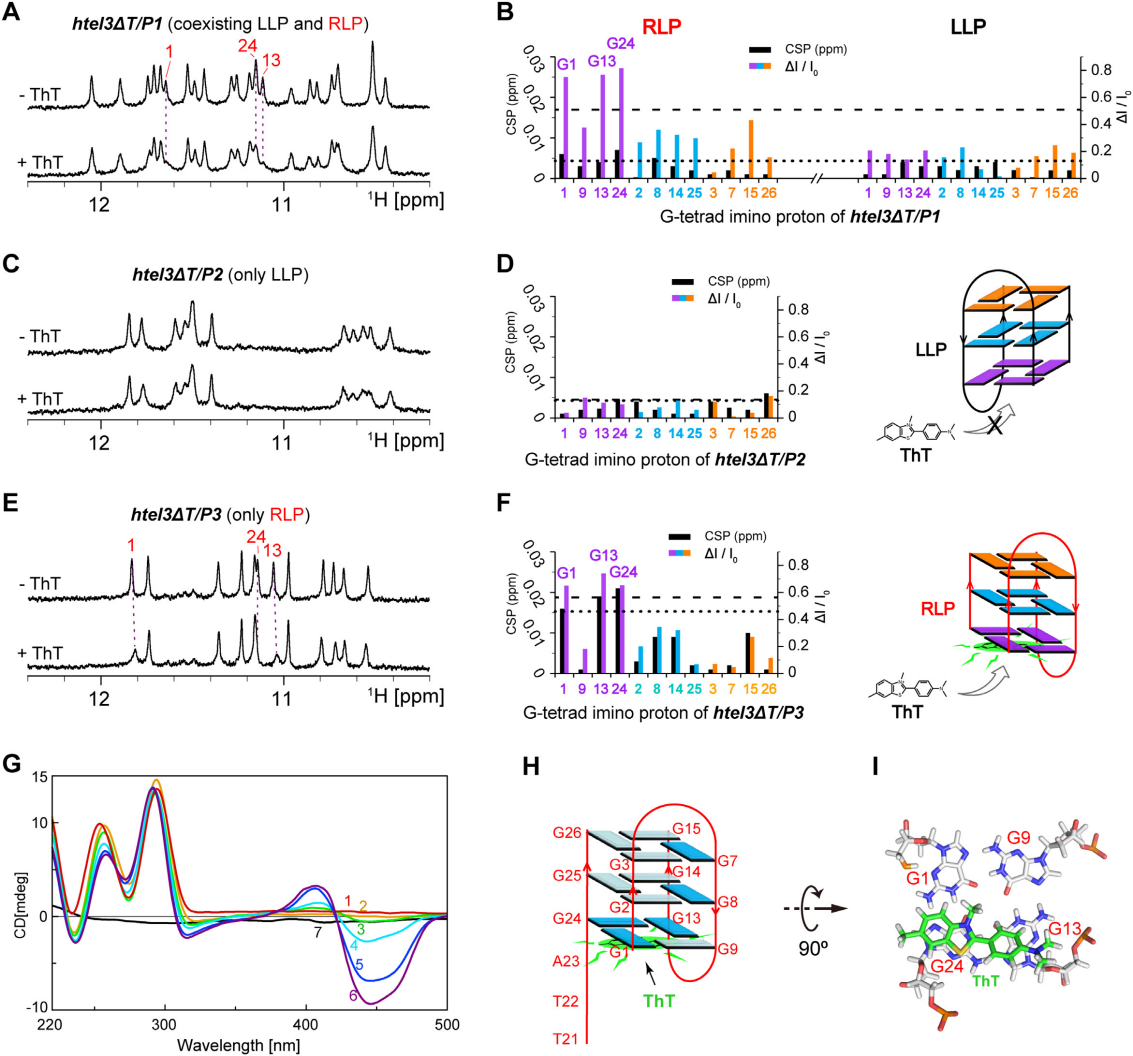
Interactions between hetero-GQs and titrated thioflavin T (ThT). The expanded imino proton region of one-dimensional ^1^H NMR spectra of 0.4 mM ***htel3ΔT***/***P1*** (**A**), 0.2 mM ***htel3ΔT***/***P2*** (**C**) and 0.2 mM ***htel3ΔT***/***P3*** (**E**) were titrated with 0.025 equivalent ThT. The guanine imino proton peaks with the most obvious perturbations that belong to the RLP-form of ***htel3Δ******T***/***P1*** are highlighted with purple dotted lines and numbered in red. The residue numbers are the same as those shown in the schematic folding topologies of two coexisting LLP-form and RLP-form of ***htel3Δ******T***/***P1*** (Figure 5C). Plot of chemical shift perturbation (CSP on left vertical axis) and signal intensity difference ratio (ΔI/I_0_ on right vertical axis) comparing G-tetrad imino protons (as horizontal axis) alone and with 0.025 equivalent ThT for tested hetero-GQs of ***htel3ΔT***/***P1*** (**B**), ***htel3ΔT***/***P2*** (**D**) and ***htel3ΔT***/***P3*** (**F**). *I*_0_ is the intensity of free GQs and Δ*I* is the intensity difference of GQs without and with ThT titration. The dotted lines and dashed lines indicate one standard deviation above the average value of CSP and Δ*I*/*I*_0_, respectively. For clarity, the top, middle, and bottom G-tetrads are coloured orange, cyan, and purple, respectively. (**G**) Induced CD spectra recorded for 0.04 mM ***htel3ΔT***/***P3*** in 20 mM Na-Pi 100 mM NaCl buffer with ThT titration at concentrations of: (1) 0 mM, (2) 0.05 mM, (3) 0.1 mM, (4) 0.25 mM, (5) 0.4 mM, (6) 0.6 mM. (7) 0.6 mM ThT alone in 20 mM Na-Pi, 100 mM NaCl buffer as a blank control. (**H**) Schematic folding topology of ***htel3Δ******T***/***P3*** binding to ThT. ThT is coloured green. *Syn-* and *anti*-guanines of ***htel3Δ******T***/***P3*** are indicated by dark blue and light blue rectangles, respectively. (**I**) Stacking of ThT (coloured green) preferentially over the bottom (G1·G24·G13·G9) G-tetrad of RLP-form of ***htel3Δ******T***/***P3***, based on molecular dynamics simulation.

In an equimolar mixture of ***htel3ΔT*** and ***P1*,** both the LLP- and RLP-forms of ***htel3ΔT***/***P1*** GQs coexisted in equilibrium. The entire course of ThT stepwise titration into this GQ mixture which contained two coexisting LLP-form and RLP-form was monitored through the imino proton region (Supplementary Figure S17) and the aromatic proton region (Supplementary Figure S18) of one-dimensional ^1^H NMR spectra, until a high loading of 1.2 equivalent of ThT (relative to the total amount of both the LLP- and RLP-GQs) was reached. During titration, we found that the preferential binding of ThT became more evident at a low ThT:GQ target ratio of 0.025:1 (Figure 7A), by comparison of the imino proton region of one-dimensional ^1^H NMR spectra in the presence and absence of ThT. The observation of only one set of imino proton signals of GQs, which gradually changed in chemical shifts upon addition of a sub-stoichiometric amount of ThT (Figure 7A, Supplementary Figure S17), indicated that NMR titration spectra are in the fast exchange limit on the chemical shift timescale due to weaker binding between ThT and GQ target. However, the selectivity of ThT was still sufficient to differentiate these two coexisting GQs with opposite loop progressions of ***htel3ΔT***/***P1***. The involvement of particular GQ residues in the ThT binding event was revealed by comparing their chemical shift values in the free and complexed states (Figure 7B). Only G1, G13, and G24 residues of the bottom G-tetrad of the RLP-form of ***htel3ΔT***/***P1*** (as topologically illustrated in Figure 5C) exhibited the largest perturbations of imino proton signals that were either shifted to new positions or largely broadened upon ThT titration, at a low ThT:GQ target ratio of 0.025:1 (Figure 7A and B). In contrast, other guanine imino proton signals, including those of the LLP-form of ***htel3ΔT***/***P1***, exhibited minimal or no perturbations (Figure 7A and B). The NMR mapping data suggest that ThT preferentially interacts with the RLP-form of ***htel3ΔT***/***P1***, rather than the coexisting LLP-form of ***htel3ΔT***/***P1***. ThT stacks over the bottom G-tetrad plane of this RLP-form.

NMR signals often broaden or even disappear during ligand titration (60). ThT inherently has a molecular rotor behavior which generates a variety of conformational ensembles of ThT (61). In NMR, the rotation of ThT aromatic ring system altered local magnetic field and consequently this rotational conformation ensembles of bound ThT would cause the chemical shift fluctuations of proximal G-tetrad signals. Free ThT alone exhibited sharp NMR signals (Supplementary Figure S28) due to the fast conformational exchange of ring rotation on chemical shift timescale. Once bound with GQ target, the rotating motion of the ThT ring system was restricted (view the attached file of ‘MD_RLP-form + ThT.mp4’ in the Supplementary data). This slowed ring rotating of bound ThT made the conformational fluctuations into an intermediate exchange on chemical shift timescale, thus it caused NMR signal broadenings preferentially for the GQ residues immediately proximal to the bound ThT.

### ThT preferentially binds with RLP-GQ of *htel3ΔT*/*P3* over LLP-GQ of *htel3ΔT*/*P2*

Unlike ***htel3ΔT***/***P1*,** which adopts two coexisting RLP- and LLP-forms, the hetero-GQ of ***htel3ΔT***/***P3*** exclusively adopts the RLP-form. The GQ of ***htel3Δ******T***/***P2*** only adopts the LLP-form. To further validate the preferential binding of ThT, NMR spectra of chemical shift perturbation upon ThT titration were acquired in details for the GQs of ***htel3Δ******T***/***P3*** (Supplementary Figures S22 and S23) and ***htel3ΔT***/***P2*** (Supplementary Figure S26). Once again, at a low ThT:target GQ molar ratio, the NMR titrations revealed that ThT still preferentially recognized the bottom G-tetrad (G1⋅G24⋅G13⋅G9) of the RLP-form of ***htel3Δ******T***/***P3*** (Figure 7E and F; Supplementary Figures S22A, S22E and S22F); whereas the LLP-form of ***htel3Δ******T***/***P2*** had no evident interaction with ThT (Figure 7C and D). As a note, the selective binding of G24 rather than overlapped G25 upon ThT titration (Figure 7E) and the cross-over movement of ^1^H chemical shift pertubations for G24 (Supplementary Figure S22A) were further confirmed by ^15^N-filtered spectra using singly guanine-labelled samples of G24 (Supplementary Figure S22E) and G25 (Supplementary Figure S22F). Usually, the observation of intermolecular NOEs between bound ThT and GQ target will greatly improve the structural constraints of ThT binding site. Unfortunately, the aromatic proton signals of ThT became seriously broaden upon binding with target GQ of ***htel3**Δ**T***/***P3*** (Supplementary Figure S28). As a result, it was impossible to detect intermolecular NOEs between the broaden-out singals of bound ThT and a GQ target.

### ICD supported the end stacking mode of ThT without altering the global topology of target GQ

ThT interacts with GQs and duplexes either by embedding in a groove or stacking on a G-tetrad (26). Induced CD experiments revealed a negative intensity enhancement at 445 nm upon the addition of ThT to ***htel3Δ******T***/***P3*** GQ, either with a high ThT loading of 1.2 equivalent (Figure 7G) or alternatively at a low 0.05 sub-stoichiometric equivalent of ThT (Supplementary Figure S30B). These observations confirmed that ThT interacts with the RLP-form GQ of ***htel3Δ******T***/***P3*** via the end stacking pattern on a G-tetrad, rather than embedding in a groove (Figure 7G and Supplementary Figure S30B). In addition, the shape of the twin shoulder peaks at 260 nm and 295 nm (Figure 7G), indicative of the characteristic (3 + 1) hybrid GQ topology, remained essentially unchanged during ThT titrations. Thus, the preservation of the (3 + 1) hybrid topology for this ***htel3Δ******T***/***P3*** GQ was inferred when it bound to the ligand ThT. As a note, in order to examine the ThT binding mode only when ThT was in low equivalent condition, much excessive amounts of GQ target had to be added into ThT:GQ mixture (Supplementary Figure S30). Although the Chirascan circular dichroism spectrometer usually can handle much more concentrated DNA samples at sub mM (25), a signal overflow at 260∼300 nm specifically for excessive GQs was still unavoid, whereas the collections of ThT signals at 445 nm however remained undamaged (Supplementary Figure S30). The negative signal enhancement at 445 nm specifically for ThT without a signal overflow was observed preferentially for RLP-GQ of ***htel3ΔT***/***P3*** (Supplementary Figure S30B) rather than that of LLP-GQ of ***htel3ΔT***/***P2*** (Supplementary Figure S30A).

### Fluorescence enhancement data supported the preferential recognition of RLP-form GQ by ThT

ThT has two main aromatic moieties: benzothiazole (BZT) and dimethylamino-benzene (DMAB) (Supplementary Figure S19A) (62). For unbound ThT, the free rotation of BZT and DMAB around the C-C single bond quenches ThT emission fluorescence (Supplementary Figure S19A) (61,63). However, when end stacked with a G-tetrad, ThT rotation is restricted and ThT planarity is enforced, leading to enhanced ThT fluorescence (26,64,65). Our fluorescence enhancement data additionally supported the preferential recognition by ThT of the RLP-form GQ of ***htel3ΔT***/***P3*** over LLP-form GQ of ***htel3ΔT***/***P2*** (Supplementary Figure S20). Fluorescence enhancement was the most obvious when RLP-form GQ of ***htel3Δ******T***/***P3*** interacted with titrated ThT.

### Molecular dynamics (MD) simulation and NMR studies on ThT binding site at target RLP-GQ of *htel3ΔT*/*P3*

A 150 ns MD simulation was performed using Amber 18 software (44). This complex system between ThT and GQ ***htel3ΔT***/***P3*** was in a relatively stable state after 90 ns, as assessed from the changing trend of heavy atom RMSD over the course of the simulation (Supplementary Figure S21). The obtained lowest-energy complex structure in which ThT bound to the target GQ of ***htel3ΔT***/***P3*** is shown in Figure 7I and Supplementary Figure S19B. To assess the changes in ThT planarity, the bound form of ThT was extracted from the MD simulation complex structure, whereas the free form of ThT was energy-minimized using Schrodinger software (41) (Supplementary Figure S19A). By comparing the overlapped structures of ThT between the free form (purple) and bound form (green), the entire aromatic ring system of ThT (BZT ring and DMAB ring) became more coplanar upon preferential binding with RLP-form GQ of ***htel3ΔT***/***P3*** (Supplementary Figure S19A), consistent with the fluorescence enhancement results (Supplementary Figure S20).

ThT has a relatively smaller aromatic moiety than a given G-tetrad plane (Figure 7I). According to the MD simulation, the stacking of ThT was centred more closely on top of the G13⋅G24 Hoogsteen base pair region within the whole (G1⋅G24⋅G13⋅G9) G-tetrad plane of ***htel3ΔT/P3*** GQ (Figure 7H, I, and Supplementary Figure S19B). The imino proton of G1 was in close proximity to the edge of the ThT aromatic moiety, whereas the base H8 proton of G1 remained far away from ThT (Figure 7I). Both the imino and base H8 protons of G9 were parallel and distant from ThT ring moieties (Figure 7I). More MD and NMR details of this ThT binding pocket at the bottom (G1⋅G24⋅G13⋅G9) G-tetrad of RLP-GQ of ***htel3ΔT***/***P3*** are described in the supplementary text and Figures S22-S23.

### ThT preferentially binds with RLP hetero-GQs compared with several representative intramolecular GQs or telomeric duplex context

GQs are mostly formed by intramolecular folding of a single guanine-rich oligonucleotide sequence. Less frequently they are formed by the intermolecular association of multiple strands. In general, unimolecular GQs in telomeres and the promoter regions of oncogenes are prevalent and have been regarded as targets of great interest because of their roles in crucial biological processes, such as aging and cancer. To further assess the specificity of ThT, several representative intramolecular GQs were examined by NMR titrations (Supplementary Table S3). These intramolecular GQs, which folded mostly by telomeric DNA, have parallel, antiparallel, or (3 + 1) hybrid topologies, and are structurally diverse (Supplementary Table S3). Essentially, at the same low ratio between ThT and target GQ, no obvious changes in guanine imino proton signals in one-dimensional ^1^H spectra were observed upon ThT titration. These observations confirmed the preferential binding of ThT with RLP hetero-GQ of ***htel3ΔT***/***P1*** or ***htel3ΔT***/***P3*** over intramolecular GQs.

Except for a single-stranded overhang, there is also a duplex region in the telomere which may interact with ThT as well. We prepared a mixture that simultaneously contained the corresponding telomeric duplex context (annealed between equivalent ***htel3ΔT*** and complementary ***htel3ΔT-C***, Table 1) and both the RLP-form and LLP-form of hetero-GQs (complex of ***htel3ΔT***/***P1***, Table 1). In one-dimensional ^1^H spectra, the characteristic imino proton signals were readily distinguishable between the duplex at 13–14 ppm and GQs at 11–12 ppm. Within a small ThT:target ratio, only an apparent broadening of imino proton peaks for RLP-form was detected, whereas other representative signals belonging to the LLP-form and duplex context remained unchanged (Supplementary Figure S24). In addition, the results of fluorescence enhancement experiments supported the preferential recognition of ThT with RLP-form GQ of ***htel3Δ******T***/***P3*** over either the LLP-form GQ of ***htel3Δ******T*****/*P2*** or the corresponding duplex context (Supplementary Figure S20), in which the most obvious fluorescence enhancement was detected when RLP-form GQ of ***htel3Δ******T***/***P3*** interacted with titrated ThT. The duplex context exhibited the least fluorescence enhancement.

### Non-selective interactions between ThT and DNA targets at higher molar ratios

At higher molar ratios between ThT and DNA target (1.2:1 or higher), not surprisingly, a severely broadened NMR spectra of all examined DNA samples, including hetero-GQs, intra-GQs, and DNA duplex context, exhibited non-selective interactions with ligand ThT (Supplementary Figure S25). Except for the expected π-π interactions, the positively charged N-atom present in ThT also enabled the contribution of electrostatic interactions, most likely in a non-selective way, with negatively charged phosphates that are extremely rich in DNA, as found for other GQ-binders (66–68). In addition, over-titrated ThT may have more potential to occupy additional binding sites elsewhere on other terminal G-tetrad surfaces. Therefore, all these non-selective interactions would reduce the selectivity of ThT, especially once at a high ThT loading. Thus its binding constant measurement became unreliable and less meaningful in the presence of non-selective interactions.

ThT has an inherently complex fluorescence signal quenching due to susceptible self-aggregation at a higher loading concentration (69). Usually, avoiding a high loading of fluorescence dyes has been a primary concern in research. Despite considerable efforts, a GQ-modulating drug is not yet available, and molecules that discriminate between different GQ structures have proven challenging to develop (70). We found that the preferential binding of ThT became even more evident at low ThT loading (in the presence of 20 excessive equivalents of DNA). ThT enabled preferential recognition with RLP-GQ over either LLP-GQ or the corresponding telomeric DNA duplex context, and even over classic intra-GQs. These attractive features make ThT an interesting lead compound with future implications for the development of more selective nucleic acid-binding ligands with biological activity.

### Effects of other naturally occurring variations of flanking bases composition at the extreme 3′-end of telomeric DNA G-overhang during formation of hetero-GQs

The single-stranded G-overhang of human telomeric DNA is composed of tandem repeats of the repetitive 6-nt unit, 5′-GGGTTA-3′. Systematically, there are six different endings at the 3′ terminus, each with a specific permutation of the 5′-GGGTTA-3′ repeat (Supplementary Figure S27A, Table 1). Interestingly, the individual association of all these target sequence variants containing at least one or several flanking bases at the 3′-terminus with the same probe ***P1*** yielded a solo LLP-form of well-folded hetero-GQ (Supplementary Figure S27C–F). As experimental support for the adoption of the LLP-form, NMR (Supplementary Figure S27D–F) and CD (Supplementary Figure S27H and S27I) spectra of ***htel3M1***/***P1***, ***htel3M2***/***P1***, and ***htel3M3***/***P1*** (Table 1) were quite comparable to that of ***htel3***/***P1*** (Supplementary Figure S27C and S27H), an LLP-form hetero-GQ with a (3 + 1) hybrid topology (14). Additionally, no evident NMR spectrum perturbations were observed upon a low equivalent ThT titration, suggesting that ***htel3M1***/***P1***, ***htel3M2***/***P1***, and ***htel3M3***/***P1*** (Supplementary Figure S27C–E) also adopted a similar LLP folding topology as ***htel3***/***P1*** (Supplementary Figure S27B). On the other hand, the exclusive formation of RLP-form hetero-GQ with a (3 + 1) hybrid topology was preferred only for complex ***htel3Δ******T***/***P3***, in which the end with a bare 3′-terminus without any non-G flanking residue was required for either target sequence ***htel3ΔT*** or short probe ***P3*** (Supplementary Figure S13). As an exception, the extra G2-tract at the 3′-end made ***htel3M4*** (Table 1) readily distinguishable from the other sequence variants. This ***htel3M4*** sequence contains four G-tracts, and thus likely self-folds into an intramolecular GQ of two G-tetrads, as indicated by the observation of eight sharp imino proton peaks of guanines in the one-dimensional ^1^H spectrum (Supplementary Figure S27G-I). The stability of the ***htel3M4*** intramolecular GQ was sufficient to prevent the binding of ***P1*** (Supplementary Figure S27G-II).

### Comparison between hetero-dimeric GQs of *htel3ΔT*/*P1* and unimolecular *C9orf72* GQs of four d(G4C2) repeats

A comparable phenomenon that a single oligonucleotide strand enabled formation of two structurally similar unimolecular GQs but with opposite loop progressions was previously reported (71,72). A prolonged expansion of d(G4C2) repeats within non-coding region of *C9orf72* gene has been identified as the most common genetic cause of two devastating neurodegenerative disorders, amyotrophic lateral sclerosis (ALS) and frontotemporal dementia (FTD) (73,74). Formation of DNA G-quadruplexes within expanded d(G4C2) repeats was proposed to drive ALS and FTD pathogenesis. Plevc reported that a single oligonucleotide of d(G4C2)_3_G4, a truncated four-repeat fragment of *C9orf72*, enabled formation of two major structurally similar unimolecular GQs in K^+^ solution (71,72). Both *C9orf72* unimolecular GQs adopted the same antiparallel topology with four stacked G-tetrads and three edgewise CC loops, the same G-residue arrays to construct each individual G-tetrad, and the same *syn* and *anti* glycosidic angle patterns of guanine residues along the sequence and in the G-tetrads, but in completely opposite loop progressions.

Two folding conditions, solution pH and the annealing/cooling rate, controlled the relative populations of these two unimolecular *C9orf72* GQs with opposite loop progressions. The kinetically favourable RLP-GQ (named **AQU** (71,72)) was favored by rapid cooling rate and was stabilized under slightly acidic conditions (pH 5.8); whereas the thermodynamically favourable LLP-GQ (named **NAN** (71,72)) was preferred by slow annealing process under neutral conditions (pH 7.2). In contrast, the LLP-form of ***htel3ΔT/P1*** is kinetically favourable while the RLP-form of ***htel3ΔT/P1*** is thermodynamically controlled.

As both unimolecular *C9orf72* GQs with four stacked G-tetrad layers exhibited high melting points (above 75°C), the interconversions between RLP kinetic structure and LLP thermodynamic structure of these unimolecular GQs were unable to spontaneously perform at room temperature, an indicative of large kinetic barriers for structural interconversion. In comparison, the LLP- and RLP-forms of hetero-dimeric GQ complex ***htel3******ΔT***/***P1*** had three stacked G-tetrads and exhibited relatively low melting points of ∼49°C. Given this lower kinetic barrier, a spontaneous structural intercoversion between the LLP- and RLP-forms of ***htel3ΔT***/***P1*** was able to achieve at room temperature under neutral conditions (pH 6.8). Moreover, unlike TTA edgewise loops in hetero-dimeric GQs of ***htel3ΔT***/***P1***, the pH-susceptible nature of cytosine residues fine-tuned interactions of edgewise C-C loops in *C9orf72* GQs of four d(G4C2) repeats, the protonated cytosine residue not only provided an extra contribution to GQ stabilization thus rendered a high kinetic barrier of structural interconversion, but also determined the population ratio between LLP and RLP-forms of *C9orf72* unimolecular GQs (71,72).

## CONCLUSION

The last residue at the extreme 3′-end of telomeric DNA G-strand is heterogeneous in base composition, without a precise ending. In this study, we explore how alteration of the last 3′-terminal base significantly affects the mutual recognition between two different G-rich oligomers, the three-repeat and single-repeat fragments of human telomeric DNA, in the formation of hetero-GQs.

Associations between target ***htel3ΔT*** of d(GGGTTAGGGTTAGGG) and probe ***P1*** of d(TAGGGT) in Na^+^ solution yield two coexisting forms of (3 + 1) hybrid hetero-GQs with completely opposite loop progressions: the kinetically favourable LLP-form and the thermodynamically controlled RLP-form. However, only the adoption of a single LLP-form has been previously reported between the same probe ***P1*** and a target variant ***htel3*** of d(GGGTTAGGGTTAGGG***T***) having one extra 3′-end thymine.

These two hetero-GQs of the LLP- and RLP-forms of complex ***htel3ΔT/P1*** might at first seem to be pseudo-mirror counter partners with a particular emphasis on the global folding topology, and they exhibit less dramatically distinct structural dissimilarities. Interestingly, even in a mixture of both LLP- and RLP-forms coexisting in equilibrium, the fluorescence dye ThT when in much less molar equivalent, preferentially recognized the RLP-form over the LLP-form of hetero-GQs. To a greater extent, ThT when in much less molar equivalent, also preferentially bound to RLP hetero-GQ than with the corresponding telomeric DNA duplex context or several other representative intramolecular GQs.

Additionally, the flanking base alterations of short G-rich probes dramatically affected formation of these hetero-GQs. There was a preference for LLP-form formation whenever at least one or several non-G flanking bases were present at the 3′-end of short G-rich probes. The formation of RLP-form hetero-GQ was preferred only in the presence of a bare 3′-terminus without any non-G flanking residue. Our findings are expected to have important implications for the structure of telomeric DNA and targeting GQs in telomeres.

Furthermore, other GQ structures have recently been used as catalysts for enantioselective Friedel-Crafts (75) and Diels-Alder reactions (76,77). Our finding of RLP-GQ and LLP-GQ as pseudo-mirror counter partners opens more possibilities in this research area, by providing a new experimental platform to improve stereo control and to expand substrate variety.

## DATA AVAILABILITY

The coordinates of 10 structures of the ***htel3Δ******T***/***P3*** have been deposited in the Protein Data Bank (accession code 7DO1). The chemical shifts have been deposited in the BioMagResBank under accession code 50625.

## Supplementary Material

gkab755_Supplemental_Files
