# Supplementary material for: Two coexisting pseudo-mirror heteromolecular telomeric G-quadruplexes in opposite loop progressions differentially recognized by a low equivalent of Thioflavin T
Source: Nucleic Acids Res. 2021 Sep 9;49(18):10717–34. doi: 10.1093/nar/gkab755 (PMC8501994; doi:10.1093/nar/gkab755)
Supplement: gkab755_Supplemental_Files [file gkab755_supplemental_files.zip › caption of MD_RLP-form ThT_NaZ.docx]

Caption: 150 ns molecular dynamics simulation of the end-stacking mode of ThT over the outer G-tetrad (G1·G24·G13·G9) of RLP-GQ ***htel3ΔT/P3***. The vertical and front view are shown in vedio.The sugar-phosphate backbones of ***htel3ΔT*** and ***P3*** are coloured blue and red, respectively. Bases and suagers are coloured green. Ligand ThT is coloured orange.
