# Supplementary material for: Two coexisting pseudo-mirror heteromolecular telomeric G-quadruplexes in opposite loop progressions differentially recognized by a low equivalent of Thioflavin T
Source: Nucleic Acids Res. 2021 Sep 9;49(18):10717–34. doi: 10.1093/nar/gkab755 (PMC8501994; doi:10.1093/nar/gkab755)
Supplement: gkab755_Supplemental_Files [file gkab755_supplemental_files.zip › Revised_Supplementary.pdf]

Table of Contents

Supplementary Text

- ♦ Folding and unfolding kinetics of two GQ forms of the ***htel3ΔT/P1*** complex.
- ♦ CD and NMR hydrogen-deuterium exchange experiments supported the folding topologies for both LLP-form and RLP-form of ***htel3ΔT/P1*** hetero-GQs.
- ♦ NMR structural identification of ***htel3ΔT/P2*** and ***htel3ΔT/P3*** hetero-GQs.
- ♦ NMR evidence to support MD simulation of the ThT binding pocket at the target RLP-GQ of ***htel3ΔT/P3***.

Supplementary Figures

Supplementary Tables

References

Supplementary Text

Folding and unfolding kinetics of two GQ forms of *htel3ΔT*/*P1* complex

Unlike the fast formation of the LLP-form denoted by the asterisk, another RLP-form of the ***htel3ΔT/P1*** GQ complex denoted by the circle has a slower formation. Upon the addition of ***P1*** to ***htel3ΔT*** at a molar ratio of 1:1, the formation of the LLP- and RLP-forms was monitored in detail at different time points by the acquisition of one-dimensional ^{1}H spectra (Figure S1). The initial formation rates of the LLP- and RLP-form of ***htel3ΔT/P1*** complex were calculated from the slopes of the initial linear portion (within the first 25 min) as 3.36 and 0.43 nM/min, respectively(1,2). The formation of the LLP-form was markedly faster than that of RLP-form.

In the presence of complementary DNA, transiently unfolded GQ structures can be trapped as a more stable duplex, driving the complete unfolding of GQs. As duplex formation is fast(3), the observed slow apparent kinetics of duplex formation and concomitant GQ disappearance are assumed to have arisen from the rate-limiting unfolding of the GQ structure(4). The unfolding kinetics of the LLP- and RLP-forms from a mixture of both forms in an equilibrium mixture of equimolar ***htel3ΔT*** and ***P1*** (Figure S2A) were tracked by one-dimensional ^{1}H NMR after addition of a 5-fold excess of complementary DNA oligomer of d(CCCTAACCCTAACCC) (termed ***htel3ΔT-C*** as listed in Table 1), based on the well-established complementary strand trap method(5-7). NMR spectra were collected periodically over a period of 3.5 h during unfolding induced by the addition of complementary strands (Figure S2). Analysis of the differential disappearance of these well-resolved imino proton signals representative of the LLP-form and RLP-form, respectively, revealed that the LLP-form unfolds more readily than the RLP-form. Overall, the kinetics of folding and unfolding were both faster for the LLP-form, whereas both were slower for the RLP-form.

CD and NMR hydrogen-deuterium exchange experiments supported the folding topologies for both LLP-form and RLP-form of *htel3ΔT/P1* hetero-GQs

CD spectra are sensitive to G-tetrad stacking and have distinctive shapes for different corresponding GQ topologies(8), reflecting the various combinations of *syn* or *anti* glycosidic bond angles (GBA) between the consecutively stacked guanosines along a G-tract(9). The positive band at 295 nm was indicative of the stacking of adjacent guanosines with different GBA. The same GBA exhibited a positive band at 260 nm. The LLP-form or RLP-form of ***htel3ΔT*/*P1*** adopted (3+1) hybrid strand orientations with equal amounts of the same GBAs and opposite GBAs (Figure 3C). The observation of two positive bands at 260 and 295 nm in the CD spectra of ***htel3ΔT*/*P1*** (Figure S5) was consistent with the (3+1) folding topologies shown in Figure 3C.

Data of subsequent hydrogen-deuterium exchange experiments supported these folding topologies. The guanines from the central G-tetrad (G2, G8, G14, and G25) were among the most protected imino protons. They exchanged with D_2O relatively slowly in both LLP-form and RLP-form (Figure S6A).

NMR structural identification of *htel3ΔT/P2* and *htel3ΔT/P3* hetero-GQs

The procedures for the assignment of ***htel3ΔT/P2*** and ***htel3ΔT/P*3** were basically the same as those for ***htel3ΔT/P1*** described above. Since the association of ***htel3ΔT*** with either ***P2*** or ***P3*** yielded only a single hetero-GQ, the assignments of ***htel3ΔT/P2*** and ***htel3ΔT/P*3** were easier. The non-ambiguous resonance assignments of guanine imino and H8 protons were first performed using the sequence-specific labelling approach (Figures S8 and S11) with samples of 4% or 100% ^{13}C, ^{15}N-enrichment (Table 1). Next, the non-exchangeable base H8/H6 and sugar H1′ proton assignments were determined by tracing the sequential NOE connectivities, for either non-isotopically labelled d(G1G2G3T4T5A6G7G8G9T10T11A12G13G14G15) and d(G24G25G26T27T28A29) of ***htel3ΔT/P2***, or d(G1G2G3T4T5A6G7G8G9T10T11A12G13G14G15) and d(T21T22A23G24G25G26) of ***htel3ΔT/P3***, respectively, in the NOESY spectra of 250 ms mixing time recorded in D_2O solution of Na-Pi buffer (pH 6.8) (Figures S9A and S12A). Finally, the guanine imino proton assignments for ***htel3ΔT/P2*** and ***htel3ΔT/P3*** were achieved in the ^{1}H-^{13}C HMBC experiment (Figures S9B and S12B) based on the correlation between guanine base H8 and imino H1 protons through $^{13}C5$ at natural abundance. Overall, these assignments using non-labelled samples are consistent with those assigned non-ambiguously using labelled samples.

In the stacked NOESY spectra of ***htel3ΔT/P2*** and ***htel3ΔT/P3*** with a 50 ms short mixing time, five strong H8-H1′ cross peaks were observed for the G1, G7, G8, G13, and G24 residues, indicating their adoption of *syn* glycosidic conformations (Figures S10B and S13B). Subsequently, two positive bands at 260 and 295 nm were observed in the CD spectra of ***htel3ΔT/P2*** and ***htel3ΔT/P3*** (Figure S5), indicating that they all adopted (3+1) folding topologies. Furthermore, the hydrogen-bond alignments and directionality within each G-tetrad of ***htel3ΔT/P2*** and ***htel3ΔT/P3*** were determined based on the establishment of imino-H8 connections in the NOESY spectrum of 250 ms mixing time in H_2O: G1→G9→G13→G24, G2←G8←G14←G25 and G3←G7←G15←G26 for ***htel3ΔT/P2*** (Figure S10A) and G1←G9←G13←G24, G2→G8→G14→G25 and G3→G7→G15→G26 for ***htel3ΔT/P3*** (Figure S13A). In the figures, the H-bonding directionality is indicated by an arrow, from donor (arrow tail) to an acceptor (arrow head). Accordingly, the folding topologies of ***htel3ΔT/P2*** (Figure S10E) and ***htel3ΔT/P3*** (Figure S13E) were established. Both adopted a (3+1) hybrid strand orientation with three G-tetrad layers and two edgewise loops, in opposite loop progressions.

NMR evidence to support MD simulation of the ThT binding pocket at the target RLP-GQ of htel3ΔT/P3

Even within the same G-tetrad plane of (G1·G24·G13·G9) for ***htel3ΔT/P3*** GQ, NMR chemical shift perturbations (CSPs) of each individual guanine still behaved differently. The imino protons of G1, G13, and G24 presented the largest perturbations that were essentially broadened upon ThT titration. In contrast, the imino protons of G1, as well as other guanines of the central G-tetrad and another terminal G-tetrad, remained almost unchanged (Figures 7E and S22). Similarly, the base H8 proton of G13 showed evident CSPs, while no apparent changes were observed for the base H8 proton of G9

(Figure S23). These NMR observations were consistent with the MD simulation. The overlap of the base H8 protons of G24 and G1 was too extensive to reliably detect a perturbation.

Previous studies demonstrated that variations in groove dimensions, loops, and flanking segments are allowed for binding affinity and specificity, despite GQ-binding ligands stacked on a common plane of G-tetrad(10-13). As the experimental support for our MD simulation, the following NMR experimental data illustrate specific contacts responsible for preferential binding of ThT with RLP-form over LLP-form.

First, the MD simulation revealed good stacking interaction of the looped A12 residue immediately below the G9 corner of the (G1·G24·G13·G9) tetrad. To accommodate this partial occupancy of looped A12, bound ThT is driven closer to the G13·G24 base pair region of the bottom (G1·G24·G13·G9) tetrad. This plausible driving process is shown in a more vivid manner with dynamic motions (see the attached file of 'MD_RLP-form + ThT.mp4' in the Supplementary data). Eventually, the H2 proton of A12 is in the immediate vicinity of ThT and points towards the ring edge of ThT, whereas the H8 proton of A12 is located far away (Figures S19B and S23). These spatial arrangements are supported by NMR data, in which the characteristic base H2 proton signal of A12 is clearly broadened upon titration with ThT, whereas the base H8 proton signal of A12 remains unchanged, or at least much less evident (Figure S23).

Furthermore, MD simulation revealed that bound ThT is loosely warped around by other looped and overhung residues T11, T21, T22, and A23, which form a binding pocket to accommodate ThT, accounting for the origin of binding specificity. The corresponding NMR data are shown in Figures S19 and S23. Collectively, ThT preferentially recognises the specific shape moiety of GQ, rather than a particular sequence. The bottom G-tetrad of the RLP-form, rather than that of the LLP-form, is the preferential binding site for ThT.

As described above, the spatial arrangements for loop residues of T11, and especially A12, are critical in the favourable contribution to ThT binding, preferentially at the bottom G-tetrad of the RLP-form. Although the size of the terminal G-tetrad surface is the same in both RLP and LLP GQs, their flanking loops have opposite loop progressions that lead to the buildup of distinct groove dimensions. Regarding the binding site of ThT at the bottom G-tetrad of (G1·G24·G13·G9), the flanking T10-T11-A12 loop spans a narrow groove in RLP-form. Conversely, the same T10-T11-A12 loop spans a wide groove in LLP-form (Figures 5C). This distinct difference in groove dimensions between the RLP-form and LLP-form will influence the spatial arrangements of these bridging loop residues of T10, T11, and A12. Hence there could be preferential binding of ThT with RLP-form over LLP-form.

Supplementary Figures

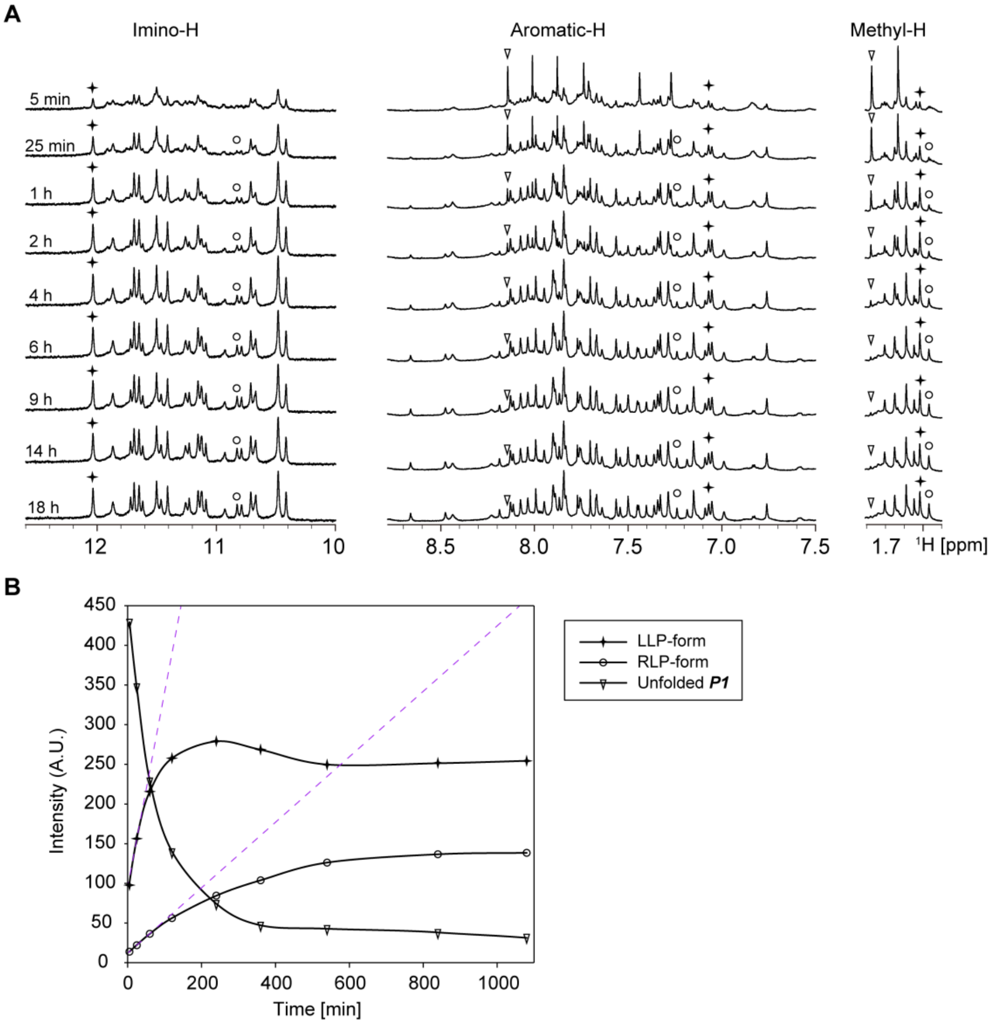

Figure S1. Formation of LLP-form and RLP-form of ***htel3ΔT/P1*** in real time after mixing ***htel3ΔT*** and equimolar ***P1***. **(A)** One-dimensional ^{1}H spectra of ***htel3ΔT/P1*** collected at different times. The representative signals of the LLP-form, RLP-form, and unfolded ***P1*** are marked by asterisks, circles, and triangles, respectively. **(B)** Population evolution (in arbitrary units, A.U.) of the LLP-form, RLP-form, and unfolded ***P1*** in an equimolar mixture of ***htel3ΔT/P1*** as a function of time is presented as a linear graph. The initial formation rates of the LLP- and RLP-form of ***htel3ΔT/P1*** complex were calculated through the slopes of the initial linear portion within the first 25 min (purple dashed lines) as 3.36 and 0.43 nM/min, respectively(1,2). All spectra were collected at 288K, 0.4 mM DNA, 20 mM Na-Pi buffer (pH 6.8), and 100 mM NaCl.

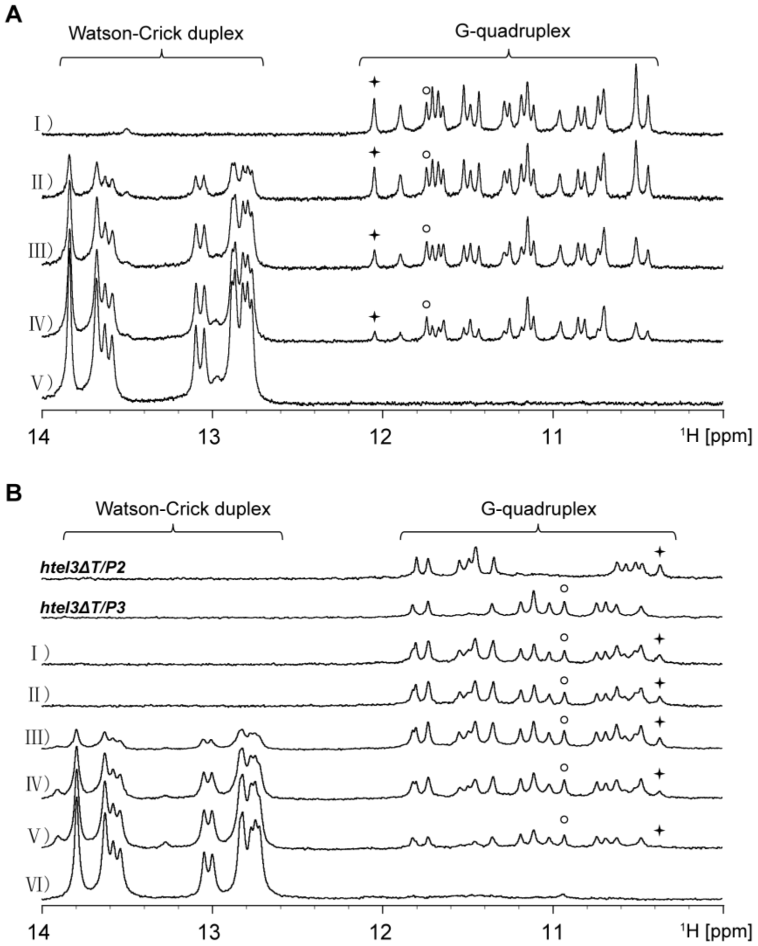

Figure S2. Stability comparison between the LLP-form and RLP-form by using the complementary strand trap method. **(A)** One-dimensional ^{1}H spectra of an equimolar mixture of ***htel3ΔT/P1*** (0.2 mM DNA strand concentration for each oligomer) after annealing and titration with complementary strand ***htel3ΔT-C*** in different amounts: I) 0 equivalent; II) 0.5 equivalent; III) 2 equivalent; IV) 4 equivalent; and V) 4 equivalent, followed by incubation at 288 K for 3 h. The representative signals for the LLP- and RLP-form of ***htel3ΔT/P1*** are denoted by asterisks and circles, respectively. **(B)** ***htel3ΔT/P2*** (0.2 mM) and ***htel3ΔT/P3*** were annealed independently and then mixed at equal concentrations before titration with complementary strand ***htel3ΔT-C*** in different amounts: I) 0 equivalent; II) 0 equivalent and incubated at 288 K for 21 h; III) 0.5 equivalent; IV) 1 equivalent; V) 2 equivalent; and VI) 2 equivalent, followed by incubation at 288 K for 3 h. The representative signals for ***htel3ΔT/P2*** and ***htel3ΔT/P3*** are denoted by asterisks and circles, respectively.

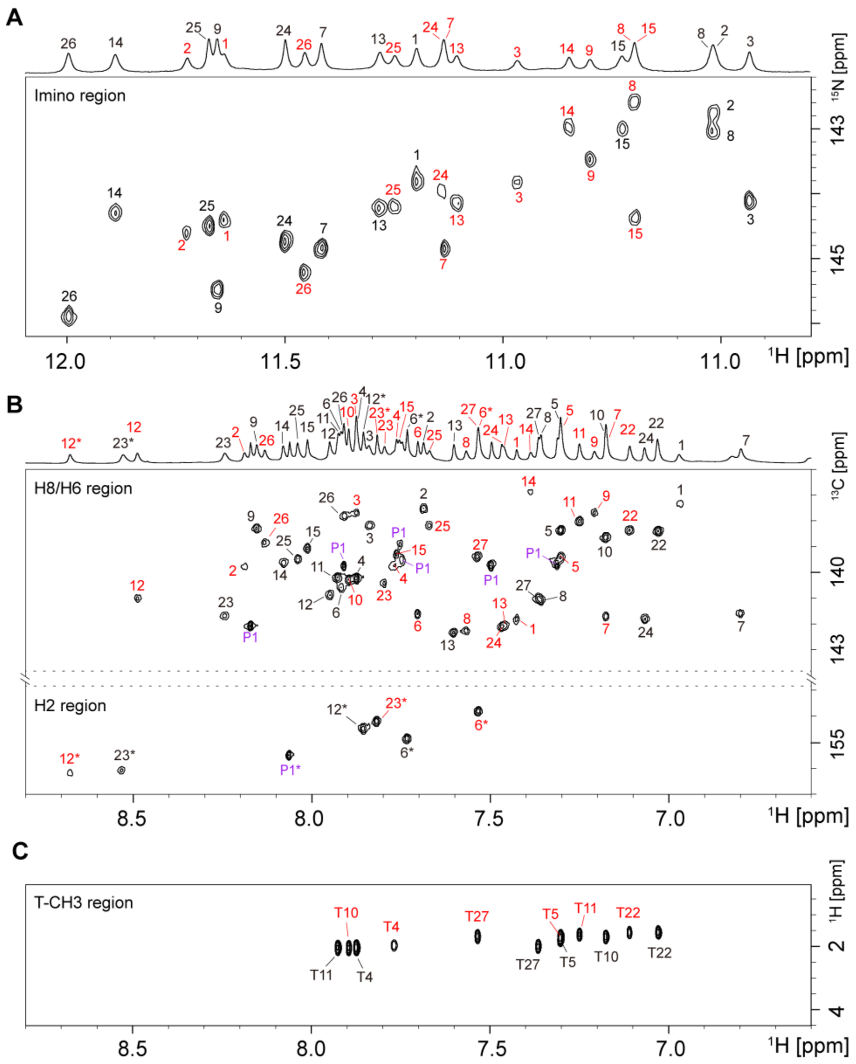

Figure S3. Two-dimensional through-bond NMR experiments of non-isotopically labelled complex ***htel3ΔT/P1***. **(A)** The expanded imino proton region of the ^{1}H-^{15}N HSQC spectrum. **(B)** The expanded base H8/H6/H2 proton region of the ^{1}H-^{13}C HSQC spectrum. **(C)** The expanded base H6 proton of thymine region (horizontal axis 7.0–8.0 ppm) and methyl CH_3 proton of thymine region (vertical axis 1.5–2.5 ppm) of the ^{1}H-^{1}H TOCSY spectrum. One-dimensional projections of imino and aromatic base protons are shown on the top. The H2 protons of adenine are marked by right superscript *. The residue numbers of the LLP-form and RLP-form of ***htel3ΔT***/***P1*** are coloured black and red, respectively. Cross peaks of unstructured portion of ***P1*** are coloured purple. Experimental conditions were 4 mM DNA, 20 mM Na-Pi buffer (pH 6.8), 100 mM NaCl, and 303 K.

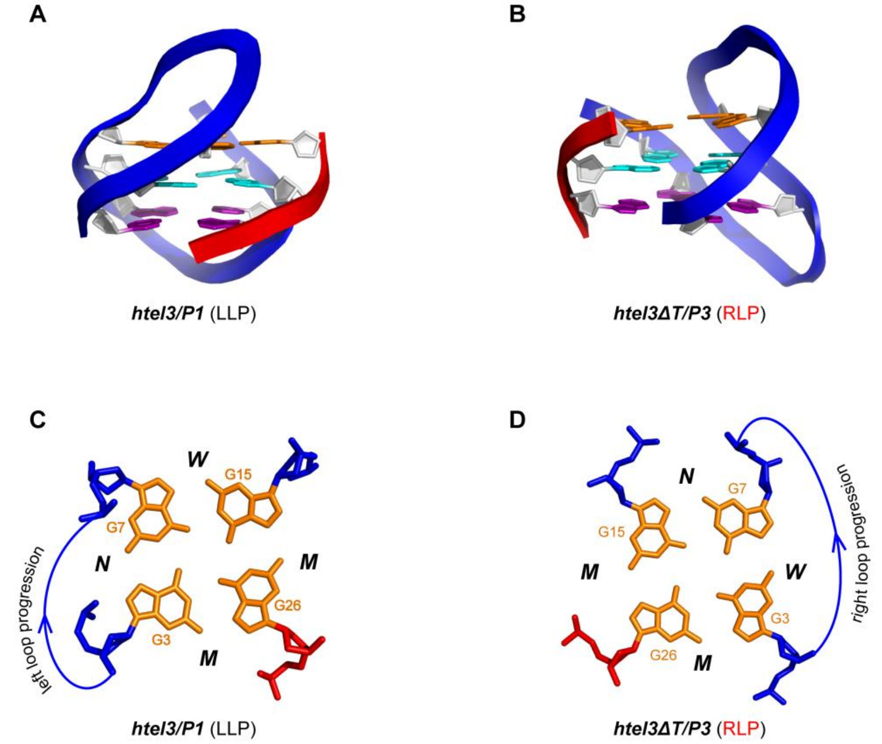

Thro

Figure S4. Comparison of solution structures of ***htel3***/***P1*** (similar to the LLP-form of ***htel3ΔT***/***P1***) and ***htel3ΔT***/***P3*** (similar to the RLP-form of ht***el3ΔT***/***P1***). Cartoon representation of representative refined structures of ***htel3***/***P1*** **(A)** and ***htel3ΔT***/***P3*** **(B)**. The backbones are displayed in a rectangle view coloured blue and red, respectively. G-tetrad (G3·G7·G15·G26) of ***htel3***/***P1*** **(C)** and G-tetrad (G3·G26·G15·G7) of ***htel3ΔT***/***P3*** **(D)** coloured orange. *W*, *M*, and *N* represent wide, medium, and narrow groove width, respectively. Directions of the loop progression of ***htel3*** and ***htel3ΔT*** are indicated by blue arrows.

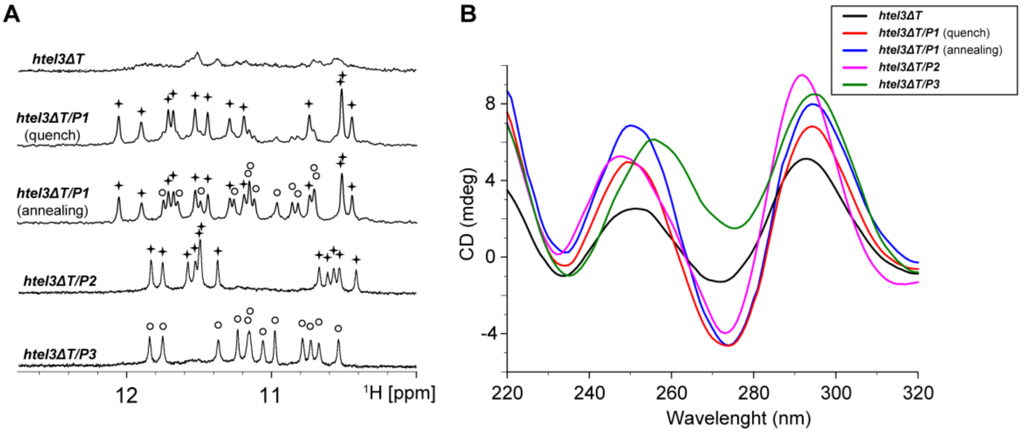

Figure S5. **(A)** Expanded imino proton region of one-dimensional ^{1}H spectra of ***htel3*** alone, ***htel3ΔT***/***P1*** (quench), ***htel3ΔT***/***P1*** (annealing), ***htel3ΔT***/***P2***, and ***htel3ΔT***/***P3***. The LLP-form and RLP-form of these hetero-GQs are marked by asterisks and circles, respectively. Experimental conditions: 0.2 mM DNA, 20 mM Na-Pi buffer (pH 6.8), 100 mM NaCl, and 288 K. **(B)** CD spectra of ***htel3*** alone, ***htel3ΔT***/***P1*** (quench), ***htel3ΔT***/***P1*** (annealing), ***htel3ΔT***/***P2***, and ***htel3ΔT***/***P3*** are coloured black, red, blue, magenta, and green, respectively. Experimental conditions: 40 µM DNA, 20 mM Na-Pi buffer (pH 6.8), 100 mM NaCl, and 288 K.

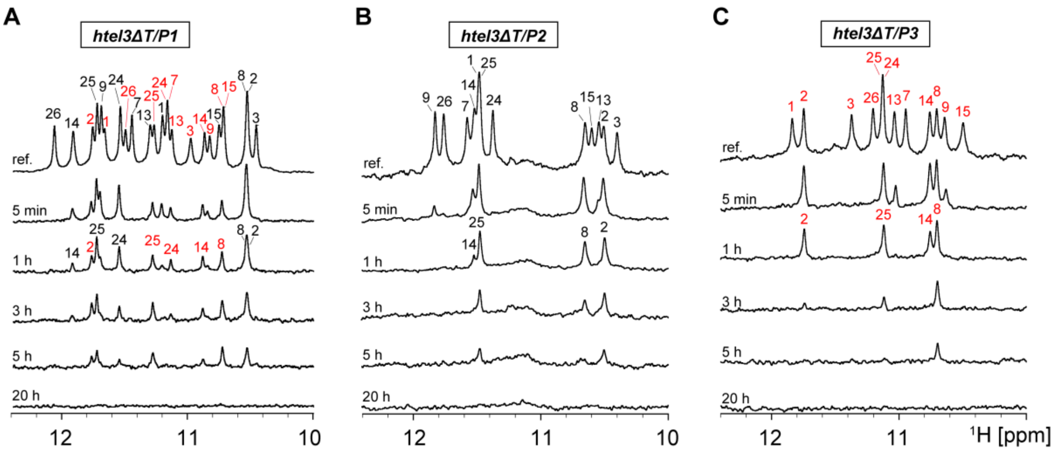

Figure S6. Imino proton spectra of ***htel3ΔT/P1*** **(A)**, ***htel3ΔT/P2*** **(B)**, and ***htel3ΔT/P3*** **(C)** in H_2O as reference and following hydrogen-deuterium exchange (HDX) in D_2O at different times from 5 min to 20 h. LLP-form and RLP-form of GQs are coloured black and red, respectively. Experimental conditions: 0.2 mM DNA, 20 mM Na-Pi buffer (pH 6.8), 100 mM NaCl, and 288 K.

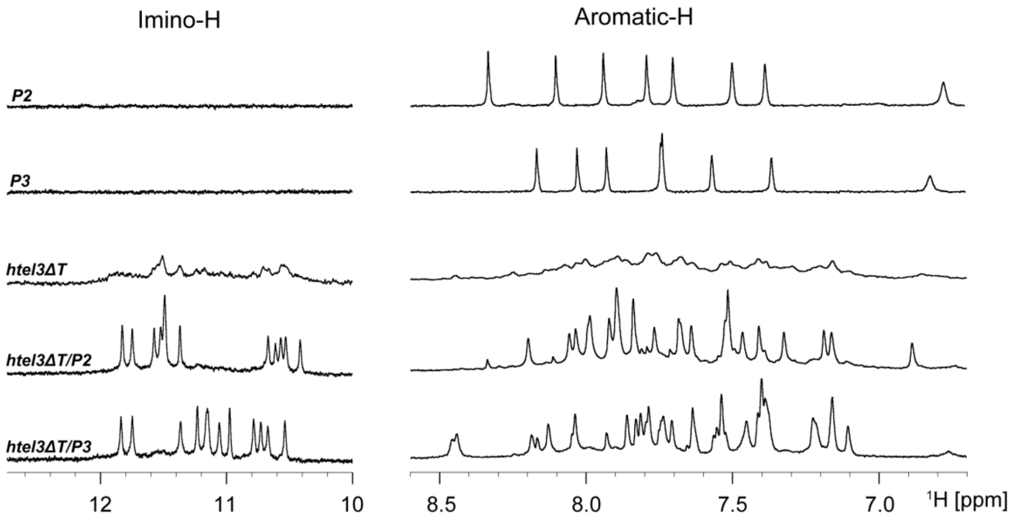

Figure S7. One-dimensional ^{1}H NMR spectra of ***P2*** alone, ***P3*** alone, ***htel3ΔT*** alone, complex of ***htel3ΔT/P2***, and complex of ***htel3ΔT/P3***. All samples were dissolved in 20 mM Na-Pi buffer (pH 6.8), 100 mM NaCl, 10% D_2O solution at 303 K. Strand concentration of each oligomer is 0.2 mM.

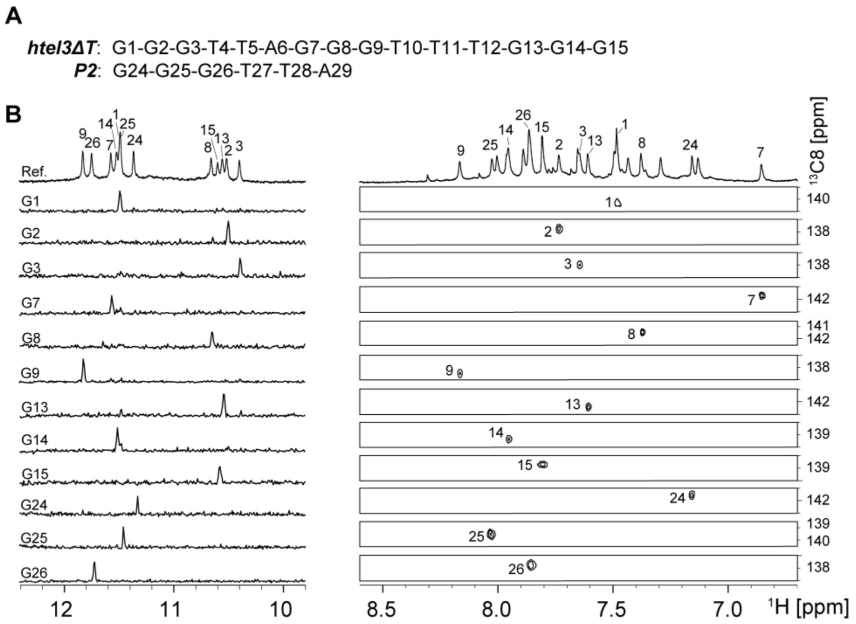

Figure S8. **(A)** The residue numbers of ***htel3ΔT/P2*** are shown on top. **(B)** ^{15}N-filtered spectra of imino protons (left panel) and ^{13}C-filtered spectra of aromatic base protons (right panel) of singly guanine-labelled ***htel3ΔT/P2*** with non-ambiguous resonance assignments indicated over the reference spectrum. Singly guanine-labelled samples were 4% ^{15}N, ^{13}C-labelled at positions G1, G2, G3, G7, G8, G9, G13, and G14, while 100% ^{15}N, ^{13}C-labelled at positions G15, G24, G25, and G26. The residue numbers of ***htel3ΔT/P2*** are coloured black to represent the adoption of LLP-form GQ. All samples were dissolved in 20 mM Na-Pi buffer (pH 6.8), 100 mM NaCl, and 10% D_2O solution at 303 K. Strand concentration of samples G1-G14 and samples G15-G26 are 0.6 mM and 0.1 mM, respectively.

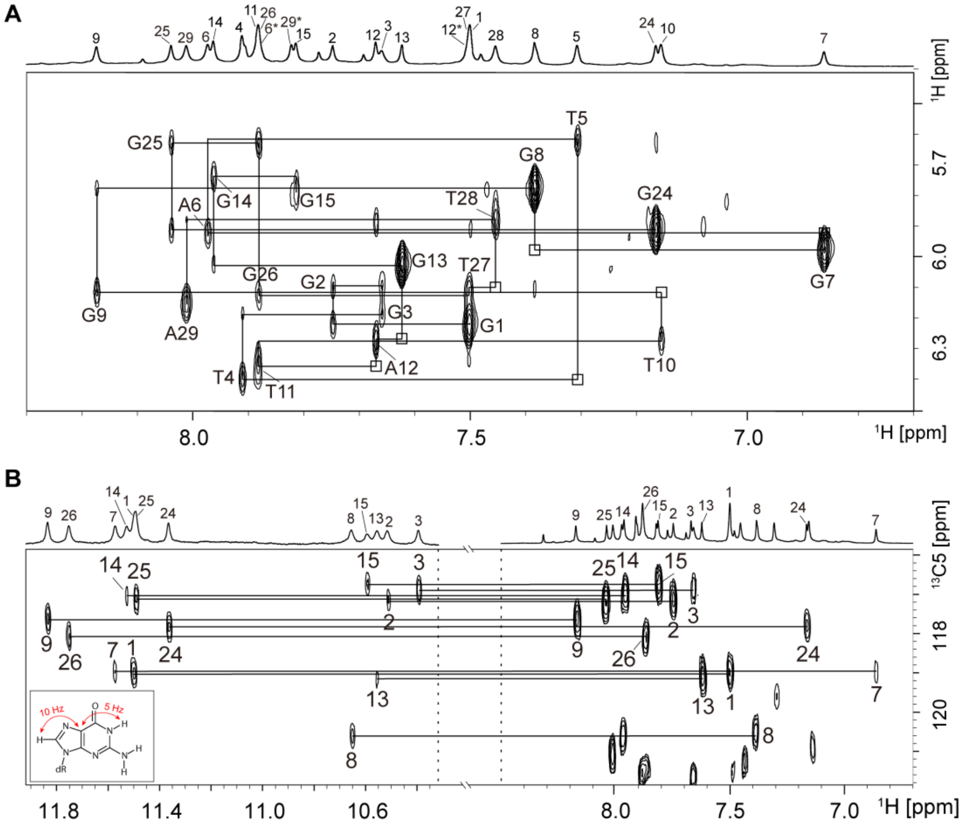

Figure S9. ^{1}H-^{1}H NOESY and ^{1}H-^{13}C HMBC spectra of ***htel3ΔT*/*P2*.** **(A)** Sequential walking in the NOESY spectrum (250 ms mixing time, D_2O) demonstrates the H8/H6-H1′ connectivity through-space. Cross peaks are labelled with residue numbers. The H2 protons of adenine are marked by right superscript *. Weak or missing sequential connectivities are labelled with rectangles. **(B)** Through-bond correlations between guanine imino and H8 protons via $^{13}C5$ at natural abundance by using long-range J-couplings shown in the inset. Experimental conditions: 3 mM DNA, 20 mM Na-Pi buffer (pH 6.8), 100 mM NaCl, and 303 K.

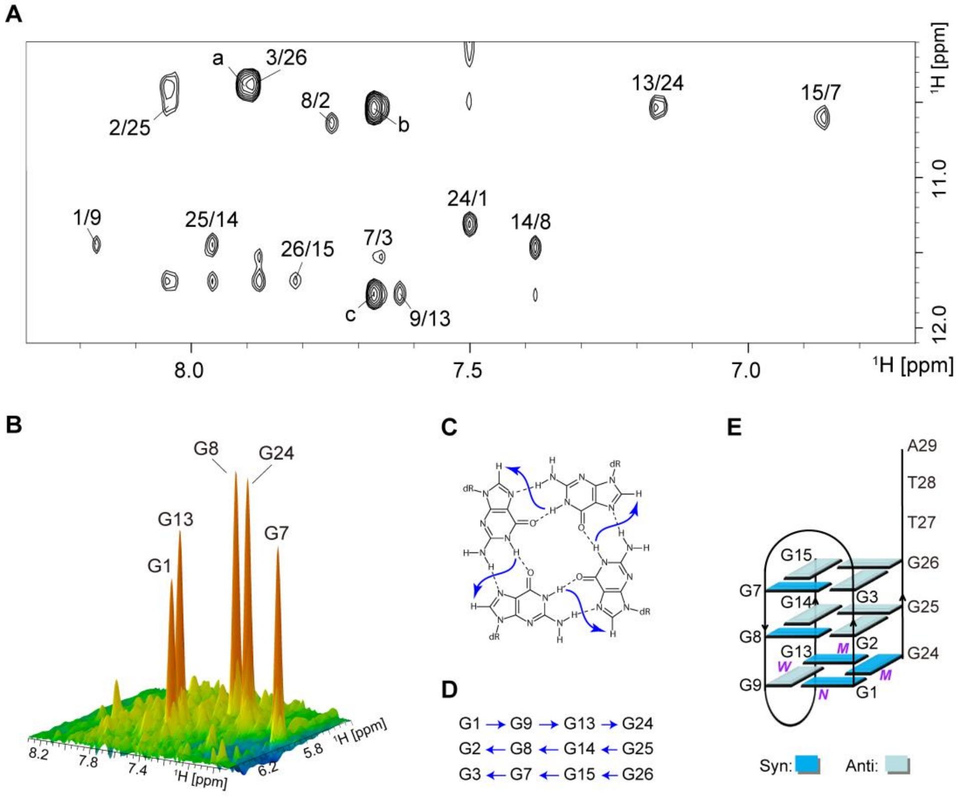

Figure S10. Folding topology of ***htel3ΔT*/*P2*** determined by NMR. **(A)** NOESY spectrum (250 ms mixing time, 10% D_2O/90% H_2O) shows inter-residue imino H1-base H8 cross peaks to identify the arrangements of G-tetrads. The guanine imino H1-base H8 cross peaks are labelled with residue numbers of imino H1 and base H8 protons in the first and second positions, respectively. The remaining strong cross peaks in the NOE spectrum are also identified as follows: a, G3H1/T4H6; b, G13H1/A12H8; c, G9H1/A12H8. **(B)** Stacked plot of two-dimensional NOESY spectrum (50 ms mixing time, D_2O) of ***htel3ΔT*/*P2***, distinctively shows the strong intra-residue H8-H1′ cross peaks for the *syn* guanines. **(C)** G-tetrad arrangement with characteristic imino-H8 NOE pattern indicated with arrows. **(D)** Three G-tetrads of ***htel3ΔT*/*P2*** are shown. The hydrogen-bond directionality within the same G-tetrad is indicated by blue arrows, from a donor (arrow tail) to an acceptor (arrow head). **(E)** Schematic folding topology of ***htel3ΔT*/*P2*** is shown. The backbones are shown as black solid lines to represent the adoption of LLP-form GQ. *Syn* and *anti* guanines are indicated by dark blue and light blue rectangles, respectively. Experimental conditions: 3 mM DNA, 20 mM Na-Pi buffer (pH 6.8), 100 mM NaCl, and 303 K.

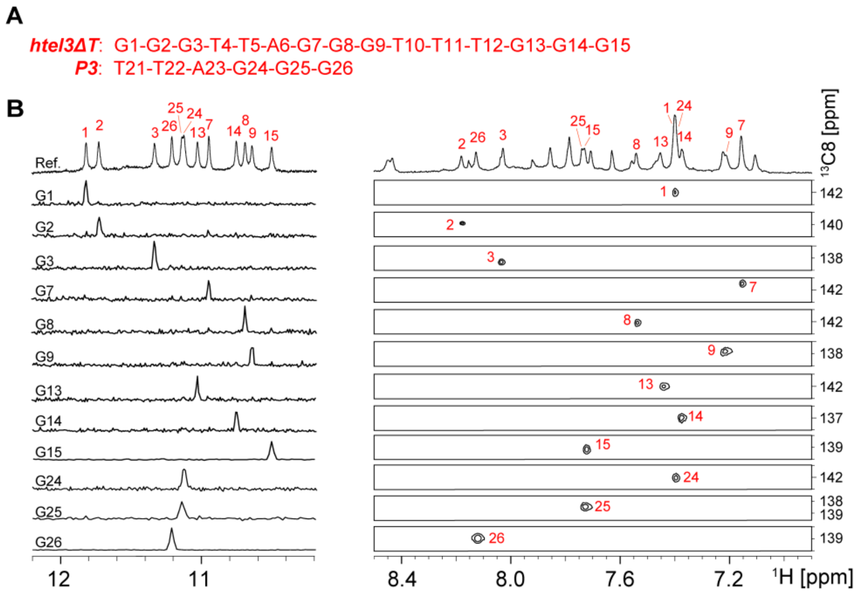

Figure S11. **(A)** The residue numbers of ***htel3ΔT*/*P3*** are shown on top. **(B)** ^{15}N-filtered spectra of imino protons (left panel) and ^{13}C-filtered spectra of aromatic base protons (right panel) of singly guanine-labelled ***htel3ΔT*/*P3*** with non-ambiguous resonance assignments indicated over the reference spectrum. Singly guanine-labelled samples were 4% ^{15}N, ^{13}C-labelled at positions G1, G2, G3, G7, G8, G9, G13 and G14, while 100% ^{15}N, ^{13}C-labelled at positions G15, G24, G25, G26. The residue numbers of ***htel3ΔT*/*P3*** are coloured red to represent the adoption of RLP-form GQ. All samples were dissolved in 20 mM Na-Pi buffer (pH 6.8), 100 mM NaCl, and 10% D_2O solution at 303 K. Strand concentration of samples G1-G14 and samples G15-G26 are 0.6 mM, and 0.1 mM, respectively.

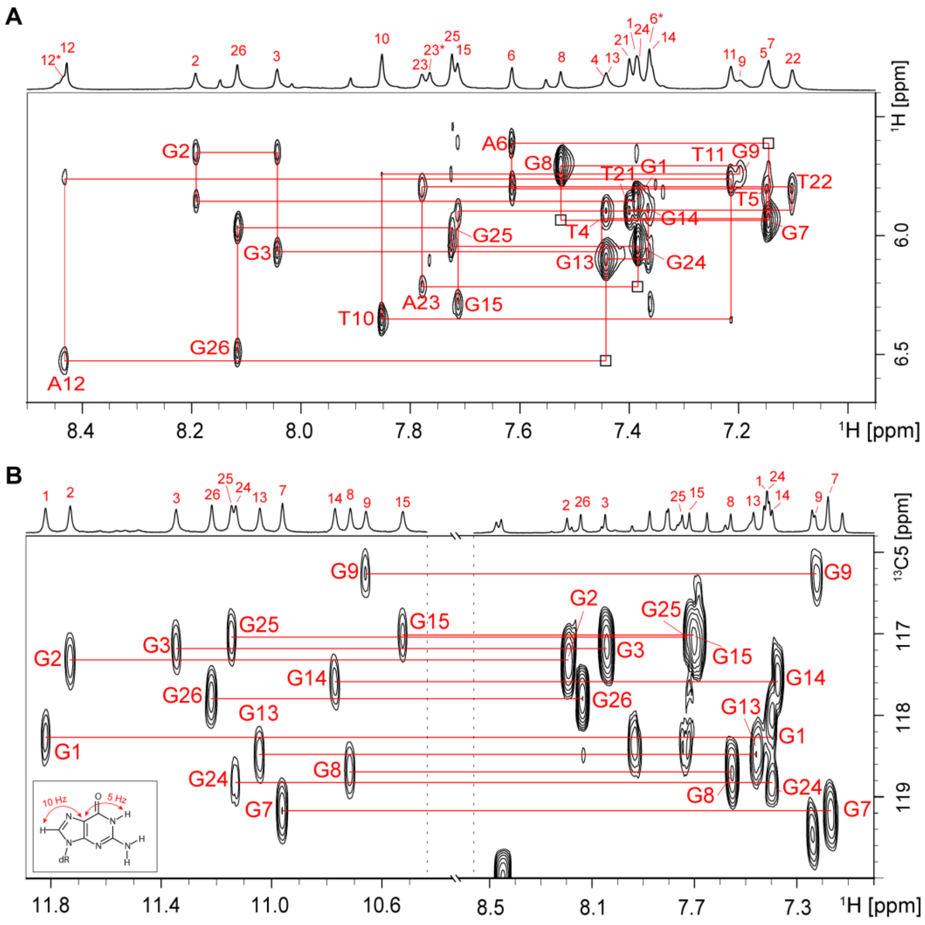

Figure S12. ^{1}H-^{1}H NOESY and ^{1}H-^{13}C HMBC spectra of ***htel3ΔT/P3.*** **(A)** Sequential walking in the NOESY spectrum (250 ms mixing time, D_2O) demonstrates the H8/H6-H1′ connectivity via space. Cross peaks are labelled with residue numbers. The H2 protons of adenine are marked by right superscript *. Weak or missing sequential connectivities are labelled with rectangles. **(B)** Through-bond correlations between guanine imino and H8 protons via ^{13}C5 at natural abundance by using long-range J-couplings shown in the inset. Experimental conditions: 3 mM DNA, 20 mM Na-Pi buffer (pH 6.8), 100 mM NaCl, and 303 K.

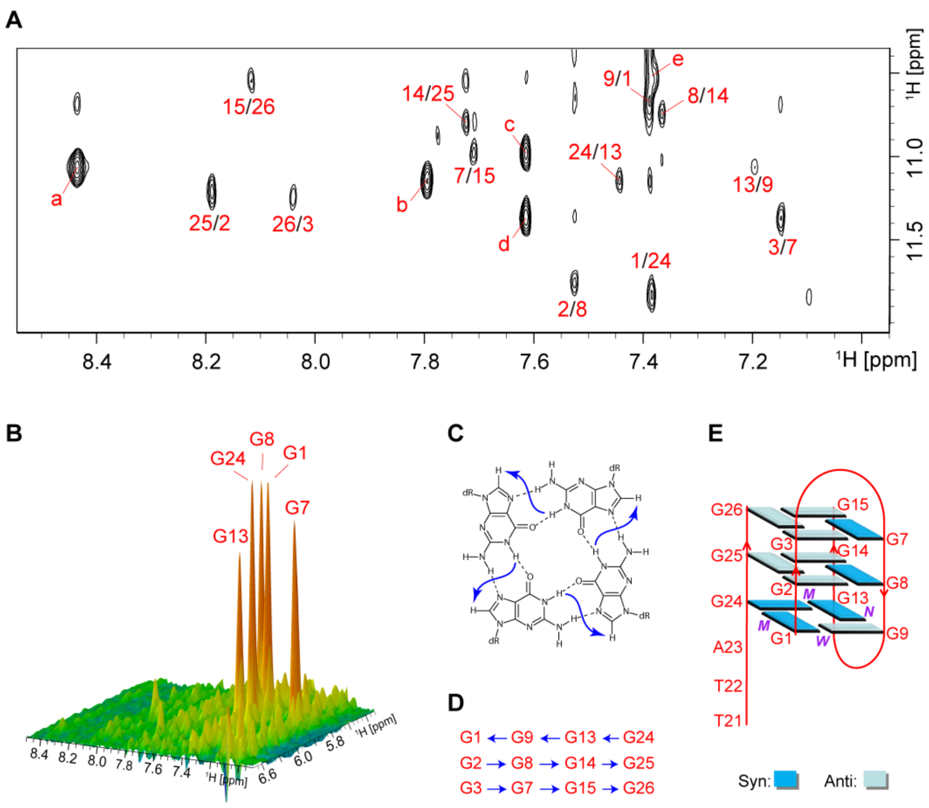

Figure S13. Folding topology of ***htel3ΔT/P3*** determined by NMR. **(A)** NOESY spectrum (250 ms mixing time, 10% D_2O/90% H_2O) shows inter-residue imino H1-base H8 cross peaks to identify the arrangements of G-tetrads. The guanine imino H1-base H8 cross peaks are labelled with residue numbers of imino H1 and base H8 protons in the first and second positions, respectively. The remaining strong cross peaks in the NOE spectrum are also identified as follows: a, G13H1/A12H2; b, G24H1/A23H8; c, G7H1/A6H8; d, G3H1/A6H8; e, G1H21/G24H8. **(B)** Stacked plot of two-dimensional NOESY spectrum (50 ms mixing time, D_2O) of ***htel3ΔT/P3***, distinctively shows the strong intra-residue H8-H1′ cross peaks for the *syn* guanines. **(C)** G-tetrad arrangement with characteristic imino-H8 NOE pattern indicated with arrows. **(D)** Three G-tetrads of ***htel3ΔT/P3*** are shown. The hydrogen-bond directionality within the same G-tetrad is indicated by blue arrows, from a donor (arrow tail) to an acceptor (arrow head). **(E)** Schematic folding topology of ***htel3ΔT/P3*** is shown. The backbones are shown as red solid lines to represent the adoption of RLP-form GQ. *Syn* and *anti* guanines are indicated by dark blue and light blue rectangles, respectively. Experimental conditions: 3 mM DNA, 20 mM Na-Pi buffer (pH 6.8), 100 mM NaCl, and 303 K.

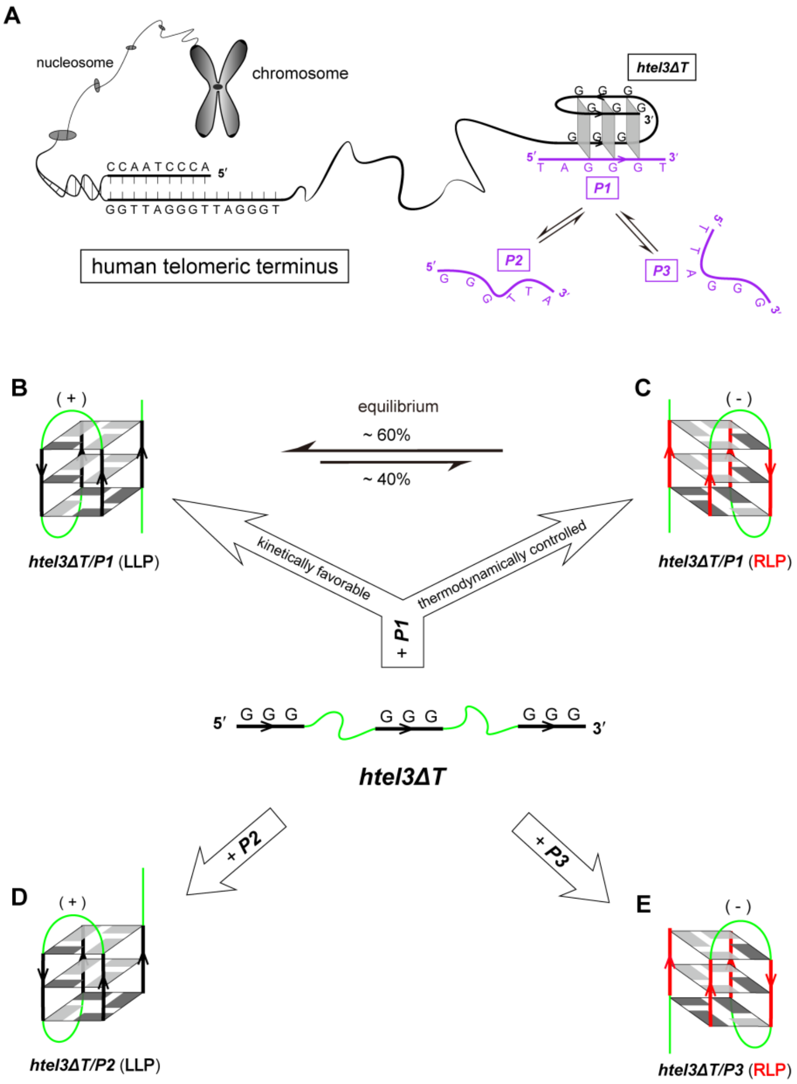

Figure S14. **(A)** Schematic illustration of the formation of heteromolecular GQ at the 3′-terminal of human telomeric DNA G-overhang. The three-repeat of human telomeric DNA fragment as a target (***htel3ΔT***) is recognised by a short G-rich probe of the single-repeat of human telomeric fragment to assemble a hetero-GQ. The competitions between short probe analogues of ***P1***, ***P2*** and ***P3*** (all coloured purple) enable mutual strand displacements when they bind to the same target ***htel3ΔT***. Different outcomes are illustrated in the assembly of the same target ***htel3ΔT*** with analogous probes of ***P1***, ***P2***, and ***P3***. An equimolar ***P1*** associates with ***htel3ΔT*** to yield two distinct hetero-GQs in opposite loop progressions, kinetically favourable LLP-form of ***htel3ΔT/P1*** **(B)**, and the thermodynamically controlled RLP-form of ***htel3ΔT/P1*** **(C)**. When equilibrium is reached, LLP-form of ***htel3ΔT/P1*** comprises approximately 60% populated and RLP-form of ***htel3ΔT/P1*** approximately 40%. On the other hand, the association of ***htel3ΔT*** with either ***P2*** or ***P3*** yields only a single hetero-GQ. The folding topology of GQ complex ***htel3ΔT/P2*** **(D)** is the same as the LLP-form of ***htel3ΔT/P1***, whereas GQ complex ***htel3ΔT/P3*** **(E)** adopts the same topology as the RLP-form of ***htel3ΔT/P1***. In the formation/disassembly processes of LLP-form and RLP-form hetero-GQs, the corresponding LLP-triplex and RLP-triplex are proposed as the most plausible candidates of on-pathway intermediates adopted by the same target sequence of ***htel3ΔT***. The backbones of the LLP-form and RLP-form are coloured black and red, respectively. Loops are coloured green, and left loop progressions are indicated by (+), while the right loop progressions are indicated by (-).

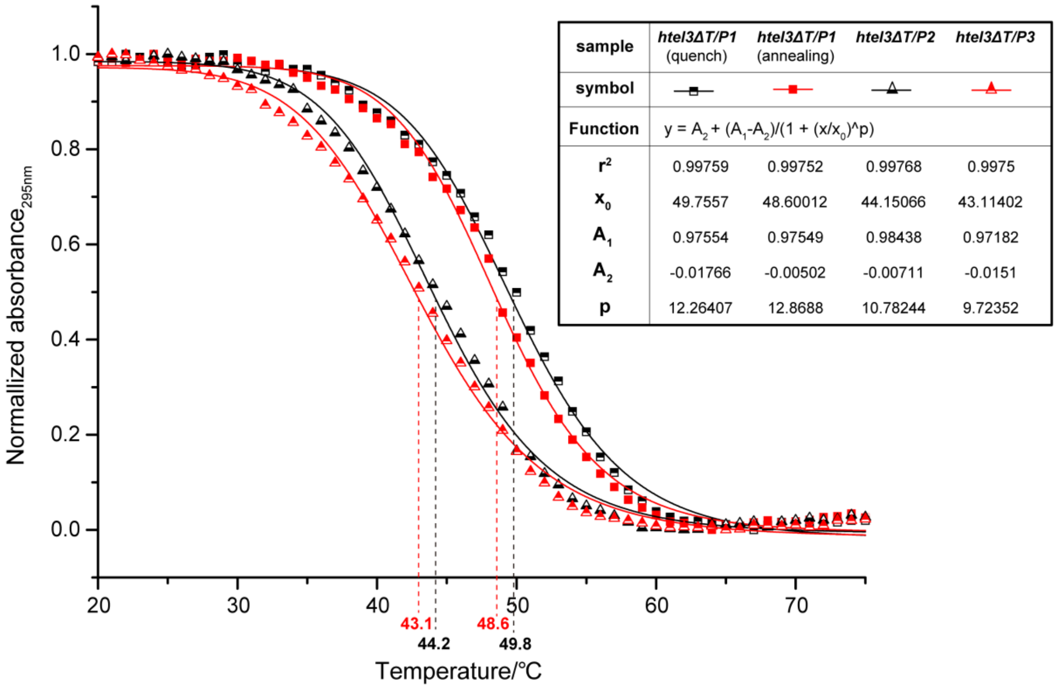

Figure S15. The temperature profiles of UV absorbance at 295 nm for every 85 µM samples of ***htel3ΔT/P1*** (immediately after quick quench), ***htel3ΔT/P1*** (after slow annealing), ***htel3ΔT/P2***, and ***htel3ΔT/P3*** in a buffer of 100 mM NaCl, 20 mM Na-Pi (pH 6.8); these examined samples are indicated by black squares, red squares, black triangles, and red triangles respectively. The vertical axis is a relative absorbance normalized between absorbances at the lowest and the highest temperatures. The fit parameters used to analyze the melting curves are listed in the inset. A_1 and A_2 are the maximum and minimum value, respectively. X_0 is the temperature corresponds to the midpoint of the sigmoidal curve, which is the melting temperature (Tm). The Tm values are indicated by dashed lines.

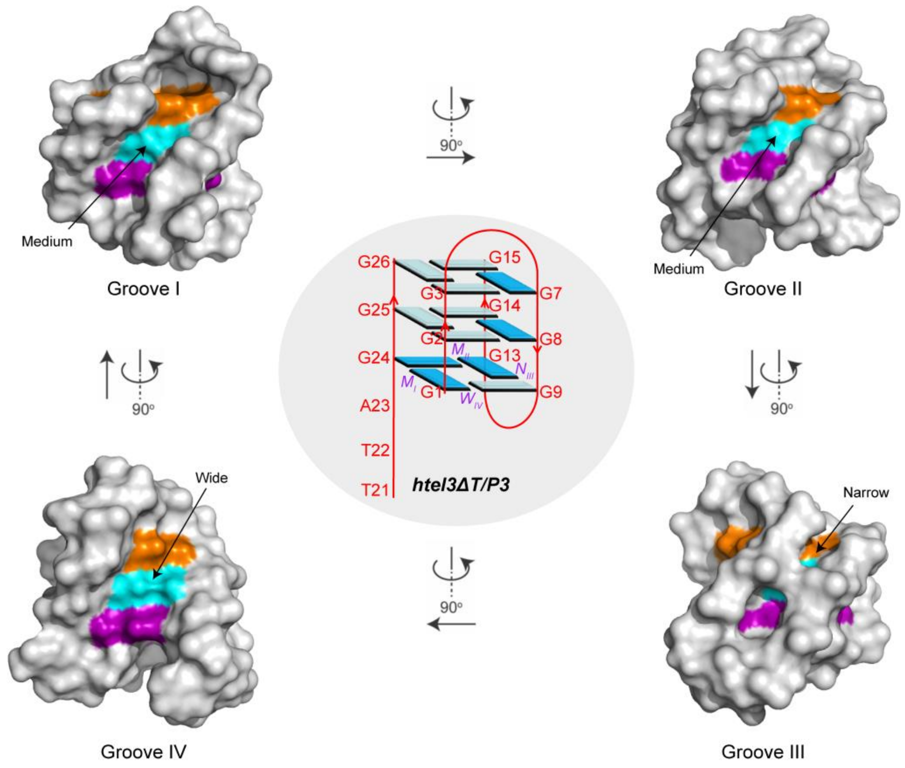

Figure S16. Surface representations of ***htel3ΔT*/*P3***. The planes of top, middle, and bottom G-tetrads are coloured orange, cyan, and purple, respectively. Four grooves are labelled the same as the schematic topology of ***htel3ΔT*/*P3*** in the centre. W, M, and N represent wide groove width, medium groove width, and narrow groove width, respectively. I, II, III, and IV indicate groove I, groove II, groove III, and groove IV, respectively.

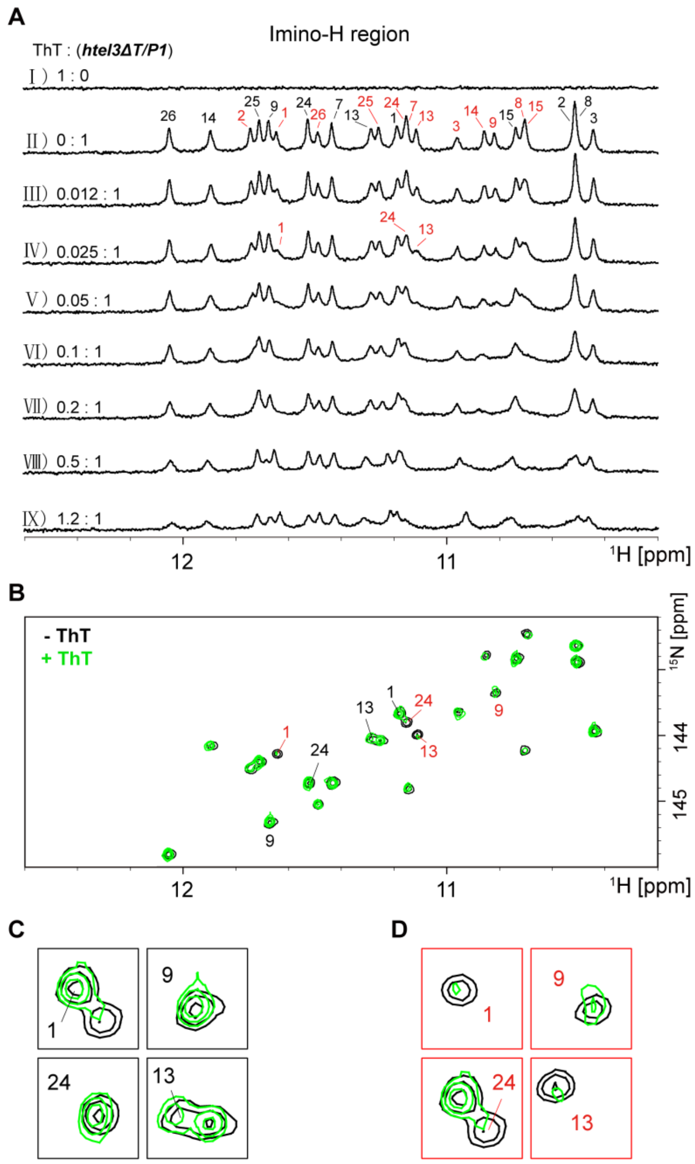

Figure S17. **(A)** Expanded imino proton regions of one-dimensional ^{1}H NMR spectra were recorded for 0.4 mM ***htel3ΔT*/*P1*** in 20 mM Na-Pi (pH 6.8), 100 mM NaCl solution with 0~1.2 equivalent ThT titrations at 288 K. The imino peaks that displayed the most obvious variations, either in chemical shifts or signal broadenings, are marked in spectrum (IV). The imino proton assignments are labelled in spectrum (II). **(B)** ^{1}H-^{15}N HSQC spectra of 6 mM ***htel3ΔT*/*P1*** were recorded in 20 mM Na-Pi (pH 6.8), 100 mM NaCl solution that were titrated without (coloured black) and with 0.025 equivalent (coloured green) ThT at 288K. The imino H1-N1 cross peaks of (G1·G24·G13·G9) G-tetrad plane are labelled in **(B)**. The most obvious variations of H1-N1 cross peaks were observed for (G1·G24·G13·G9) G-tetrad of RLP-form that were particularly magnified in **(D)**. No evident variations for those of LLP-form were magnified in **(C)**. The residue numbers of LLP-form and RLP-form of ***htel3ΔT*/*P1*** are coloured black and

red, respectively.

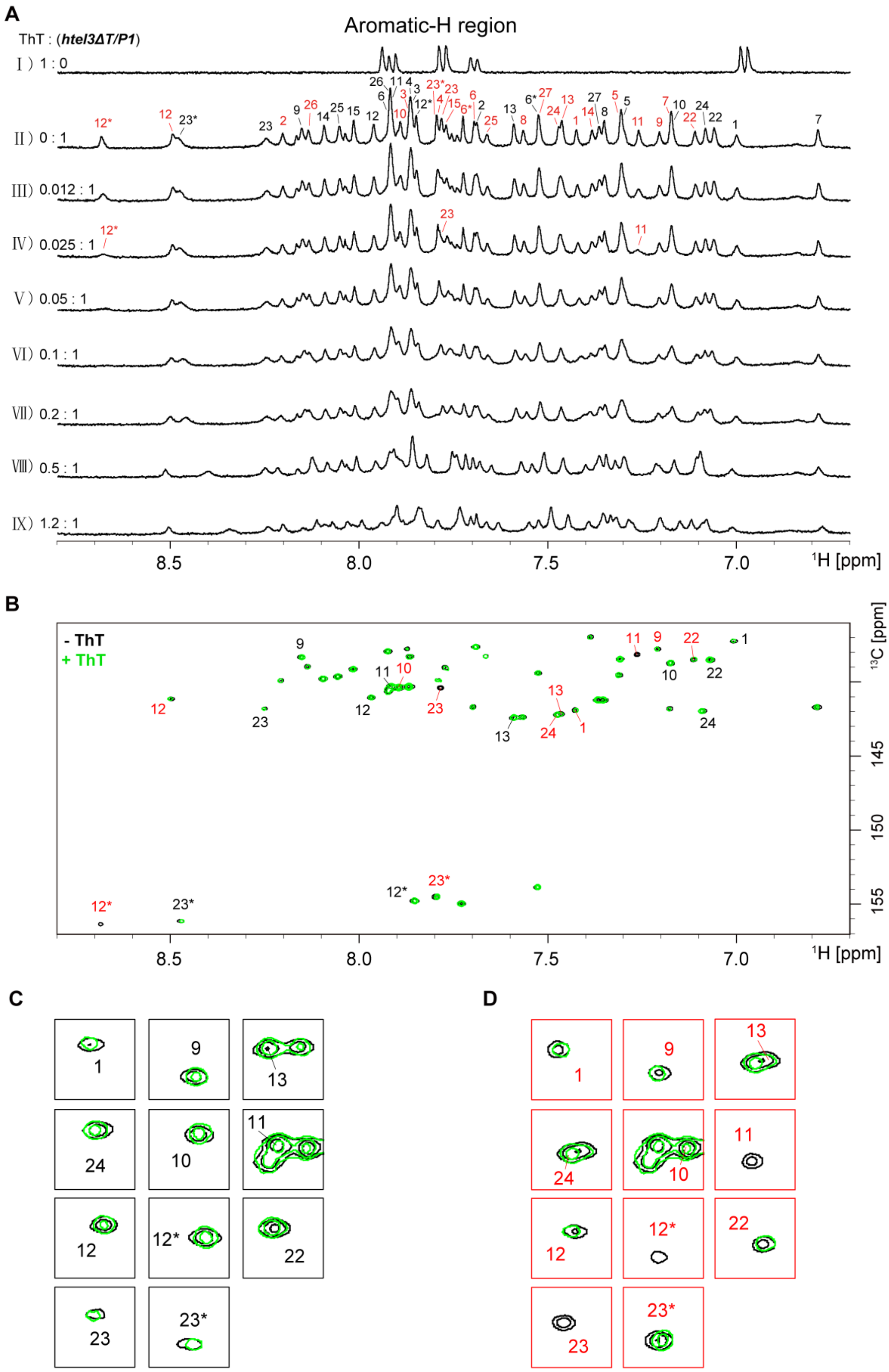

Figure S18. **(A)** Expanded aromatic base proton regions of one-dimensional ^{1}H NMR spectra were recorded for 0.4 mM ***htel3ΔT*/*P1*** in 20 mM Na-Pi (pH 6.8), 100 mM NaCl solution with 0~1.2 equivalent ThT titrations at 288 K. The aromatic base peaks that displayed the most obvious variations, either in chemical shifts or signal broadenings, are marked in spectrum (IV). The base proton assignments are labelled in spectrum (II). The H2 protons of adenine are marked by right superscript *. **(B)**

^{1}H-^{13}C HSQC spectra of 6 mM ***htel3ΔT*/*P1*** were recorded in 20 mM Na-Pi (pH 6.8), 100 mM NaCl solution that were titrated without (coloured in black) and with 0.025 equivalent (coloured in green) ThT at 288 K. The non-exchangeable cross peaks between base protons and their directly bonded base carbons of (G1·G24·G13·G9) G-tetrad plane, loop T10T11A12, and surrounding overhanging residues of T22 and A23 for both LLP-form and RLP-form of ***htel3ΔT*/*P1*** are labelled in **(B)**. The residue numbers of LLP-form and RLP-form of ***htel3ΔT*/*P1*** are coloured black and red, respectively. The obvious variations of these non-exchangeable cross peaks of binding pocket residues immediately proximal to the bound ThT were particularly observed for the RLP-form of ***htel3ΔT*/*P1*** and were distinctively magnified in **(D)**. No evident variations for those of LLP-form of ***htel3ΔT*/*P1*** were magnified in **(C)** as a control comparison.

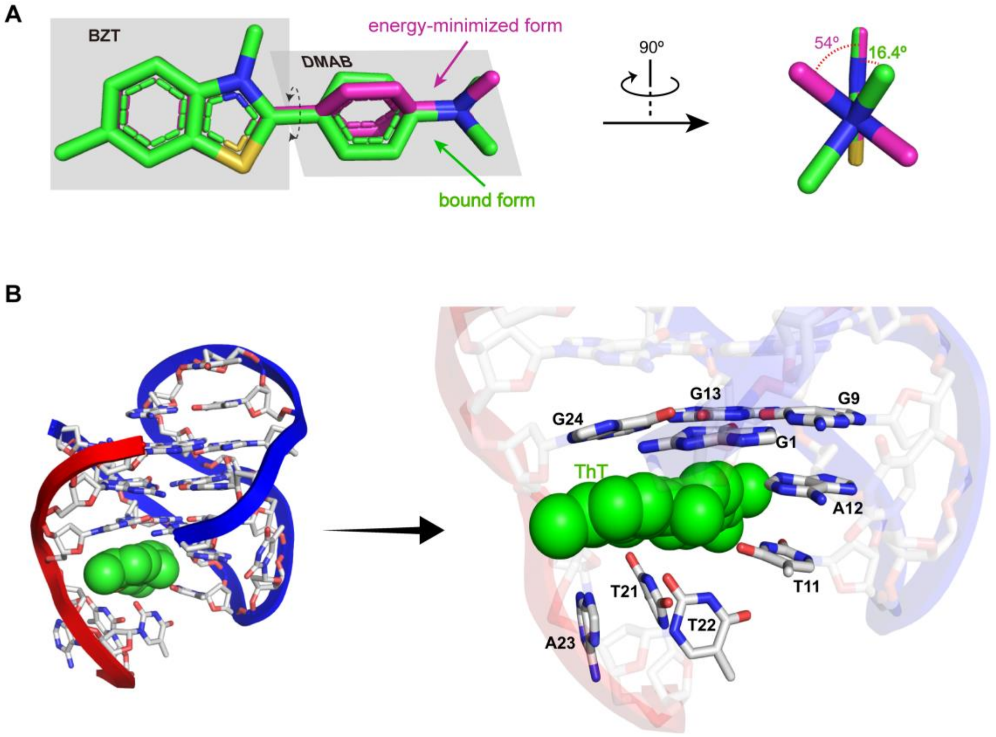

Figure S19. **(A)** Comparison of ring system coplanarity between overlapped structures of free and bound forms of ligand thioflavin T (ThT). Bound form of ThT (green) was extracted from MD simulation complex structure. The free form of ThT (purple) was energy-minimized using the LigPrep module of Schrodinger suites 2018(14). The dihedral angles between the plane BZT and plane DMAB are indicated for the free and bound forms of ligand ThT, respectively. **(B)** Snapshots of GQ target ***htel3ΔT*/*P3*** and ligand ThT after a 150 ns MD simulation. GQ target of ***htel3ΔT*/*P3*** and ligand ThT are represented as cartoon-sticks and spheres, respectively. The particular region adjacent to ThT is magnified on the right.

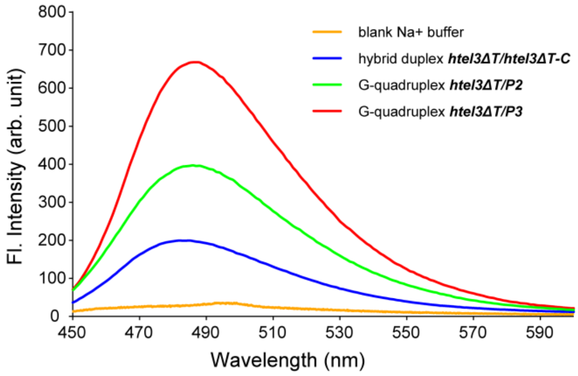

Figure S20. Fluorescence enhancement experiments of 0.05 equivalent ThT upon interaction with DNA samples. Fluorescence spectra of 0.05 mM duplex hybridized between ***htel3ΔT*** and ***htel3ΔT***-C (coloured blue), 0.05 mM ***htel3ΔT***/***P2*** (coloured green), and 0.05 mM ***htel3ΔT***/***P3*** (coloured red) in 20 mM Na-Pi, 100 mM NaCl buffer in H_2O (as blank background, coloured orange). The excitation wavelength (λ_{ex}) was 425 nm.

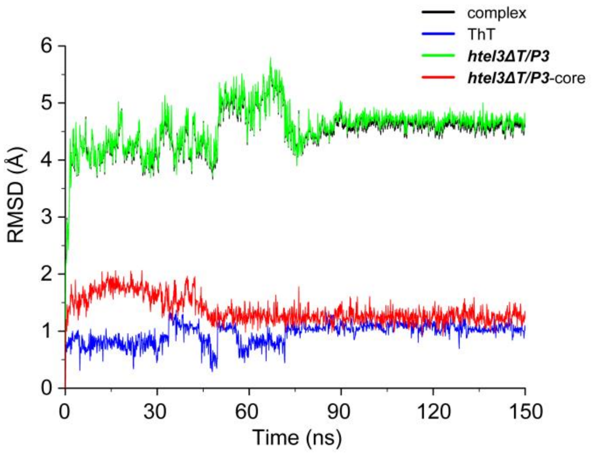

Figure S21. RMSDs of the heavy atoms of the GQ target-ligand complex between ***htel3ΔT***/***P3*** and ThT during the 150 ns simulation. Four curves represent the RMSDs on the entire complex between ***htel3ΔT***/***P3*** and ThT (black), specifically on ThT (blue), specifically on the GQ target of ***htel3ΔT***/***P3*** (green), and specifically on the core parts (red) of ***htel3ΔT***/***P3***, as indicated at the upper right corner with respect to their starting structure.

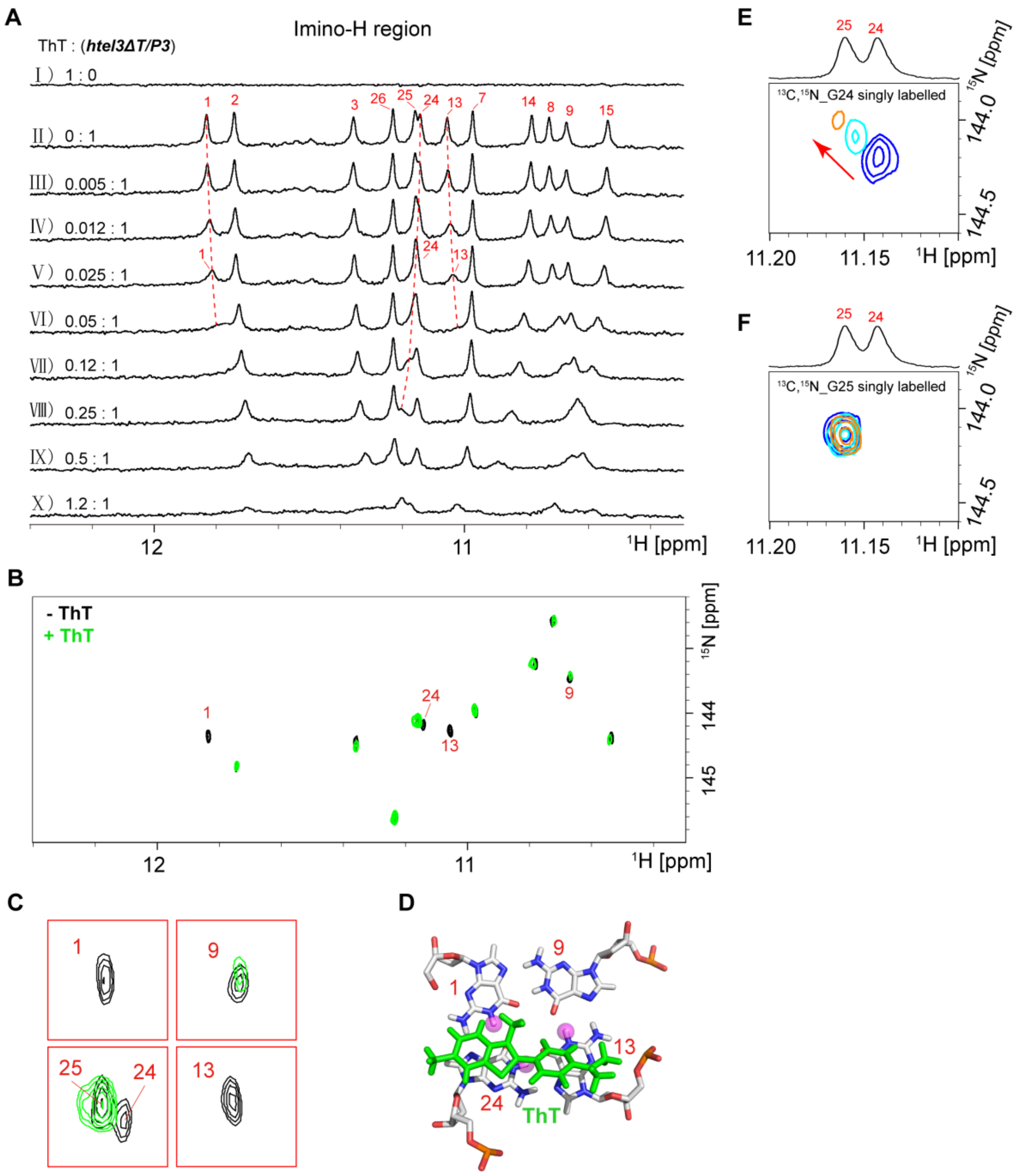

Figure S22. **(A)** Expanded imino proton regions of one-dimensional ^{1}H NMR spectra were recorded for 0.2 mM ***htel3ΔT/P3*** in 20 mM Na-Pi (pH 6.8), 100 mM NaCl solution with 0~1.2 equivalent ThT titrations at 288 K. The imino peaks that displayed the most obvious variations, either in chemical shifts or signal broadenings, are marked in spectrum (V). The imino proton assignments are labelled in spectrum (II). The residue numbers of ***htel3ΔT/P3*** are coloured red to represent the adoption of RLP-form. The moving trend of chemical shift perturbation preferentially for the peaks belonging to G1, G13 and G24 are marked by dotted line. **(B)** ^{1}H-^{15}N HSQC spectra of 3 mM ***htel3ΔT/P3*** were recorded in 20 mM Na-Pi (pH 6.8), 100 mM NaCl solution that were titrated without (coloured black) and with 0.025 equivalent (coloured green) ThT at 288 K. The imino H1-N1 cross peaks of (G1·G24·G13·G9) G-tetrad plane are labelled. **(C)** The most obvious variations of H1-N1 cross peaks were observed for (G1·G24·G13·G9) G-tetrad of ***htel3ΔT/P3*** that were particularly magnified. **(D)** Stacking of ThT (coloured green) over (G1·G24·G13·G9) G-tetrad. The cross peaks with evident variation in the chemical shift perturbation are marked by purple spherical shadows. ^{15}N-filtered spectra of 0.1 mM singly guanine-labelled ***htel3ΔT-G24/P3*** (**E**) and ***htel3ΔT-G25/P3*** (**F**) titrated with 0 equivalent (coloured blue), 0.012 equivalent (coloured cyan) and 0.05 equivalent (coloured orange) of ThT at 288 K. Singly guanine-labelled samples were 100% ^{15}N, ^{13}C-labelled at positions G24 and G25. The movement of chemical shift

pertubation of G24 upon ThT titration isdirected by red arrow. The imino proton assignments of G24 and G25 in one-dimensional ^{1}H spectrum collected using non-labelled samples are shown on the top inset of each 2D ^{1}H-^{15}N HSQC spectra using singly guanine-labelled samples in (**E**) and (**F**).

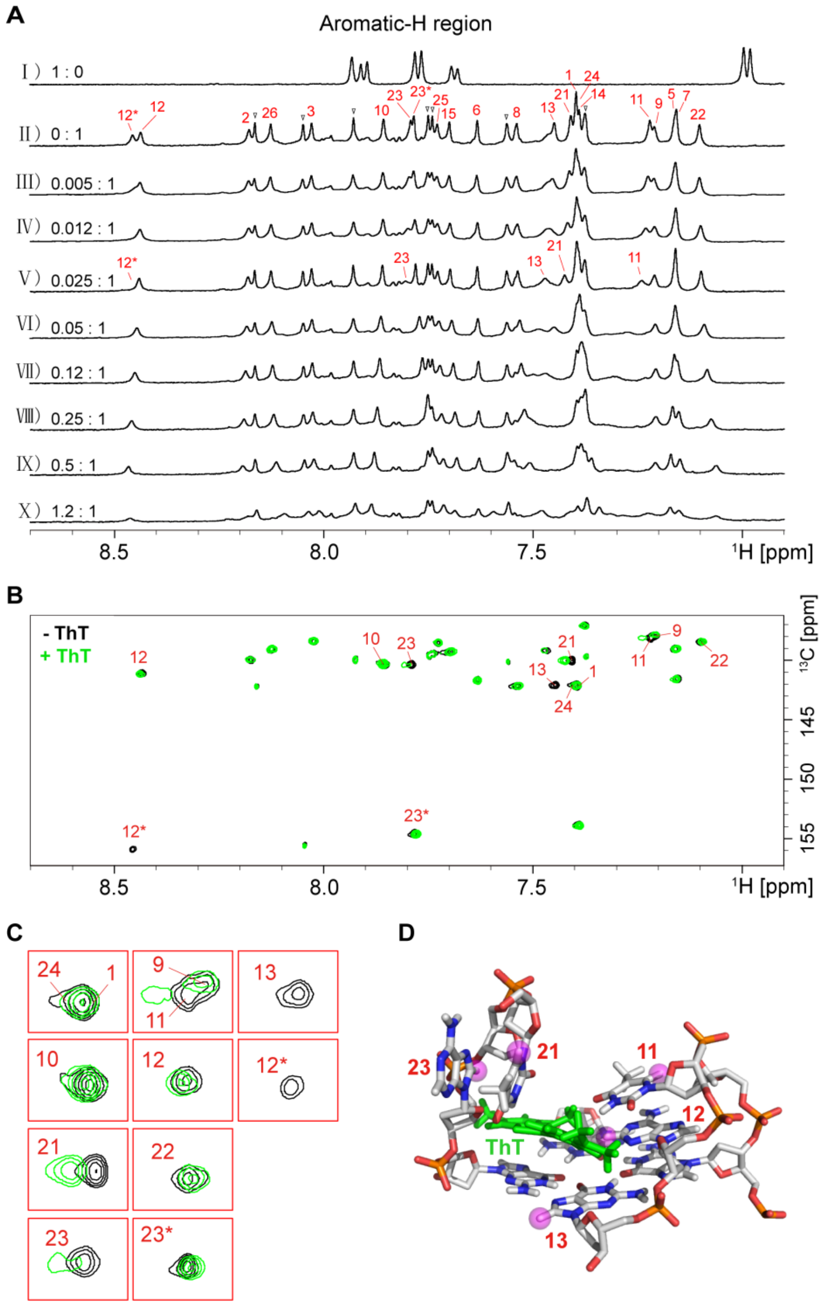

Figure S23. **(A)** Expanded aromatic base proton regions of one-dimensional ^{1}H NMR spectra were recorded for 0.4 mM ***htel3ΔT*/*P3*** in 20 mM Na-Pi (pH 6.8), 100 mM NaCl solution with 0~1.2 equivalent ThT titrations at 288 K. The aromatic base peaks that displayed the most obvious variations, either in chemical shifts or signal broadenings, are marked in spectrum (V). The base proton assignments are labelled in spectrum (II). The H2 protons of adenine are marked by right superscript *. The signals that belong to the unfolded portion of ***P3*** are marked by inverted triangles. **(B)** ^{1}H-^{13}C HSQC spectra of 3 mM ***htel3ΔT*/*P3*** were recorded in 20 mM Na-Pi (pH 6.8), 100 mM NaCl solution that were titrated without (coloured black) and with 0.025

equivalent (coloured green) ThT at 288 K. The obvious variations of non-exchangeable cross peaks between base protons and their directly bonded base carbons were mostly observed for (G1·G24·G13·G9) G-tetrad plane, flanking loop T10T11A12, and surrounding overhanging residues of T21, T22, and A23 that were are immediately proximal to the bound ThT. These H8/H6/H2/H2*-C8/C6/C2/C2* cross peaks are labelled in **(B)** and distinctively magnified in **(C)**. **(D)** Stacking of ThT (coloured green) over (G1·G24·G13·G9) G-tetrad, loop T10T11A12, and overhanging residues of T21, T22, and A23. The cross peaks with evident variation in the chemical shift perturbation are denoted by purple spherical shadows. The residue numbers of ***htel3ΔT*/*P3*** are coloured red to represent the adoption of RLP-form GQ.

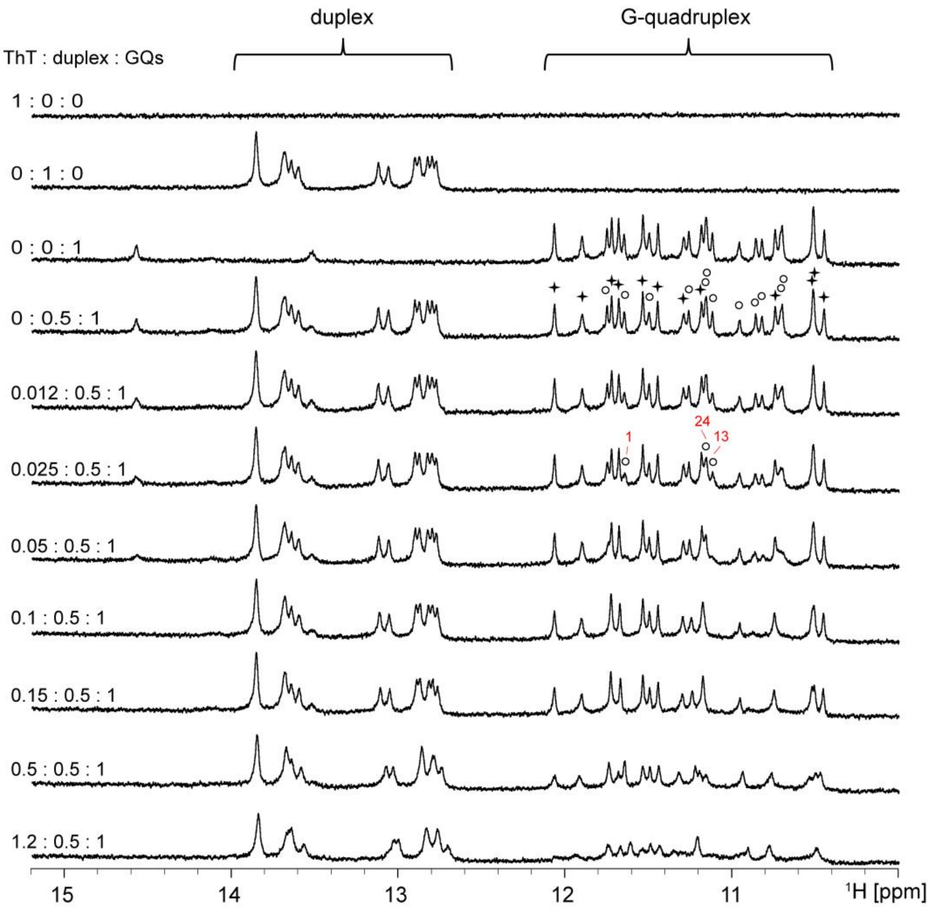

Figure S24. Comparison of ThT binding specificity between hetero-GQs and corresponding telomeric DNA duplex context. One-dimensional ^{1}H NMR spectra were recorded in a mixture of 0.4 mM hetero-GQs of ***htel3ΔT*/*P1*** (10.5~12 ppm) and 0.2 mM duplex context of ***htel3ΔT*/*htel3ΔT-C*** (12.6~14 ppm) upon 0~1.2 equivalent of ThT titrations. LLP-form and RLP-form of ***htel3ΔT*/*P1*** GQs are denoted by asterisks and circles, respectively. The peaks with evident variation are marked by residue numbers in red to represent the RLP-form of ***htel3ΔT*/*P1***. Experimental conditions: 20 mM Na-Pi buffer (pH 6.8), 100 mM NaCl, and 288 K.

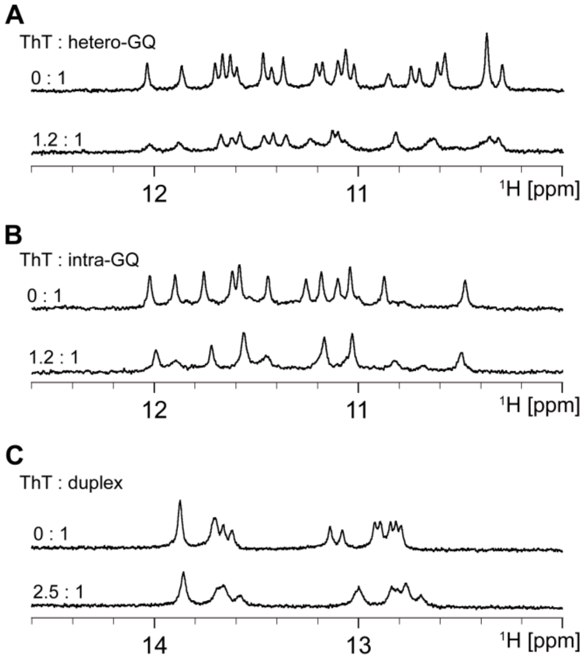

Figure S25. The expanded imino proton region of one-dimensional ^{1}H NMR spectra of 0.4 mM hetero-GQs (**A**, ***htel3ΔT/P1***), 0.2 mM intra-GQs (**B**, ***2GKU***, listed in Table S3), and 0.2 mM duplex (**C**, ***htel3ΔT/htel3ΔT-C***) were titrated with ThT in high equivalent. Experimental conditions: 20 mM Na-Pi buffer (pH 6.8), 100 mM NaCl, and 288 K.

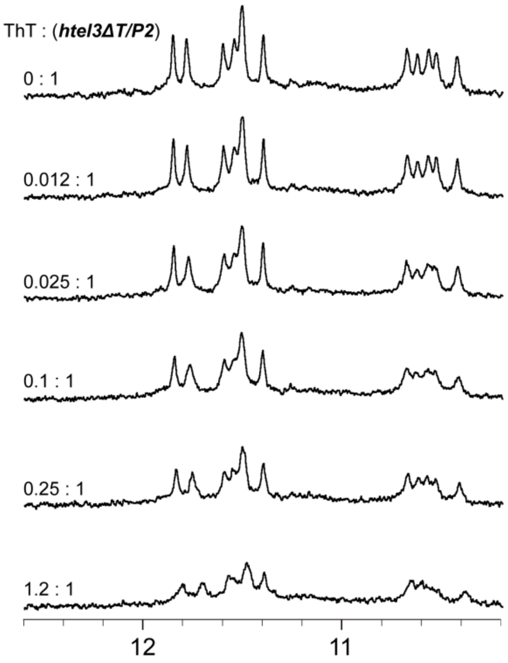

Figure S26. Expanded imino proton regions of one-dimensional ^{1}H NMR spectra were recorded for 0.2 mM ***htel3ΔT/P2*** in 20 mM Na-Pi (pH 6.8), 100 mM NaCl solution, titrated with 0~1.2 equivalent of ThT at 288 K.

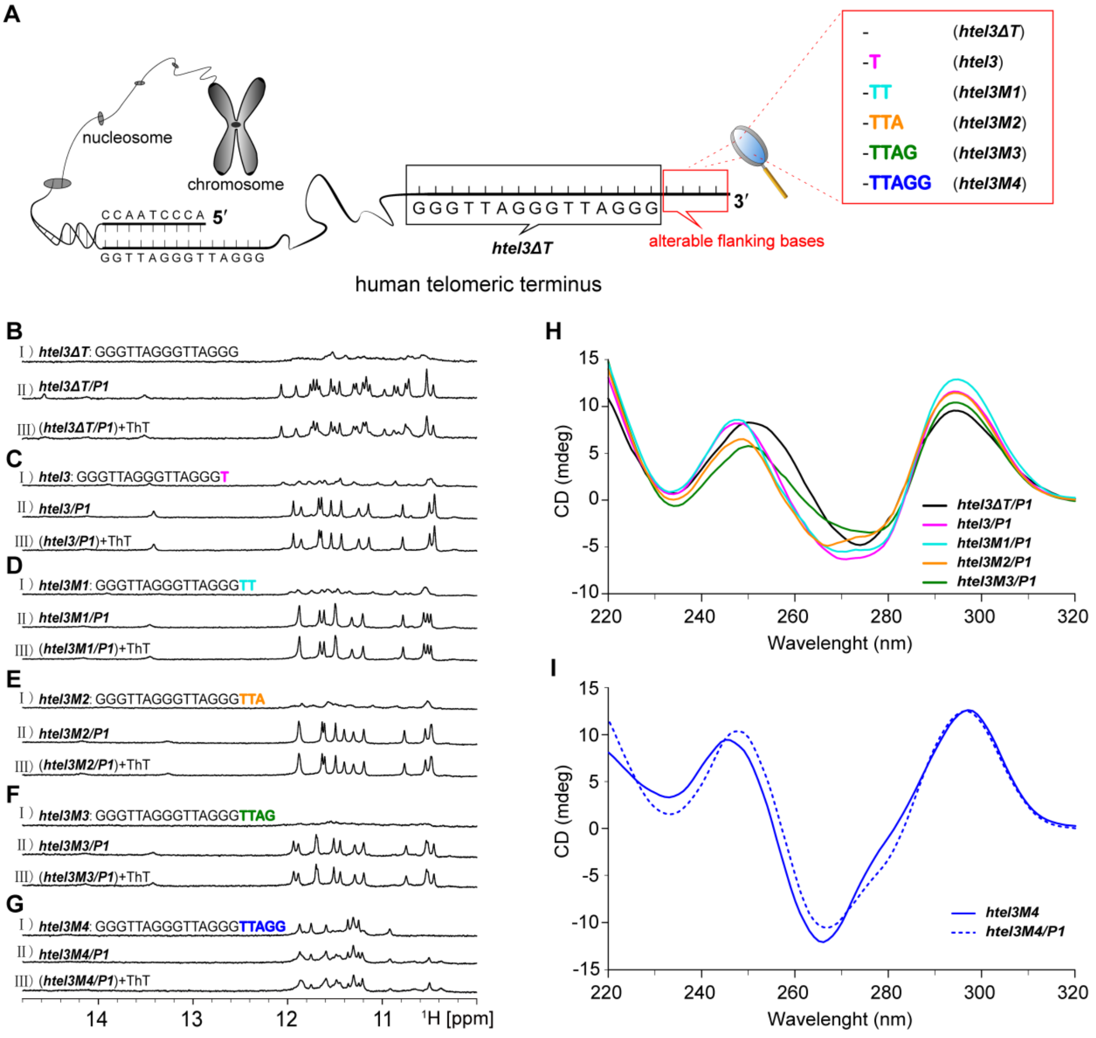

Figure S27. **(A)** Illustration of naturally occurring variations of the 3′-flanking bases at human telomere terminus. Each has a specific permutation of the 5′-GGGTTA-3′ repeat. The total of six different endings of target sequence d(GGGTTAGGGTTAGGG) at 3′ terminus of human telomere are named as follows: only a bare ending without a flanking base (***htel3ΔT***), with flanking bases T (magenta, ***htel3***), with TT (cyan, ***htel3M1***), with TTA (orange, ***htel3M2***), with TTAG (green, ***htel3M3***), and with TTAGG (blue, ***htel3M4***). **(B-G)** Expanded imino proton region of one-dimensional ^{1}H spectra for individual target sequence variants with different 3′-endings alone I), hetero-GQs formed with the same probe ***P1*** II), and these hetero-GQs titrated with 0.05 equivalent of ThT III) are shown. **(H)** CD spectra of ***htel3ΔT/P1***, ***htel3***/***P1***, ***htel3M1***/***P***, ***htel3M2***/***P1***, and ***htel3M3***/***P1*** are coloured black, magenta, cyan, orange, and green, respectively. **(I)** CD spectra recorded for ***htel3M4*** and ***htel3M4***/***P1*** complex are labelled with blue solid and dashed line, respectively.

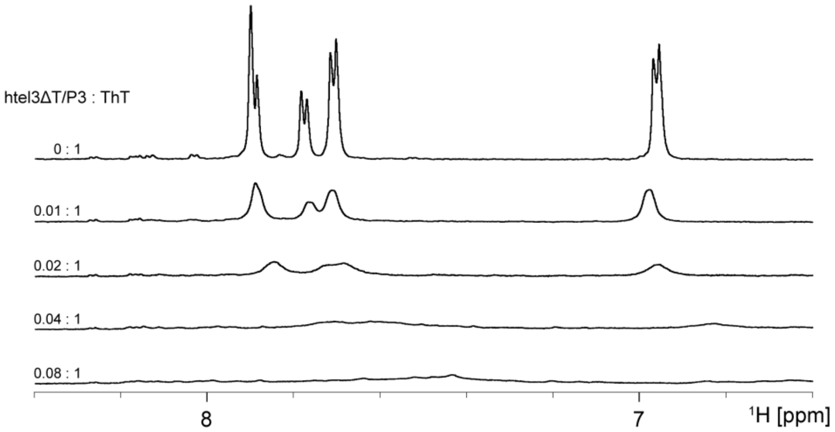

Figure S28. Expanded aromatic proton regions of one-dimensional ^{1}H NMR spectra were recorded for 10 mM ThT in 20 mM Na-Pi (pH 6.8), 100 mM NaCl solution titrated with 0~0.08 equivalent of GQ target ***htel3ΔT/P3*** at 288 K.

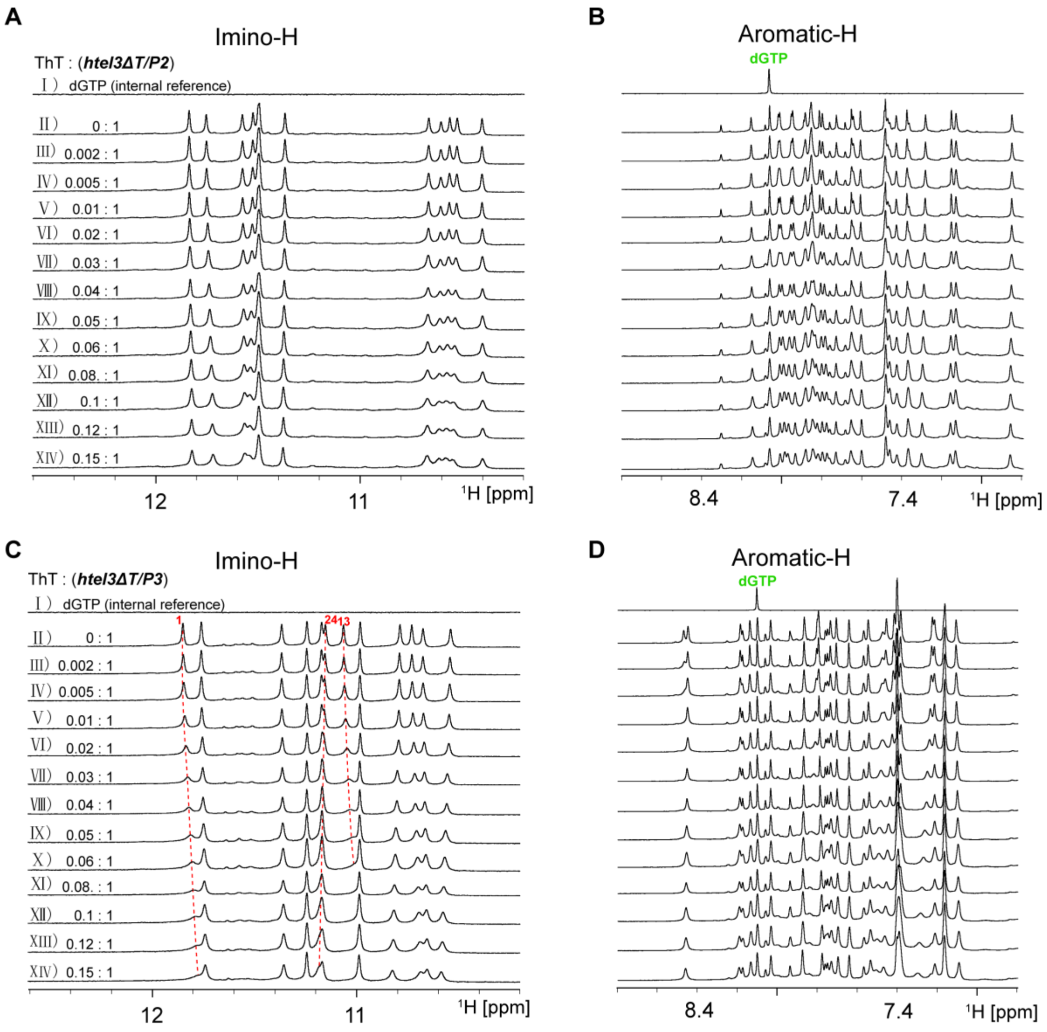

Figure S29. Expanded imino and aromatic proton regions of one-dimensional ^{1}H NMR spectra were recorded for 1 mM hetero-GQs ***htel3ΔT*/*P2*** (**A, B**) and ***htel3ΔT*/*P3*** (**C, D**) in 20 mM Na-Pi (pH 6.8), 100 mM NaCl solution with 0~0.15 low equivalent of ThT titrations at 288 K. The imino peaks that displayed the most obvious variations, either in chemical shifts or

signal broadenings, were marked in spectrum (**C**-II). The moving trend of signal pertubations specifically belongs to G1, G13 and G24 of hetero-GQ ***htel3ΔT/P3*** were marked by dotted line. Another 1 mM dGTP (coloured green) was added to the titration system as an internal reference.

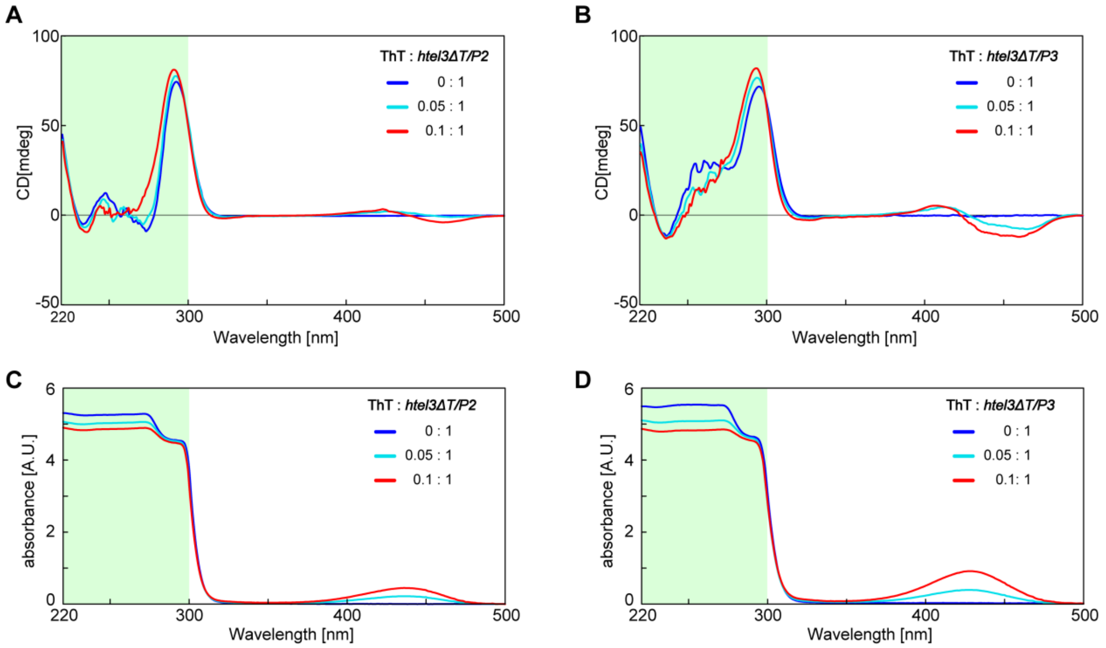

Figure S30. The ICD spectra of 0.5 mM hetero-GQs ***htel3ΔT/P2*** (**A**) and ***htel3ΔT/P3*** (**B**) are titrated with 0 equivalent (blue), 0.05 equivalent (cyan) and 0.1 equivalent (red) of ThT, respectively. The corresponding UV absorption spectra reveal that both UV and CD signals of hetero-GQs ***htel3ΔT/P2*** (**C**) and ***htel3ΔT/P3*** (**D**) specifically at 260~300 nm encounter signal overflow (coloured in green background), but the collections of ThT signals at 400~500 nm are not affected by the excessive DNA GQ signal overflow.

Supplementary Tables

Table S1. ^{1}H, ^{13}C, ^{15}N chemical shift (ppm) of ***htel3ΔT/P3***.[a]

Res.	H1/H3	H8/H6	H2/Me	H21, H22	H1′	H2′, H2″	H3′	H4′	G,A-C8 T-C6	G-C5 A-C2	G-N1
**G1**	11.82	7.38	–	10.44, 6.33	5.89	2.87, 2.89	4.98	4.33	142.03	118.37	144.35
**G2**	11.73	8.18	–	10.03, 6.67	5.68	2.61, 2.64	5.09	4.34	139.94	117.35	144.80
**G3**	11.35	8.03	–	8.74, 7.14	6.10	2.66, 2.70	5.02	4.23	138.36	117.18	144.45
**T4**	–[b]	7.44	1.76	–	6.00	2.14, 2.40	4.69	3.96	139.13	–	–
**T5**	–[b]	7.14	1.64	–	5.83	1.65, 2.03	4.45	–[c]	139.00	–	–
**A6**	–	7.61	7.35	–	5.64	1.82, 2.41	4.61	3.85	141.66	153.89	–
**G7**	10.96	7.14	–	9.01, 6.68	5.97	3.16, 3.56	4.98	–[c]	141.53	119.18	143.98
**G8**	10.72	7.52	–	9.39, 6.88	5.74	2.52, 2.66	4.97	–[c]	142.14	118.69	142.54
**G9**	10.66	7.19	–	9.74, 6.68	5.77	2.56, 2.85	5.11	4.34	137.83	116.28	143.43
**T10**	–[b]	7.84	1.97	–	6.38	2.43, 2.53	4.97	4.43	140.30	–	–
**T11**	–[b]	7.21	1.51	–	5.80	0.99, 1.81	4.72	4.15	138.05	–	–
**A12**	–	8.42	8.44	–	6.56	2.84, 3.04	5.13	4.62	141.06	155.92	–
**G13**	11.04	7.43	–	8.19, 7.68	6.13	3.02, 3.78	5.01	4.50	142.07	118.49	144.28
**G14**	10.77	7.36	–	9.23, 5.86	5.93	2.36, 2.75	4.80	4.39	137.02	117.60	143.22
**G15**	10.52	7.70	–	9.25, 6.39	6.32	2.51, 2.52	4.67	4.39	139.22	117.04	144.37
**T21**	–[b]	7.39	1.56	–	5.93	2.18, 2.31	4.62	4.01	140.01	–	–
**T22**	–[b]	7.09	1.50	–	5.83	1.96, 2.30	4.59	4.10	138.40	–	–
**A23**	–	7.77	7.76	–	6.24	1.86, 2.46	4.69	4.13	140.32	154.60	–
**G24**	11.13	7.37	–	9.20, 6.83	6.07	2.90, 3.55	4.91	4.46	142.14	118.76	144.15
**G25**	11.14	7.72	–	9.52, 6.42	6.00	2.61, 2.68	5.08	4.45	138.51	117.06	144.09
**G26**	11.22	8.11	–	8.41, 7.75	6.52	2.54, 2.72	4.39	4.26	139.04	117.79	145.59

[a]The values of ^{1}H chemical shift were obtained at 303K, 20 mM sodium phosphate buffer (pH 6.8), 100 mM NaCl, and 4 mM sample strand concentration.
[b]This proton cannot be observed due to fast exchange with water.
[c]This proton cannot be distinguished because it overlaps with other cross-peaks.
– The residue does not have this atom.

Table S2. Primers for enzymatic synthesis (presented 5′-3′)

Name	Sequence (5′-3′)
htel3ΔT-G15-F	GGGTTAGGGTTAGG
htel3ΔT-G15-R	CCCTAACCCTAACCCTTTTTT
P1-G3-F	AGTGCACTGC/rG/TA
P1-G3-R	CTACGCAGTGCACTTTTTT
P1-G4-F	AGTGCACTGC/rG/TAG
P1-G4-R	CCTACGCAGTGCACTTTTTT
P1-G5-F	AGTGCACTGC/rG/TAGG
P1-T6-R	ACCCTACGCAGTGCACTTTTTT
P2-G1-F	AGTGCACTGC/rG/
P2-G1-R	CCGCAGTGCACTTTTTT
P2-G2-F	AGTGCACTGC/rG/G
P2-G2-R	CCCGCAGTGCACTTTTTT
P2-G3-F	AGTGCACTGC/rG/GG
P2-A6-R	TAACCCCGCAGTGCACTTTTTT
P3-G4-F	AGTGCACTGC/rG/TTA
P3-G4-R	CTAACGCAGTGCACTTTTTT
P3-G5-F	AGTGCACTGC/rG/TTAG
P3-G5-R	CCTAACGCAGTGCACTTTTTT
P3-G6-F	AGTGCACTGC/rG/TTAGG
P3-G6-R	CCCTAACGCAGTGCACTTTTTT

Table S3. Titration results of intramolecular GQs with low concentration of ThT [a]

**PDB ID** and Sequence	Topology	Significance	Models	Titrated with ThT
(***1XAV***)(15) TGAGGGTGGGTAGGGTGGGTAA	parallel	Human c-MYC promoter	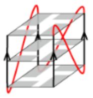	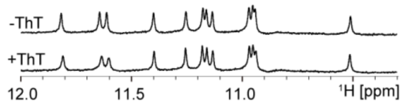
(***2KYP***)(16) CGGGCGGGCGCGAGGGAGGGG	parallel	Human cKIT-2 proto-oncogene promoter	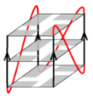	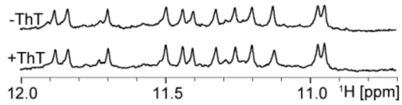
(***148D***)(17) GGTTGGTGTGGTTGG	antiparallel	Thrombin-binding DNA aptamer	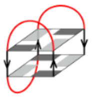	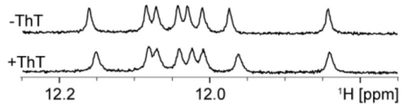
(***143D***)(18) AGGGTTAGGGTTAGGGTTAGGG	antiparallel	Human telomeric repeat	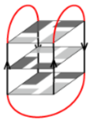	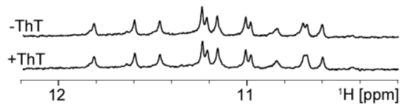
(***2GKU***)(19) TTGGGTTAGGGTTAGGGTTAGGGA	(3+1) hybridized form 1	Human telomeric repeat	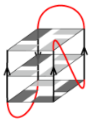	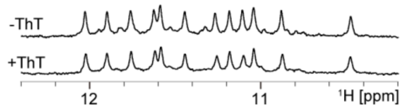
(***2JSM***)(20) TAGGGTTAGGGTTAGGGTTAGGG	(3+1) hybridized form 1	Human telomeric repeat	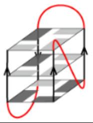	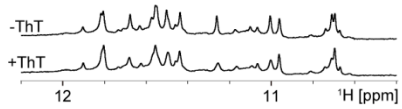
(***186D***)(21) TTGGGGTTGGGGTTGGGGTTGGGG	(3+1) hybridized form 2	Tetrahymena telomeric repeat	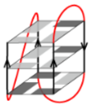	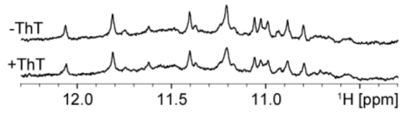
(***6AC7***)(22) TGGGGTCCGAGGCGGGGCTTGGG	(3+1) hybridized GQ with a bulge	Poly (ADP-ribose) polymerase 1 promoter	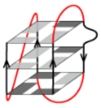	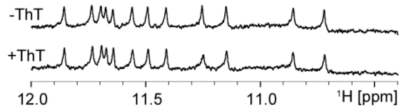

[a] 0.2 mM intramolecular GQs are titrated with 0.05 equivalent ThT. The temperature is 288K.

References

1. Wang, J., Cheng, M., Chen, J., Ju, H., Monchaud, D., Mergny, J.L. and Zhou, J. (2020) An oxidatively damaged G-quadruplex/hemin DNAzyme. *Chem Commun (Camb)*, **56**, 1839-1842.
2. Phan, A.T. and Patel, D.J. (2003) Two-repeat human telomeric d(TAGGGTTAGGGT) sequence forms interconverting parallel and antiparallel G-quadruplexes in solution: distinct topologies, thermodynamic properties, and folding/unfolding kinetics. *J. Am. Chem. Soc.*, **125**, 15021-15027.
3. Rauzan, B., McMichael, E., Cave, R., Sevcik, L.R., Ostrosky, K., Whitman, E., Stegemann, R., Sinclair, A.L., Serra, M.J. and Deckert, A.A. (2013) Kinetics and thermodynamics of DNA, RNA, and hybrid duplex formation. *Biochemistry*, **52**, 765-772.
4. Gray, R.D., Trent, J.O., Arumugam, S. and Chaires, J.B. (2019) Folding Landscape of a Parallel G-Quadruplex. *J Phys Chem Lett*, **10**, 1146-1151.

5. Gray, R.D., Trent, J.O. and Chaires, J.B. (2014) Folding and unfolding pathways of the human telomeric G-quadruplex. *J. Mol. Biol.*, **426**, 1629-1650.
6. Green, J.J., Ying, L., Klenerman, D. and Balasubramanian, S. (2003) Kinetics of unfolding the human telomeric DNA quadruplex using a PNA trap. *J. Am. Chem. Soc.*, **125**, 3763-3767.
7. Lane, A.N., Chaires, J.B., Gray, R.D. and Trent, J.O. (2008) Stability and kinetics of G-quadruplex structures. *Nucleic Acids Res.*, **36**, 5482-5515.
8. Vorlickova, M., Kejnovska, I., Sagi, J., Renciuk, D., Bednarova, K., Motlova, J. and Kypr, J. (2012) Circular dichroism and guanine quadruplexes. *Methods*, **57**, 64-75.
9. Randazzo, A., Spada, G.P. and da Silva, M.W. (2013) Circular dichroism of quadruplex structures. *Top. Curr. Chem.*, **330**, 67-86.
10. Calabrese, D.R., Chen, X., Leon, E.C., Gaikwad, S.M., Phyo, Z., Hewitt, W.M., Alden, S., Hilimire, T.A., He, F., Michalowski, A.M. *et al.* (2018) Chemical and structural studies provide a mechanistic basis for recognition of the MYC G-quadruplex. *Nat Commun*, **9**, 4229.
11. Neidle, S. (2017) Quadruplex nucleic acids as targets for anticancer therapeutics. *Nat. Rev. Chem.*, **1**.
12. Dai, J., Carver, M., Hurley, L.H. and Yang, D. (2011) Solution structure of a 2:1 quindoline-c-MYC G-quadruplex: insights into G-quadruplex-interactive small molecule drug design. *J. Am. Chem. Soc.*, **133**, 17673-17680.
13. Wirmer-Bartoschek, J., Bendel, L.E., Jonker, H.R.A., Grun, J.T., Papi, F., Bazzicalupi, C., Messori, L., Gratteri, P. and Schwalbe, H. (2017) Solution NMR Structure of a Ligand/Hybrid-2-G-Quadruplex Complex Reveals Rearrangements that Affect Ligand Binding. *Angew. Chem.*, **56**, 7102-7106.
14. Sastry, G.M., Adzhigirey, M., Day, T., Annabhimoju, R. and Sherman, W. (2013) Protein and ligand preparation: parameters, protocols, and influence on virtual screening enrichments. *J. Comput. Aided Mol. Des.*, **27**, 221-234.
15. Ambrus, A., Chen, D., Dai, J., Jones, R.A. and Yang, D. (2005) Solution structure of the biologically relevant G-quadruplex element in the human c-MYC promoter. Implications for G-quadruplex stabilization. *Biochemistry*, **44**, 2048-2058.
16. Kuryavyi, V., Phan, A.T. and Patel, D.J. (2010) Solution structures of all parallel-stranded monomeric and dimeric G-quadruplex scaffolds of the human c-kit2 promoter. *Nucleic Acids Res.*, **38**, 6757-6773.
17. Schultze, P., Macaya, R.F. and Feigon, J. (1994) Three-dimensional solution structure of the thrombin-binding DNA aptamer d(GGTTGGTGTGGTTGG). *J. Mol. Biol.*, **235**, 1532-1547.
18. Wang, Y. and Patel, D.J. (1993) Solution structure of the human telomeric repeat d[AG3(T2AG3)3] G-tetraplex. *Structure*, **1**, 263-282.
19. Luu, K.N., Phan, A.T., Kuryavyi, V., Lacroix, L. and Patel, D.J. (2006) Structure of the human telomere in K+ solution: an intramolecular (3 + 1) G-quadruplex scaffold. *J. Am. Chem. Soc.*, **128**, 9963-9970.
20. Phan, A.T., Kuryavyi, V., Luu, K.N. and Patel, D.J. (2007) Structure of two intramolecular G-quadruplexes formed by natural human telomere sequences in K+ solution. *Nucleic Acids Res.*, **35**, 6517-6525.
21. Wang, Y. and Patel, D.J. (1994) Solution structure of the Tetrahymena telomeric repeat d(T2G4)4 G-tetraplex. *Structure*, **2**, 1141-1156.
22. Sengar, A., Vandana, J.J., Chambers, V.S., Di Antonio, M., Winnerdy, F.R., Balasubramanian, S. and Phan, A.T. (2019) Structure of a (3+1) hybrid G-quadruplex in the PARP1 promoter. *Nucleic Acids Res.*, **47**, 1564-1572.
